# Molecular Approach to the Identification of Fish in the South China Sea

**DOI:** 10.1371/journal.pone.0030621

**Published:** 2012-02-17

**Authors:** Junbin Zhang, Robert Hanner

**Affiliations:** 1 Department of Aquaculture & Life Science, Shanghai Ocean University, Shanghai, China; 2 Department of Integrative Biology, University of Guelph, Guelph, Ontario, Canada; University of Vermont, United States of America

## Abstract

**Background:**

DNA barcoding is one means of establishing a rapid, accurate, and cost-effective system for the identification of species. It involves the use of short, standard gene targets to create sequence profiles of known species against sequences of unknowns that can be matched and subsequently identified. The Fish Barcode of Life (FISH-BOL) campaign has the primary goal of gathering DNA barcode records for all the world's fish species. As a contribution to FISH-BOL, we examined the degree to which DNA barcoding can discriminate marine fishes from the South China Sea.

**Methodology/Principal Findings:**

DNA barcodes of cytochrome oxidase subunit I (COI) were characterized using 1336 specimens that belong to 242 species fishes from the South China Sea. All specimen provenance data (including digital specimen images and geospatial coordinates of collection localities) and collateral sequence information were assembled using Barcode of Life Data System (BOLD; www.barcodinglife.org). Small intraspecific and large interspecific differences create distinct genetic boundaries among most species. In addition, the efficiency of two mitochondrial genes, 16S rRNA (16S) and cytochrome *b* (cyt*b*), and one nuclear ribosomal gene, 18S rRNA (18S), was also evaluated for a few select groups of species.

**Conclusions/Significance:**

The present study provides evidence for the effectiveness of DNA barcoding as a tool for monitoring marine biodiversity. Open access data of fishes from the South China Sea can benefit relative applications in ecology and taxonomy.

## Introduction

Fishes show an astonishing diversity of shapes, sizes, and colors. The delimitation and recognition of fish species is not only of interest for taxonomy and systematists, but it is also a requirement in studies of natural history and ecology, fishery management, tracking the dispersal patterns of eggs and larvae, estimations of recruitment and spawn areas, and authentication of food products [1]–[2]. Fish identification is traditionally based on morphological features. However, due to high diversity and morphological plasticity, in many cases, fish and their diverse developmental stages are difficult to identify by using morphological characteristics alone [2]. DNA-based identification techniques have been developed and proven to be analytically powerful [3]–[5]. As a standardized and universal method, DNA barcoding identification systems have been widely advocated to identify species and uncover biological diversity in these years [6]–[7]. For many animal taxa, sequence divergences within the 5′ region of the mitochondrial cytochrome oxidase subunit I (COI) gene are generally much greater between species than within species. This in turn suggests that the approach is extensively applicable among phylogenetically distant animal groups [8]–[14]. Many studies have shown that intraspecific variation of COI barcodes is generally pretty small and clearly discriminable from interspecific variation [15]–[24].

The South China Sea lies within the Indo-West Pacific marine biogeographic province, which has long been recognized as the global center of marine tropical biodiversity [25]. In addition to temperate species, there are many coral fish living in the South China Sea. The most striking feature of these marine fish is their diversity, both in terms of number of species and in the range of morphologies [26]. In the present study, more than 1,300 specimens from the South China Sea were sequenced for COI barcodes. DNA barcode data were then integrated with the relevant taxonomical and ecological information in two projects, Fishes from the South China Sea (FSCS) and Coral Fishes from the South China Sea (CFCS), in the Barcode of Life Data System (BOLD).

Recently, some other mitochondrial genes or nuclear ribosomal DNA fragments, have been proposed as alternatives for species identification [5], [27]. Most studies focus on narrow-range taxa, but only a few have systematically compared the utility of different molecular markers in species identification. Herein, we also used samples from a few select groups of species to test three other different molecular markers—mitochondrial cytochrome *b* (cyt*b*), 16S rRNA gene (16S), and nuclear ribosomal 18S rRNA gene (18S)—with respect to their ability to identify fish species.

## Materials and Methods

### Ethics

Ethical approval was not required for this study because no endangered fish were involved. However, specimen collection and maintenance were performed in strict accordance with the recommendations of Animal Care Quality Assurance in China.

### Specimen collection and DNA extraction

**Figure 1:**
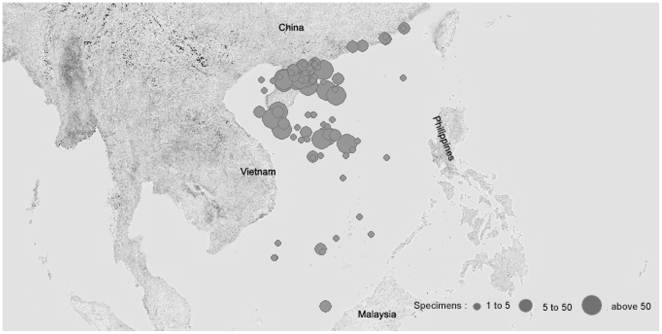
Map showing the sampling localities for fish from the South China Sea.

Fish samples were collected from more than 40 locations in the South China Sea (Fig. 1, Table S1). Voucher specimens were deposited in the Marine Biodiversity Collection of South China Sea, South China Sea Institute of Oceanography, Chinese Academy of Sciences. All specimens were preserved in 70% ethanol. Tissue samples were dissected from the body muscle, and genomic DNA was extracted according to the standard Barcode of Life protocol [28].

### PCR and DNA sequencing

Fragments of the 5′ region of mitochondrial COI gene were amplified using C_FishF1t1/C_FishR1t1 primer cocktails [29]. The primer combination C_FishF1t1 contained two primers (FishF2_t1/VF2_t1), and C_FishR1t1 also contained two primers (FishR2_t1/FR1d_t1). These primers are described in Table S2. PCR reactions were carried out in 96-well plates using Mastercycler® Eppendorf gradient thermal cyclers (Brinkmann Instruments, Inc.). The reaction mixture of 825 µl water, 125 µl 10× buffer, 62.5 µl MgCl_2_ (25 mM), 6.25 µl dNTP (10 mM), 6.25 µl of each primer (0.01 mM), and 6.25 µl Taq DNA polymerase (5 U/µl) was prepared for each plate. Each well contained 10.5 µl of mixture and 2 µl genomic DNA. Thermocycling comprised an initial step of 2 min at 95°C and 35 cycles of 30 s at 94°C, 40 s at 52°C, and 1 min at 72°C, with a final extension at 72°C for 10 min. Amplicons were visualized on 2% agarose E-Gel® 96-well system (Invitrogen). Each chosen PCR product was sequenced bi-directionally with the primers M13F (5′-TGTAAAACGACGGCCAGT-3′) and M13R (5′-CAGGAAACAGCTATGAC-3′) using the BigDye® Terminator v.3.1 Cycle Sequencing Kit (PE Biosystems, Inc.). Thermocycling conditions were as follows: An initial step of 2 min at 96°C and 30 cycles of 30 s at 96°C, 15 s at 55°C, and 4 min at 60°C. Final PCR products were directly sequenced using an ABI 3730 capillary sequencer according to manufacturer's instructions.

For specimens that failed to yield amplification products using the primer combinations above, a second round of PCR using the alternative C_VF1LFt1/C_ VR1LRt1 primer combination was carried out. C_VF1LFt1 consisted of four primers (VF1_t1/VF1d_t1/LepF1_t1/VFli_t1), and C_VR1LRt1 also comprised four primers (VR1_t1/VR1d_t1/LepR1_t1/VRli_t1) (Table S2). The PCR program consisted of an initial denaturation at 94°C for 1 min, five cycles of 94°C for 30 s, annealing at 50°C for 40 s, and extension at 72°C for 1 min, followed by 30 cycles of 94°C for 30 s, 54°C for 40 s, and 72°C for 1 min, with a final extension at 72°C for 10 min. All other procedures were performed as given above.

Specimen data such as images, collection information, museum accession numbers, and sequence trace files were assembled in BOLD in accordance with the BARCODE data standard as specified by the Consortium for the Barcode of Life in collaboration with the International Nucleotide Sequence Database Collaboration (INSDC) [30], [31]. Sequences were submitted to GenBank using the NCBI Barcode Submission Tool, where they were subsequently annotated with the reserved keyword BARCODE.

In addition to the COI barcode region, two DNA fragments, one of mitochondrial 16S and one of cyt*b*, and one DNA fragment of nuclear ribosomal 18S were screened as potential species markers in 282 specimens from 52 species. Primers utilized in this study are listed in Table S2. Each PCR reaction mixture contained of 16.7 µl water, 2.5 µl 10× buffer, 2.0 µl MgCl_2_ (25 mM), 1 µl dNTPs (10 mM), 0.5 µl each primer (0.01 mM), 0.2 µl Taq DNA polymerase (5 U/µl) and 1.0 µl template DNA. PCR amplifications were performed with the following conditions: 35 cycles of denaturation at 94°C for 45 s, annealing at 52–62°C (depending on the primer combination) for 50 s, and extension at 72°C for 1 min, with an initial denaturation at 94°C for 2 min and final extension at 72°C for 5 min. Amplified products were visualized in 1% agarose gel, and purified products were directly sequenced on an Applied Biosystems 3730 sequencer using the BigDye Terminator Cycle Sequencing Ready Reaction Kit (PE Biosystems, Inc.). Sequencing primers were the same as those listed above for PCR. All sequencing reactions were performed according to the manufacturer's instructions.

### Data analyses

DNA sequences were aligned with SeqScape v.2.1.1 software (Applied Biosystems, Inc.). Mitochondrial COI and cyt*b* sequences were translated into amino acids in order to exclude sequencing errors and to avoid the inclusion of pseudogene sequences in the datasets. Sequence divergences were calculated using the Kimura 2 parameter (K2P) distance [32]. This system usually makes a suitable metric model when genetic distances are low [33]. An unrooted NJ tree based on K2P distances was created using MEGA software (version 3.1) [34].

The following categories of K2P distances were calculated: intraspecific distances, interspecific values within the same genus, and interspecific values between different genera within the same family. These values were plotted using the boxplot representation of R. Boxplots in SPSS 11.5 software (SPSS Inc., Chicago, IL, U.S.) [35]. Separate boxplots were constructed only for families containing specimens from 2 or more genera in order to compare among taxonomic categories. Median (central bar), interquartile range (IQR: between upper [Q3] and low [Q1] quartile), values lying within 1.5× IQR beneath Q1 or 1.5× above Q3 (“whiskers”), and extreme values (outliers) are described in the boxplots.

## Results

### COI DNA barcoding

A total of 1336 bidirectional COI sequences belonging to 242 species were obtained (GenBank accession numbers, taxonomic data and museum numbers listed in Table S1). All sequences were aligned with a consensus length of 652 bp, and no insertions, deletions, or stop codons were observed in any sequence. However, multiple haplotypes were detected for some species.

**Figure 2:**
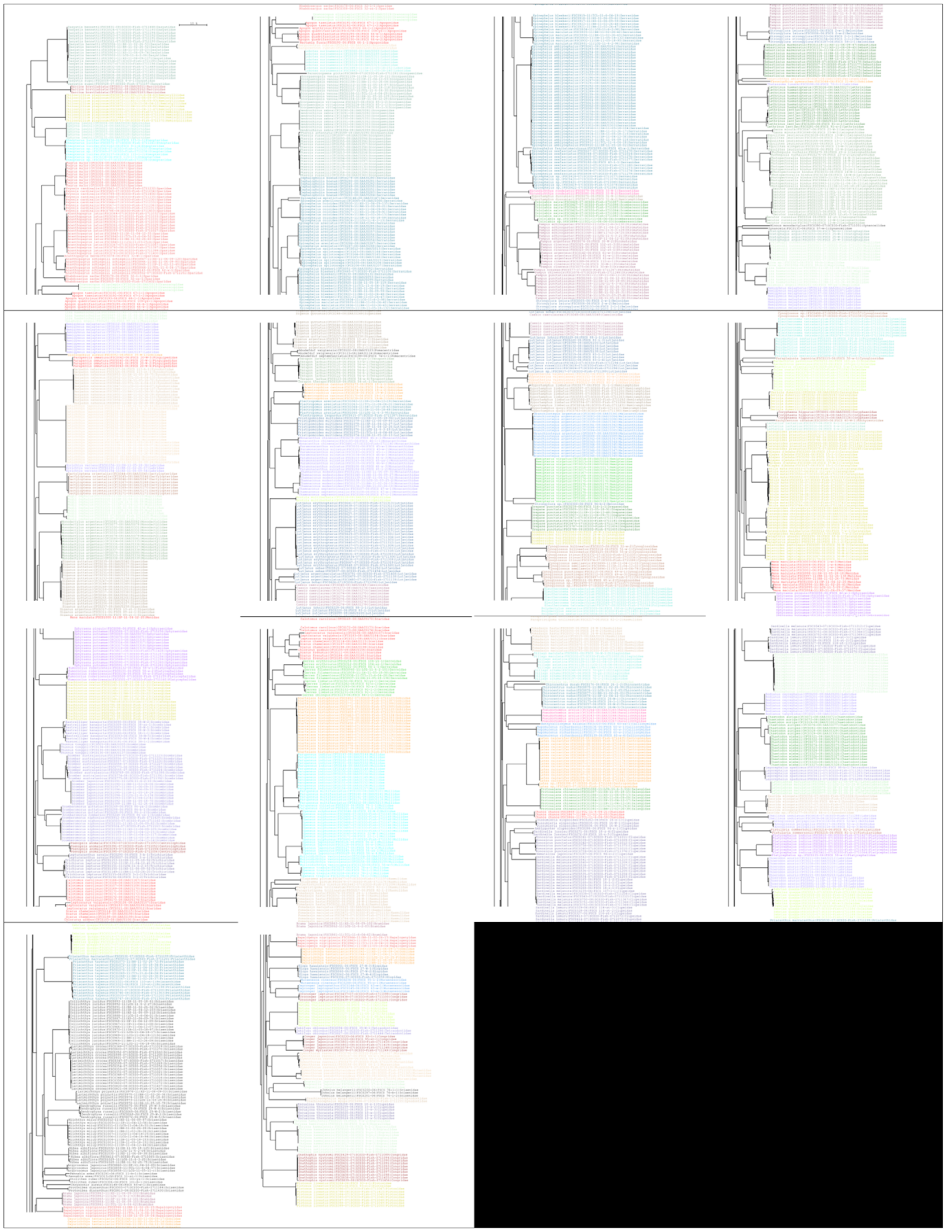
Neighbor-joining (NJ) tree based on COI barcodes from fishes of the South China Sea. Scale: 5% K2P distance. The specimen ID is annotated in each sequence.

**Figure 3:**
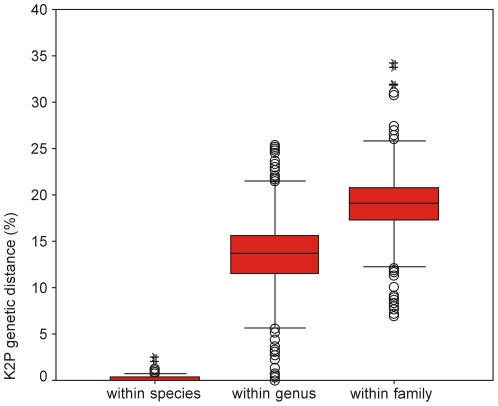
The distribution of K2P distances at COI sequences within species, genus and family respectively. IQR: interval into which the central 50% of the data fall. Black bar in the box indicates the median. Circles indicate mild outliers and asterisks indicate extreme outliers. Extreme outliers are discussed in the text.

**Figure 4:**
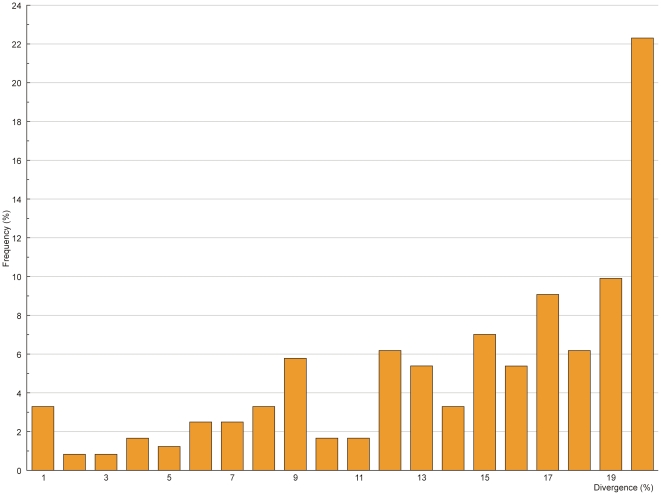
Distribution of the genetic distances to the nearest-neighbor. The analysis is based on all the comparisons of COI barcodes from this study.

The mean intraspecific K2P (Kimura two-parameter) distance was 0.18%. The distance increased sharply to 13.55% among individuals of different congeneric species. Apart from *Pampus*, all other COI sequences formed species clusters [Fig. 2]. Barcode divergences of 1% were used as filters to perform comparisons between units that were identified morphologically; the criterion was met in all cases except *Upeneus sulphureus*, *Siganus guttatus*, *Alepes djedaba*, *Acentrogobius caninus*, *Hyporhamphus limbatus*, *Gymnothorax reevesii*, *Kumococius rodericensis*, *Mene maculata*, *Terapon jarbua*, *Zebrias quagga*, *Pennahia anea*, and *Mugil cephalus*. For these the barcode divergences reached maximum value of 2.51%, and 98.43% (5723 out of 5814) of pairwise genetic distances within species were below 1%. Overall, the average of interspecific distances among congeneric species was over 70-fold higher than that of intraspecific distances. For higher taxonomic ranks (family, order, and class), mean pairwise genetic distances increased gradually, reaching 19.65%, 24.05%, and 24.91%, respectively (Table 1). Interspecific genetic distances below 5% were found only among pairwise comparisons within genera and not at high taxonomic levels such as family or order. The steep increase in genetic variation at the generic level and the smoothness of the rise at high taxonomic levels was observed. This indicates profound differences at species boundaries under the frame of COI divergence (Table 1 and Fig. 3). The distribution of the nearest-neighbor distance (NND), namely the minimum of genetic variation between a species and its closest relative, revealed that only 3.31% of NNDs (8 cases) were lower than 1% (Fig. 4). Fish speciation has many causes, and the rate of mitochondrial COI differentiation during evolution is not equal for all fishes [27]. The distribution of interspecific K2P genetic distances of COI gene within genera at the family level was obviously different (Table 2). Wide fluctuations were observed in values of the interspecific divergence within genera. In the genus *Gerres*, the interspecific distance reached 25.35%, but in the genera *Scomber*, *Thamnaconus*, *Pterois*, *Cololabis*, *Etmopterus*, *Pampus*, and *Plectropomus*, most genetic variations within the genus were below 5%.

**Table 1:** Summary of K2P genetic divergences at different taxonomic levels.

Comparisons within	Taxa	Number of comparisons	Mean	Minimum	Maximum	SE*
species	199	5814	0.18	0	2.51	0.01
genus	141	5958	13.55	0	25.35	0.07
family	80	11126	19.65	6.99	35.71	0.051
order	22	375239	24.05	13.96	39.58	0.01
class	2	429246	24.91	14.74	40.58	0.01

*standard error.

Data are from 1336 sequences from 242 species and 159 genera.

**Table 2:** Distribution of interspecific K2P genetic distances of COI gene within genus at the family level.

Order	Family	<5%	5–10%	>10%
Anguilliformes	Congridae			5
Anguilliformes	Muraenidae	3		24
Aulopiformes	Synodontidae			6
Beloniformes	Belonidae			11
	Hemiramphidae			36
	Scomberesocidae	12		
Clupeiformes	Chirocentridae			8
	Clupeidae			156
	Engraulidae			41
Mugiliformes	Mugilidae			19
Perciformes	Apogonidae			14
	Carangidae			86
	Centropomidae		32	
	Chaetodontidae	52	37	
	Gerreidae			59
	Haemulidae			30
	Labridae			7
	Leiognathidae		15	
	Lethrinidae			56
	Lutjanidae		15	560
	Mullidae			220
	Pomacentridae		2	
	Priacanthidae			28
	Sciaenidae			80
	Scombridae	72		48
	Serranidae		237	3217
	Siganidae			68
	Sillaginidae			32
	Sparidae		111	
	Sphyraenidae			13
	Stromateidae	24	19	236
	Terapontidae			8
Pleuronectiformes	Cynoglossidae		9	81
Rajiformes	Dasyatidae			38
Scorpaeniformes	Platycephalidae	20		34
Squaliformes	Etmopteridae	16		
Tetraodontiformes	Monacanthidae	18		
	Total	217	462	5279

Values of zero have been left blank.

### Genetic analyses of other markers

**Figure 5:**
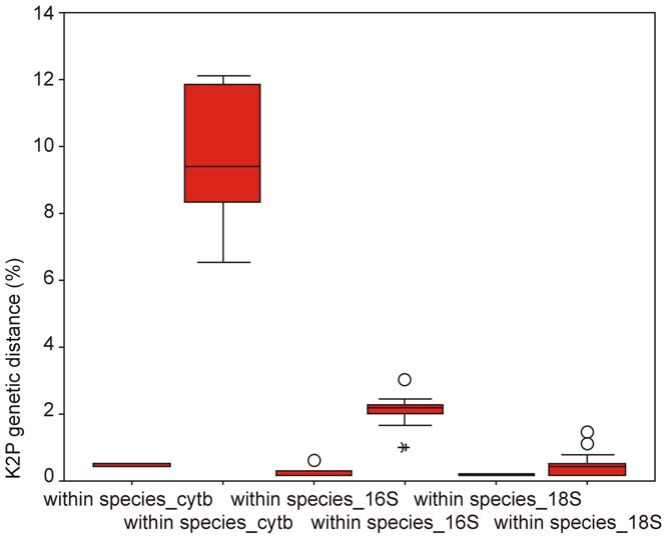
The distribution of intra- and inter- specific genetic divergences for cyt*b*, 16S, and 18S. K2P genetic distances within species and genus for partial sequences from mitochondrial cyt *b*, 16S, and nuclear ribosomal 18S genes of fish from the South China Sea.

**Figure 6:**
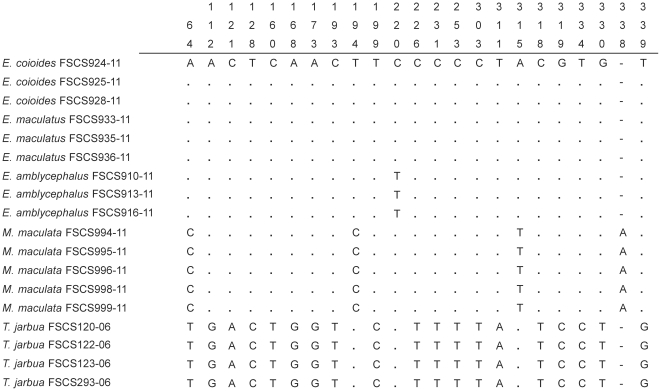
Sequence analyses of 18S for fish identification. Diagnostic sites in 18S for *Epinephelus coioides*, *Epinephelus maculatus*, *Epinephelus amblycephalus*, *Mene maculata*, *Terapon jarbua*, and *Zebrias quagga* as examples of the character-based method.

A high level of sequence variations for cyt*b* makes it difficult to design universal primers for these fish. Thirteen primers were designed for cyt*b* (Table S2), but fewer than half of the samples were amplified successfully. For the 282 selected specimens from 52 species, a data set of 281 mitochondrial 16S (521–561 bp; accession numbers JN211430–JN211710), 124 cyt*b* (832 bp; accession numbers JN211987–JN212110), and 276 nuclear ribosomal 18S (449–459 bp; accession numbers JN211711–JN211986) sequences were ultimately obtained. Many insertions and deletions were found in 16S and 18S. While sequence errors could be detected for cyt*b* by translating into amino acids, the non-coding regions of 16S and 18S could not.The average intraspecific variation was 0.78 for cyt*b* and 0.27 for 16S. Intraspecific K2P distances of 18S were low (the average was only 0.16), and 18S sequences were conserved across a broad range of taxa (Table 3 and Fig. 5). In some congeneric species, no genetic variations were observed. These included *Epinephelus coioides* and *Epinephelus maculatus* (Fig. 6). Due to its high sequence conservation, distance-based inference may not be appropriate for 18S analysis as an approach to species assignment. The character-based method advocated by Sarkar et al. may be a suitable alternative [36]. In COI analysis based on the criterion of genetic distance, deep intraspecific divergences were observed in *Mene maculata* and *Terapon jarbua*, but unique type was characterized for each species based on the sequence analysis of 18S (Fig. 6). Exploring several gene regions for species markers and choosing a gene region and an appropriate measure for species identification can balance the potential for two types of errors: (1) mistreating individual variation for species level variation by using a relatively variable gene region; or (2) failing to identify true species differences, by using a conserved gene region to recover sufficient variation [37].

**Table 3:** Summary of K2P genetic p-distances (%) within different taxonomic levels.

	16S		Cyt *b*		18S	
Comparisons within	Mean	SE	Mean	SE	Mean	SE
species	0.27	0.05	0.78	0.11	0.16	0.02
genus	2.38	0.36	10.52	0.32	0.54	0.29
family	4.39	0.42	15.24	0.61	4.21	0.08
order	11.82	0.85	-		6.72	0.17

Values are calculated from DNA partial sequences of mitochondrial 16S rRNA (16S; n = 281), cytochrome *b* (cyt *b*; n = 124), and nuclear ribosomal 18S rRNA (16S; n = 276).

## Discussion

The ideal DNA barcoding should be robust, with conserved priming sites and reliable DNA amplifications and sequencing, and the DNA fragment sequenced should be nearly identical among individuals of the same species, but differentiative between species [38]. Therefore, we hope that DNA sequences exhibit high levels of conservation within the species and modest levels of genetic variability between different species. [39]. If the gene evolves too quickly, genetic variation would tends to be saturated at lower taxonomic groups. However, if it evolves too slowly, some closely allied species may not be differentiated. In other words, the high level of sequence conservation across a wide range of taxa can underestimate species diversity [40]. In this study, interspecific variations within the genera and families were close for 16S and 18S (Table 3 and Fig. 5). The presence of insertions and deletions in 16S and 18S can lead to errors in sequence alignment [41]. Compared to protein-coded COI and cyt*b*, the design of cyt*b* primers is surprisingly difficult given that COI is usually more conserved than cyt*b*. Based on the comprehensive analyses given above, the results show that the COI barcode region is a more suitable species marker across wide-range taxa.

Other DNA markers can provide assistance to species identification in cases where COI is lack of high resolving power. While DNA barcoding provides taxonomic identification for a given specimen, accuracy depends on whether there is an exact or nearly match to that species in the database. It is desirable that COI sequences representing each taxon in the reference database can cover the major part of the existing diversity, otherwise in the interrogation of BOLD, identification difficulties would arise when the unknown specimens come from a currently under-described part of biodiversity [42]. In case of low resolution from the COI gene alone, the combination of other molecular markers such as cyt*b*, 16S, and 18S can help solve this problem. For example, intraspecific variations of the COI gene in *Mene maculata* and *Terapon jarbua* were greater than the average of most intraspecific values, which imply possible overlaps with close related species if the sampling size is augmented continuously. In such cases, the sequence analysis of 18S sequences or other markers could help resolve this overlap should it occur.

Geographical structure, if ignored, can blur and distort species delineation [27]. Biological mechanisms, water dynamics, and even historical events may affect the deep genetic structure of marine populations [43]. Many explanations of genetic population structure on local and regional scales involve behaviors such as the adoption of pelagic early life stages and movement over broad geographic ranges. These factors are theoretically associated with gene flow. For marine fish, there is generally a lack of genetic differentiation within species on macrogeographic scales [44]–[47]. In this study, for many species, intraspecific genetic variations were near or equal to zero. However, some pairwise K2P distances of more than 1% were observed. Deep intraspecific genetic divergences were observed in species displaying restricted migratory behaviors or other biological mechanisms that would limit gene flow among individuals [48], [49]. *Siganus guttatus*, *Alepes djedaba*, *Scomber japonicus*, *Hyporhamphus limbatus*, *Terapon jarbua*, and *Pennahia anea* are coastal marine fish that reproduce in estuaries and bays and do not undertake large-scale migratory movements. The relevance of the reference DNA barcode database depends on the exhaustiveness of intra-taxon sampling, so the global participation and cooperation is indispensible for DNA barcoding projects.

**Figure 7:**
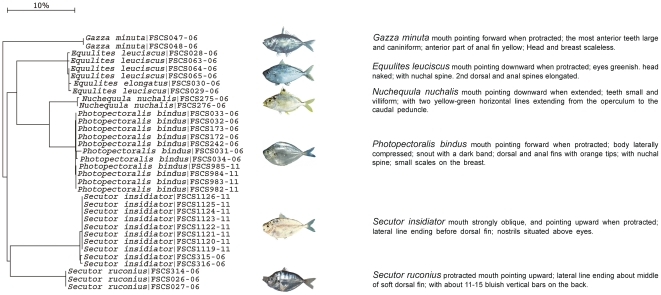
Neighbour joining tree (K2P) for Leiognathidae samples. Branches with specimen ID-number from BOLD and species name.

The combination of morphological and molecular characteristics can bridge the gap between morphological taxonomy and the DNA barcoding approach [37]. This idea has been embodied in the establishment of BOLD. DNA sequences in BOLD are derived from voucher specimens preserved in museums all around the world. Specimen data such as photo images and collection information are linked with each sequence. One can solve any problems concerning morphological identification by searching the relevant database or sending inquiries to confirm voucher specimens. The taxonomy of Leiognathidae species has changed drastically as a result of revisions carried out in recent years [50]. Several taxonomic designations of species used in the literature have been recognized as dubious identifications [51]. For example, *Nuchequula nuchalis* is misidentified as *Leiognathus nuchalis* [52] or *Leiognathus blochii* [53], and *Equulites leuciscus* is misidentified as *Leiognathus leuciscus* [53]. In this study, all genetic distances between *Nuchequula nuchalis* and *Equulites leuciscus* are over 15.80% [Fig. 7], and the value is greater than the average (13.55%) within genus. The big divergence among individuals of the two species supports the current taxonomy about Leiognathidae in which they should be classified into different genera [51]. In the genus *Pampus*, there are overlaps between intraspecific and interspecific genetic variations [Fig. 2]. Due to morphological similarities in *Pampus*, there is great confusion regarding the relative nomenclature [54]–[57]. *P. cinereus* is regarded by Parin and Piotrovsky as a synonym of *P. argenteus* based on morphological characeristics [57]. In the present study, *P. cinereus* and *P. argenteus* show small genetic variations and overlap in the NJ tree [Fig. 2], and our results support the idea that the nomenclature of *Pampus cinereus* may be removed as the synonym of *Pampus argenteus* in the FISHBASE. The results of DNA barcoding can also provide clues to the discovery of sample misidentification. One specimen of *Thrissa kammalensis*, which was collected off the west coast of the South China Sea, showed an average genetic divergence of 9.25% from other individuals of *Thrissa kammalensis*. However, its sequence was identical to those of *Thrissa setirostris*. The identification of this specimen merits suspicion because the value 9.25% greatly exceeds the average intraspecific genetic range. We checked the voucher specimen and found that this particular case had been misclassified. Species identification generally requires the collection of a large number of individuals, and occasional instances of misclassification are perhaps inevitable. Voucher specimens must be preserved in good condition for later collaborations and deposited for posterity in longstanding, legitimate collections dedicated to the storage of such materials [58]. Moreover, this example suggests that DNA barcoding can detect cases of morphological misclassification. The Fish Barcode of Life (FISH-BOL) campaign has the primary goal of gathering DNA barcode records for all of the world's fish. Standard reference DNA sequences amplified from expertly identified morphological voucher specimens can be used to better characterize and broadly identify species [10], [59].

One of the key concerns raised against DNA barcoding is that nuclear mitochondrial pseudogenes (numts) may misestimate the number of unique species [60], [61]. Actually, such problems were taken into account at the beginning of the DNA barcoding project [62]. Generally, submitted sequences are evaluated for suspicious numts in Barcode of Life Data Systems (BOLD, www.barcodinglife.org) if indels or stop codons are found. It seems possible that some numts may be of the expected length without any in-frame stop codons and therefore may not be readily distinguishable from the orthologous mtDNA [60]. Definite diagnosis is confirmed only by large numbers of sequence comparisons within and between species. We can set up a sub-database for numts in BOLD. After the abundant influx of the relevant data, the misidentification rate will dramatically decrease. In this study, over 1,000 specimens were amplified using universal primers, and only 4 numts were obtained, all of them in *Satyrichthys amiscus*. Orthologous mtDNAs were successfully amplified only by increasing the annealing temperature by 2°C. The number of mitochondrial genomes is greater than that of nuclear genomes, so conserved primers should preferentially amplify mtDNAs over numts. In special cases, several methods have been suggested as means of avoiding numt co-amplification. These include RT-PCR, long PCR, and mtDNA enrichment [63].

## Supporting Information

Table S1 Specimen data and GenBank accession numbers of these 1336 sequences (the specimen ID is the number which is given for each specimen in Barcode of Life Database (www.barcodinglife.org) containing all information from two projects, “Fishes from the South China Sea” (FSCS) and “Coral Fishes from the South China Sea” (FSCS).
(DOCX)

Table S2 Primers (5′-3′) utilized for PCR amplifications and sequencing in this study.
(DOCX)
