# Supplementary material for: Molecular Approach to the Identification of Fish in the South China Sea
Source: PLoS One. 2012 Feb 17;7(2):e30621. doi: 10.1371/journal.pone.0030621 (PMC3281855; doi:10.1371/journal.pone.0030621)
Supplement: Table S1 — Specimen data and GenBank accession numbers of these 1336 sequences (the specimen ID is the number which is given for each specimen in Barcode of Life Database (www.barcodinglife.org) containing all information from two projects, “Fishes from the South China Sea” (FSCS) and “Coral Fishes from the South China Sea” (FSCS). (DOCX) [file pone.0030621.s001.docx]

**Table S1** Specimen data and GenBank accession numbers of these 1336 sequences (the specimen ID is the number which is given for each specimen in Barcode of Life Database ([www.barcodinglife.org](http://www.barcodinglife.org)) containing all information from two projects, “Fishes from the South China Sea” (FSCS) and “Coral Fishes from the South China Sea” (FSCS).

| **Family** | **Genus** | **Species** | **Specimen ID** | **GenBank accession number** | **Museum number** | **Locality(Lat_ Lon)** |
| --- | --- | --- | --- | --- | --- | --- |
| Apogonidae | *Apogon* | *erythrinus* | FSCS193-06 | EF607315 | MBCSC:Fish:GD 9081041 | 20.454 110.717 |
|  | *Apogon* | *quadrifasciatus* | FSCS149-06 | EF607320 | MBCSC:Fish:GD 9085079 | 20.454 109.889 |
|  |  | *quadrifasciatus* | FSCS150-06 | EF607319 | MBCSC:Fish:GD 9085080 | 20.476 109.825 |
|  |  | *quadrifasciatus* | FSCS256-06 | EF607318 | MBCSC:Fish:GD 9083017 | 21.551 111.987 |
|  |  | *quadrifasciatus* | FSCS295-06 | EF607317 | MBCSC:Fish:GD 9087006 | 21.435 111.128 |
|  |  | *taeniatus* | FSCS191-06 | EF607322 | MBCSC:Fish:GD 9081039 | 20.808 110.6 |
|  |  | *taeniatus* | FSCS192-06 | EF607321 | MBCSC:Fish:GD 9081040 | 20.808 110.6 |
|  | *Nectamia* | *fusca* | FSCS190-06 | EF607316 | MBCSC:Fish:GD 9081038 | 20.702 110.564 |
| Ariidae | *Netuma* | *thalassina* | FSCS202-06 | EF607328 | MBCSC:Fish:GD 9081050 | 20.767 110.675 |
|  |  | *thalassina* | FSCS204-06 | EF607327 | MBCSC:Fish:GD 9081052 | 20.767 110.675 |
|  |  | *thalassina* | FSCS205-06 | EF607326 | MBCSC:Fish:GD 9081053 | 20.917 110.542 |
|  |  | *thalassina* | FSCS206-06 | EF607325 | MBCSC:Fish:GD 9081054 | 20.454 110.717 |
|  |  | *thalassina* | ^*^FSCS1027-11 | JN242656 | MBCSC:Fish:ZP1141251 | 21.333 111.977 |
|  |  | *thalassina* | FSCS1026-11 | JN242655 | MBCSC:Fish:LCG116208 | 23.388 117.449 |
|  |  | *thalassina* | FSCS1025-11 | JN242654 | MBCSC:Fish:ZP1141238 | 21.333 111.977 |
|  |  | *thalassina* | FSCS1024-11 | JN242653 | MBCSC:Fish:LCG116472 | 23.45 117.379 |
|  |  | *thalassina* | FSCS1023-11 | JN242652 | MBCSC:Fish:TCL116483 | 21.121 110.921 |
| Atherinidae | *Hypoatherina* | *valenciennei* | FSCS151-06 | EF607408 | MBCSC:Fish:GD 9085081 | 20.313 109.858 |
|  |  | *valenciennei* | FSCS152-06 | EF607407 | MBCSC:Fish:GD 9085082 | 20.313 109.858 |
|  |  | *valenciennei* | FSCS212-06 | EF607406 | MBCSC:Fish:GD 9081060 | 20.454 110.717 |
|  |  | *valenciennei* | FSCS213-06 | EF607405 | MBCSC:Fish:GD 9081061 | 20.454 110.717 |
| Belonidae | *Strongylura* | *leiura* | FSCS005-06 | EF607567 | MBCSC:Fish:GD 9085005 | 20.313 109.858 |
|  |  | *leiura* | FSCS006-06 | EF607566 | MBCSC:Fish:GD 9085006 | 20.313 109.858 |
|  |  | *strongylura* | FSCS153-06 | EF607571 | MBCSC:Fish:GD 9081001 | 20.808 110.6 |
|  |  | *strongylura* | FSCS154-06 | EF607570 | MBCSC:Fish:GD 9081002 | 20.917 110.542 |
|  |  | *strongylura* | FSCS187-06 | EF607569 | MBCSC:Fish:GD 9081035 | 20.808 110.6 |
|  |  | *sp.* | FSCS300-06 | EF607568 | MBCSC:Fish:GD 9087011 | 21.435 111.128 |
| Bothidae | *Arnoglossus* | *polyspilus* | FSCS116-06 | EF607331 | MBCSC:Fish:GD 9085046 | 20.476 109.825 |
|  |  | *polyspilus* | FSCS117-06 | EF607330 | MBCSC:Fish:GD 9085047 | 20.416 109.841 |
|  |  | *polyspilus* | FSCS118-06 | EF607329 | MBCSC:Fish:GD 9085048 | 20.476 109.825 |
| Bramidae | *Brama* | *japonica* | FSCS865-11 | JN242505 | MBCSC:Fish:ZP11412101 | 21.333 111.977 |
|  |  | *japonica* | FSCS864-11 | JN242504 | MBCSC:Fish:XS1169103 | 17.311 113.276 |
|  |  | *japonica* | FSCS863-11 | JN242503 | MBCSC:Fish:XS1169102 | 17.311 113.276 |
|  |  | *japonica* | FSCS862-11 | JN242502 | MBCSC:Fish:LCG116203 | 23.388 117.449 |
|  |  | *japonica* | FSCS861-11 | JN242501 | MBCSC:Fish:TCL116462 | 21.121 110.921 |
| Callionymidae | *Bathycallionymus* | *kaianus* | FSCS310-06 | EF607336 | MBCSC:Fish:GD 9084003 | 22.678 114.549 |
|  | *Repomucenus* | *richardsonii* | FSCS136-06 | EF607502 | MBCSC:Fish:GD 9085066 | 20.454 109.889 |
|  |  | *richardsonii* | FSCS137-06 | EF607501 | MBCSC:Fish:GD 9085067 | 20.476 109.825 |
|  |  | *richardsonii* | FSCS138-06 | EF607500 | MBCSC:Fish:GD 9085068 | 20.476 109.825 |
|  |  | *richardsonii* | FSCS139-06 | EF607499 | MBCSC:Fish:GD 9085069 | 20.313 109.858 |
| Carangidae | *Alepes* | *djedaba* | FSCS088-06 | EF607312 | MBCSC:Fish:GD 9086063 | 20.313 109.858 |
|  |  | *djedaba* | FSCS166-06 | EF607311 | MBCSC:Fish:GD 9081014 | 20.767 110.675 |
|  |  | *djedaba* | FSCS167-06 | EF607310 | MBCSC:Fish:GD 9081015 | 20.808 110.6 |
|  |  | *djedaba* | FSCS260-06 | EF607309 | MBCSC:Fish:GD 9082003 | 21.089 110.452 |
|  |  | *djedaba* | FSCS261-06 | EF607308 | MBCSC:Fish:GD 9082004 | 21.047 110.665 |
|  |  | *djedaba* | FSCS292-06 | EF607307 | MBCSC:Fish:GD 9087003 | 21.42 111.169 |
|  | *Atule* | *mate* | FSCS257-06 | EF607335 | MBCSC:Fish:GD 9083018 | 21.551 111.987 |
|  |  | *mate* | FSCS333-07 | EU595067 | MBCSC:Fish:ZC I07003 | 21.091 112.553 |
|  |  | *mate* | FSCS334-07 | EU595066 | MBCSC:Fish:ZC I07004 | 19.366 113.422 |
|  |  | *mate* | FSCS335-07 | EU595065 | MBCSC:Fish:ZC I07005 | 21.007 111.223 |
|  |  | *mate* | FSCS336-07 | EU595064 | MBCSC:Fish:ZC I07006 | 21.007 111.223 |
|  |  | *mate* | FSCS337-07 | EU595063 | MBCSC:Fish:ZC I07007 | 20.516 110.981 |
|  |  | *mate* | FSCS338-07 | EU595062 | MBCSC:Fish:ZC I07008 | 20.516 110.981 |
|  |  | *mate* | FSCS550-07 | EU595061 | MBCSC:Fish:ZC I07216 | 20.045 111.484 |
|  |  | *mate* | FSCS551-07 | EU595060 | MBCSC:Fish:ZC I07217 | 18.045 111.484 |
|  |  | *mate* | FSCS552-07 | EU595059 | MBCSC:Fish:ZC I07218 | 21.091 112.553 |
|  |  | *mate* | FSCS553-07 | EU595058 | MBCSC:Fish:ZC I07219 | 17.35 112.553 |
|  |  | *mate* | FSCS754-08 | FJ237969 | MBCSC:Fish:ZC I07329 | 19.685 112.767 |
|  |  | *mate* | FSCS757-08 | FJ237968 | MBCSC:Fish:ZC I07332 | 19.685 112.767 |
|  |  | *mate* | FSCS764-08 | FJ237967 | MBCSC:Fish:ZC I07339 | 19.685 112.767 |
|  | *Carangoides* | *malabaricus* | FSCS012-06 | EF607341 | MBCSC:Fish:GD 9085012 | 20.313 109.858 |
|  |  | *malabaricus* | FSCS013-06 | EF607340 | MBCSC:Fish:GD 9085013 | 20.454 109.889 |
|  |  | *chrysophrys* | FSCS096-06 | EF607339 | MBCSC:Fish:GD 9085026 | 20.476 109.825 |
|  |  | *chrysophrys* | FSCS097-06 | EF607338 | MBCSC:Fish:GD 9085027 | 20.416 109.841 |
|  |  | *chrysophrys* | CFCS028-08 | FJ237673 | MBCSC:Fish:HN SY08344 | 17.073 109.736 |
|  |  | *chrysophrys* | CFCS029-08 | FJ237672 | MBCSC:Fish:HN SY08345 | 16.677 112.898 |
|  |  | *chrysophrys* | CFCS227-08 | FJ237671 | MBCSC:Fish:HN SY08543 | 17.073 109.736 |
|  |  | *chrysophrys* | CFCS228-08 | FJ237670 | MBCSC:Fish:HN SY08544 | 17.073 109.736 |
|  |  | *chrysophrys* | CFCS229-08 | FJ237669 | MBCSC:Fish:HN SY08545 | 17.073 109.736 |
|  |  | *chrysophrys* | CFCS230-08 | FJ237668 | MBCSC:Fish:HN SY08546 | 17.073 109.736 |
|  |  | *praeustus* | FSCS572-07 | EU595071 | MBCSC:Fish:ZC I07242 | 20.497 113.568 |
|  |  | *praeustus* | FSCS573-07 | EU595070 | MBCSC:Fish:ZC I07243 | 20.497 113.568 |
|  |  | *praeustus* | FSCS574-07 | EU595069 | MBCSC:Fish:ZC I07244 | 21.074 111.557 |
|  |  | *praeustus* | FSCS575-07 | EU595068 | MBCSC:Fish:ZC I07245 | 21.074 111.557 |
|  |  | *praeustus* | FSCS796-08 | FJ237972 | MBCSC:Fish:ZC I07371 | 20.55 111.2 |
|  |  | *praeustus* | FSCS799-08 | FJ237971 | MBCSC:Fish:ZC I07374 | 19.685 112.767 |
|  |  | *praeustus* | FSCS800-08 | FJ237970 | MBCSC:Fish:ZC I07375 | 20.875 111.21 |
|  | *Decapterus* | *akaadsi* | FSCS194-06 | EF607334 | MBCSC:Fish:GD 9081042 | 20.767 110.675 |
|  |  | *akaadsi* | FSCS195-06 | EF607333 | MBCSC:Fish:GD 9081043 | 20.454 110.717 |
|  |  | *akaadsi* | FSCS296-06 | EF607332 | MBCSC:Fish:GD 9087007 | 21.435 111.128 |
|  | *Scomberoides* | *tol* | FSCS063-06 | EF607531 | MBCSC:Fish:GD 9086038 | 20.454 109.889 |
|  |  | *tol* | FSCS064-06 | EF607530 | MBCSC:Fish:GD 9086039 | 20.313 109.858 |
|  |  | *tol* | FSCS065-06 | EF607529 | MBCSC:Fish:GD 9086040 | 20.313 109.858 |
|  |  | *tol* | FSCS066-06 | EF607528 | MBCSC:Fish:GD 9086041 | 20.313 109.858 |
|  |  | *tol* | FSCS067-06 | EF607527 | MBCSC:Fish:GD 9086042 | 20.313 109.858 |
|  | *Selaroides* | *leptolepis* | FSCS083-06 | EF607548 | MBCSC:Fish:GD 9086058 | 20.313 109.858 |
|  |  | *leptolepis* | FSCS084-06 | EF607547 | MBCSC:Fish:GD 9086059 | 20.313 109.858 |
|  |  | *leptolepis* | FSCS085-06 | EF607546 | MBCSC:Fish:GD 9086060 | 20.313 109.858 |
|  |  | *leptolepis* | FSCS086-06 | EF607545 | MBCSC:Fish:GD 9086061 | 20.476 109.825 |
|  |  | *leptolepis* | FSCS087-06 | EF607550 | MBCSC:Fish:GD 9086062 | 20.476 109.825 |
|  |  | *leptolepis* | FSCS179-06 | EF607549 | MBCSC:Fish:GD 9081027 | 20.808 110.6 |
|  | *Seriola* | *dumerili* | CFCS024-08 | FJ237927 | MBCSC:Fish:HN SY08340 | 16.583 112.756 |
|  |  | *dumerili* | CFCS042-08 | FJ237926 | MBCSC:Fish:HN SY08358 | 18.216 109.447 |
|  |  | *dumerili* | CFCS258-08 | FJ237925 | MBCSC:Fish:HN SY08574 | 18.234 109.455 |
|  |  | *dumerili* | CFCS259-08 | FJ237924 | MBCSC:Fish:HN SY08575 | 18.234 109.455 |
|  |  | *dumerili* | CFCS260-08 | FJ237923 | MBCSC:Fish:HN SY08576 | 18.234 109.455 |
|  |  | *dumerili* | CFCS261-08 | FJ237922 | MBCSC:Fish:HN SY08577 | 18.234 109.455 |
|  |  | *dumerili* | CFCS262-08 | FJ237921 | MBCSC:Fish:HN SY08578 | 18.234 109.455 |
|  | *Trachinotus* | *ovatus* | FSCS482-07 | EU595326 | MBCSC:Fish:ZC I07141 | 19.366 113.422 |
|  |  | *ovatus* | FSCS483-07 | EU595325 | MBCSC:Fish:ZC I07142 | 19.685 112.767 |
|  |  | *ovatus* | FSCS792-08 | FJ238047 | MBCSC:Fish:ZC I07367 | 20.875 111.21 |
|  |  | *ovatus* | FSCS793-08 | FJ238046 | MBCSC:Fish:ZC I07368 | 20.65 111.487 |
|  |  | *ovatus* | FSCS794-08 | FJ238045 | MBCSC:Fish:ZC I07369 | 20.55 111.2 |
|  |  | *ovatus* | FSCS795-08 | FJ238044 | MBCSC:Fish:ZC I07370 | 20.55 111.2 |
|  |  | *ovatus* | FSCS797-08 | FJ238043 | MBCSC:Fish:ZC I07372 | 20.875 111.21 |
|  |  | *ovatus* | FSCS1148-11 | JN242715 | MBCSC:Fish:XS116963 | 17.311 113.276 |
|  |  | *ovatus* | FSCS1147-11 | JN242717 | MBCSC:Fish:BH1122623 | 21.232 109.377 |
|  |  | *ovatus* | FSCS1146-11 | JN242716 | MBCSC:Fish:BH1122622 | 21.232 109.377 |
|  |  | *ovatus* | FSCS1145-11 | JN242714 | MBCSC:Fish:XS116979 | 17.311 113.276 |
|  |  | *ovatus* | FSCS1144-11 | JN242713 | MBCSC:Fish:XS116978 | 17.311 113.276 |
|  |  | *ovatus* | FSCS1143-11 | JN242712 | MBCSC:Fish:ZP1141211 | 21.333 111.977 |
|  |  | *ovatus* | FSCS1142-11 | JN242711 | MBCSC:Fish:ZP1141210 | 21.333 111.977 |
| Carcharhinidae | *Scoliodon* | *sp.* | FSCS238-06 | EF607378 | MBCSC:Fish:GD 9081086 | 20.702 110.564 |
|  |  | *sp.* | FSCS568-07 | EU595127 | MBCSC:Fish:ZC I07237 | 19.685 112.767 |
|  |  | *sp.* | FSCS569-07 | EU595126 | MBCSC:Fish:ZC I07238 | 19.685 112.767 |
|  |  | *sp.* | FSCS570-07 | EU595125 | MBCSC:Fish:ZC I07239 | 19.685 112.767 |
| Centrolophidae | *Psenopsis* | *anomala* | FSCS391-07 | EU595253 | MBCSC:Fish:ZC I07052 | 19.366 113.422 |
|  |  | *anomala* | FSCS392-07 | EU595252 | MBCSC:Fish:ZC I07053 | 19.685 112.767 |
|  |  | *anomala* | FSCS393-07 | EU595251 | MBCSC:Fish:ZC I07054 | 19.685 112.767 |
|  |  | *anomala* | FSCS395-07 | EU595250 | MBCSC:Fish:ZC I07056 | 19.685 112.767 |
| Chaetodontidae | *Chaetodon* | *auriga* | CFCS071-08 | FJ237683 | MBCSC:Fish:HN SY08387 | 18.234 109.455 |
|  |  | *auriga* | CFCS072-08 | FJ237682 | MBCSC:Fish:HN SY08388 | 18.234 109.455 |
|  |  | *auriga* | CFCS319-08 | FJ237681 | MBCSC:Fish:HN SY08635 | 18.234 109.455 |
|  |  | *auripes* | CFCS076-08 | FJ237687 | MBCSC:Fish:HN SY08392 | 18.216 109.447 |
|  |  | *auripes* | CFCS082-08 | FJ237686 | MBCSC:Fish:HN SY08398 | 18.234 109.455 |
|  |  | *auripes* | CFCS328-08 | FJ237685 | MBCSC:Fish:HN SY08644 | 18.234 109.455 |
|  |  | *auripes* | CFCS329-08 | FJ237684 | MBCSC:Fish:HN SY08645 | 18.234 109.455 |
|  |  | *wiebeli* | CFCS073-08 | FJ237698 | MBCSC:Fish:HN SY08389 | 18.216 109.447 |
|  |  | *wiebeli* | CFCS074-08 | FJ237697 | MBCSC:Fish:HN SY08390 | 18.216 109.447 |
|  |  | *wiebeli* | CFCS075-08 | FJ237696 | MBCSC:Fish:HN SY08391 | 18.216 109.447 |
|  |  | *wiebeli* | CFCS320-08 | FJ237695 | MBCSC:Fish:HN SY08636 | 18.234 109.455 |
|  |  | *wiebeli* | CFCS321-08 | FJ237694 | MBCSC:Fish:HN SY08637 | 18.234 109.455 |
|  |  | *wiebeli* | CFCS322-08 | FJ237693 | MBCSC:Fish:HN SY08638 | 18.234 109.455 |
|  |  | *wiebeli* | CFCS323-08 | FJ237692 | MBCSC:Fish:HN SY08639 | 18.234 109.455 |
|  |  | *wiebeli* | CFCS324-08 | FJ237691 | MBCSC:Fish:HN SY08640 | 18.234 109.455 |
|  |  | *wiebeli* | CFCS325-08 | FJ237690 | MBCSC:Fish:HN SY08641 | 18.234 109.455 |
|  |  | *wiebeli* | CFCS326-08 | FJ237689 | MBCSC:Fish:HN SY08642 | 18.234 109.455 |
|  |  | *wiebeli* | CFCS327-08 | FJ237688 | MBCSC:Fish:HN SY08643 | 18.234 109.455 |
| Chanidae | *Chanos* | *chanos* | FSCS868-11 | JN242679 | MBCSC:Fish:TCL116421 | 21.121 110.921 |
|  |  | *chanos* | FSCS867-11 | JN242678 | MBCSC:Fish:BH1122605 | 21.232 109.377 |
|  |  | *chanos* | FSCS866-11 | JN242677 | MBCSC:Fish:TCL116459 | 21.121 110.921 |
| Chirocentridae | *Chirocentrus* | *nudus* | FSCS056-06 | EF607345 | MBCSC:Fish:GD 9086031 | 20.313 109.858 |
|  |  | *nudus* | FSCS057-06 | EF607344 | MBCSC:Fish:GD 9086032 | 20.313 109.858 |
|  |  | *nudus* | FSCS058-06 | EF607343 | MBCSC:Fish:GD 9086033 | 20.416 109.841 |
|  |  | *nudus* | FSCS175-06 | EF607346 | MBCSC:Fish:GD 9081023 | 20.917 110.542 |
|  |  | *nudus* | FSCS878-11 | JN242564 | MBCSC:Fish:ZP1141201 | 21.333 111.977 |
|  |  | *nudus* | FSCS877-11 | JN242563 | MBCSC:Fish:BH1122601 | 21.232 109.377 |
|  |  | *nudus* | FSCS876-11 | JN242562 | MBCSC:Fish:LCG116205 | 23.388 117.449 |
|  |  | *nudus* | FSCS875-11 | JN242561 | MBCSC:Fish:BH1122696 | 21.232 109.377 |
|  |  | *dorab* | FSCS176-06 | EF607342 | MBCSC:Fish:GD 9081024 | 20.808 110.6 |
| Clupeidae | *Amblygaster* | *clupeoides* | FSCS286-06 | EF607313 | MBCSC:Fish:GD 9082029 | 21.047 110.665 |
|  | *Dussumieria* | *elopsoides* | FSCS035-06 | EF607362 | MBCSC:Fish:GD 9086010 | 20.454 109.889 |
|  |  | *elopsoides* | FSCS036-06 | EF607361 | MBCSC:Fish:GD 9086011 | 20.416 109.841 |
|  |  | *elopsoides* | FSCS262-06 | EF607363 | MBCSC:Fish:GD 9082005 | 21.047 110.665 |
|  | *Escualosa* | *thoracata* | FSCS020-06 | EF607375 | MBCSC:Fish:GD 9085020 | 20.416 109.841 |
|  |  | *thoracata* | FSCS021-06 | EF607374 | MBCSC:Fish:GD 9085021 | 20.313 109.858 |
|  |  | *thoracata* | FSCS022-06 | EF607373 | MBCSC:Fish:GD 9085022 | 20.313 109.858 |
|  |  | *thoracata* | FSCS024-06 | EF607372 | MBCSC:Fish:GD 9085024 | 20.313 109.858 |
|  |  | *thoracata* | FSCS258-06 | EF607377 | MBCSC:Fish:GD 9082001 | 21.094 110.529 |
|  |  | *thoracata* | FSCS259-06 | EF607376 | MBCSC:Fish:GD 9082002 | 21.094 110.529 |
|  | *Konosirus* | *punctatus* | FSCS339-07 | EU595157 | MBCSC:Fish:ZC I07009 | 19.366 113.422 |
|  |  | *punctatus* | FSCS340-07 | EU595156 | MBCSC:Fish:ZC I07010 | 21.074 111.557 |
|  |  | *punctatus* | FSCS341-07 | EU595155 | MBCSC:Fish:ZC I07011 | 21.091 112.553 |
|  |  | *punctatus* | FSCS342-07 | EU595154 | MBCSC:Fish:ZC I07012 | 21.238 111.667 |
|  |  | *punctatus* | FSCS758-08 | FJ237996 | MBCSC:Fish:ZC I07333 | 19.685 112.767 |
|  |  | *punctatus* | FSCS759-08 | FJ237995 | MBCSC:Fish:ZC I07334 | 19.685 112.767 |
|  | *Nematalosa* | *japonica* | FSCS287-06 | EF607513 | MBCSC:Fish:GD 9082030 | 21.047 110.665 |
|  | *Sardinella* | *jussieu* | FSCS271-06 | EF607507 | MBCSC:Fish:GD 9082014 | 21.047 110.665 |
|  |  | *jussieu* | FSCS272-06 | EF607506 | MBCSC:Fish:GD 9082015 | 21.089 110.452 |
|  |  | *lemuru* | FSCS327-06 | EF607505 | MBCSC:Fish:GD 9088015 | 23.296 116.784 |
|  |  | *lemuru* | FSCS328-06 | EF607504 | MBCSC:Fish:GD 9088016 | 23.256 116.833 |
|  |  | *lemuru* | FSCS329-06 | EF607503 | MBCSC:Fish:GD 9088017 | 23.256 116.833 |
|  |  | *lemuru* | FSCS413-07 | EU595256 | MBCSC:Fish:ZC I07072 | 21.074 111.557 |
|  |  | *lemuru* | FSCS414-07 | EU595255 | MBCSC:Fish:ZC I07073 | 21.074 111.557 |
|  |  | *lemuru* | FSCS417-07 | EU595254 | MBCSC:Fish:ZC I07076 | 20.516 110.981 |
|  |  | *melanura* | FSCS168-06 | EF607512 | MBCSC:Fish:GD 9081016 | 20.702 110.564 |
|  |  | *melanura* | FSCS169-06 | EF607511 | MBCSC:Fish:GD 9081017 | 20.767 110.675 |
|  |  | *melanura* | FSCS235-06 | EF607510 | MBCSC:Fish:GD 9081083 | 20.702 110.564 |
|  |  | *melanura* | FSCS268-06 | EF607509 | MBCSC:Fish:GD 9082011 | 21.047 110.665 |
|  |  | *melanura* | FSCS269-06 | EF607508 | MBCSC:Fish:GD 9082012 | 21.094 110.529 |
|  |  | *melanura* | FSCS534-07 | EU595266 | MBCSC:Fish:ZC I07203 | 20.516 110.981 |
|  |  | *melanura* | FSCS535-07 | EU595265 | MBCSC:Fish:ZC I07204 | 20.516 110.981 |
|  |  | *melanura* | FSCS536-07 | EU595264 | MBCSC:Fish:ZC I07205 | 20.516 110.981 |
|  |  | *melanura* | FSCS537-07 | EU595263 | MBCSC:Fish:ZC I07206 | 20.045 111.484 |
|  |  | *melanura* | FSCS538-07 | EU595262 | MBCSC:Fish:ZC I07207 | 20.045 111.484 |
|  |  | *melanura* | FSCS539-07 | EU595261 | MBCSC:Fish:ZC I07208 | 20.045 111.484 |
|  |  | *melanura* | FSCS540-07 | EU595260 | MBCSC:Fish:ZC I07209 | 20.045 111.484 |
|  |  | *melanura* | FSCS541-07 | EU595259 | MBCSC:Fish:ZC I07210 | 20.045 111.484 |
|  |  | *melanura* | FSCS542-07 | EU595258 | MBCSC:Fish:ZC I07211 | 20.045 111.484 |
|  |  | *melanura* | FSCS543-07 | EU595257 | MBCSC:Fish:ZC I07212 | 20.045 111.484 |
|  |  | *melanura* | FSCS750-08 | FJ238023 | MBCSC:Fish:ZC I07325 | 20.65 111.487 |
|  |  | *melanura* | FSCS751-08 | FJ238022 | MBCSC:Fish:ZC I07326 | 20.65 111.487 |
|  |  | *melanura* | FSCS752-08 | FJ238021 | MBCSC:Fish:ZC I07327 | 20.65 111.487 |
| Congridae | *Conger* | *japonicus* | FSCS233-06 | EF607456 | MBCSC:Fish:GD 9081081 | 20.702 110.564 |
|  |  | *japonicus* | FSCS320-06 | EF607455 | MBCSC:Fish:GD 9088008 | 23.256 116.833 |
|  |  | *japonicus* | FSCS578-07 | EU595082 | MBCSC:Fish:ZC I07248 | 19.685 112.767 |
|  |  | *japonicus* | FSCS801-08 | FJ237976 | MBCSC:Fish:ZC I07376 | 20.55 111.2 |
|  |  | *japonicus* | FSCS802-08 | FJ237975 | MBCSC:Fish:ZC I07377 | 20.55 111.2 |
|  |  | *myriaster* | FSCS579-07 | EU595083 | MBCSC:Fish:ZC I07249 | 20.045 111.484 |
|  | *Gnathophis* | *nystromi* | FSCS429-07 | EU595142 | MBCSC:Fish:ZC I07089 | 21.476 111.998 |
|  |  | *nystromi* | FSCS430-07 | EU595141 | MBCSC:Fish:ZC I07090 | 20.383 109.117 |
|  |  | *nystromi* | FSCS431-07 | EU595140 | MBCSC:Fish:ZC I07091 | 20.283 111.776 |
|  |  | *nystromi* | FSCS432-07 | EU595139 | MBCSC:Fish:ZC I07093 | 21.475 111.883 |
|  |  | *nystromi* | FSCS433-07 | EU595138 | MBCSC:Fish:ZC I07094 | 20.335 112.568 |
|  |  | *nystromi* | FSCS434-07 | EU595137 | MBCSC:Fish:ZC I07095 | 20.045 111.484 |
|  |  | *nystromi* | FSCS435-07 | EU595136 | MBCSC:Fish:ZC I07097 | 20.045 111.484 |
|  |  | *nystromi* | FSCS436-07 | EU595135 | MBCSC:Fish:ZC I07098 | 20.045 111.484 |
|  |  | *nystromi* | FSCS838-08 | FJ237989 | MBCSC:Fish:ZC I07413 | 20.55 111.2 |
|  |  | *nystromi* | FSCS839-08 | FJ237988 | MBCSC:Fish:ZC I07414 | 20.65 111.487 |
|  | *Uroconger* | *lepturus* | FSCS437-07 | EU595337 | MBCSC:Fish:ZC I07099 | 21.007 111.223 |
|  |  | *lepturus* | FSCS438-07 | EU595336 | MBCSC:Fish:ZC I07100 | 21.007 111.223 |
|  |  | *lepturus* | FSCS439-07 | EU595335 | MBCSC:Fish:ZC I07101 | 21.007 111.223 |
| Coryphaenidae | *Coryphaena* | *hippurus* | CFCS001-08 | FJ237709 | MBCSC:Fish:HN SY08317 | 17.073 109.736 |
|  |  | *hippurus* | CFCS124-08 | FJ237708 | MBCSC:Fish:HN SY08440 | 17.073 109.736 |
|  |  | *hippurus* | CFCS125-08 | FJ237707 | MBCSC:Fish:HN SY08441 | 17.073 109.736 |
| Cynoglossidae | *Cynoglossus* | *bilineatus* | FSCS114-06 | EF607350 | MBCSC:Fish:GD 9085044 | 20.454 109.889 |
|  |  | *bilineatus* | FSCS115-06 | EF607349 | MBCSC:Fish:GD 9085045 | 20.476 109.825 |
|  |  | *bilineatus* | FSCS125-06 | EF607348 | MBCSC:Fish:GD 9085055 | 20.416 109.841 |
|  |  | *itinus* | FSCS372-07 | EU595088 | MBCSC:Fish:ZC I07035 | 19.366 113.422 |
|  |  | *itinus* | FSCS373-07 | EU595087 | MBCSC:Fish:ZC I07036 | 19.685 112.767 |
|  |  | *itinus* | FSCS374-07 | EU595086 | MBCSC:Fish:ZC I07088 | 20.65 109.567 |
|  |  | *puncticeps* | FSCS110-06 | EF607352 | MBCSC:Fish:GD 9085040 | 20.476 109.825 |
|  |  | *puncticeps* | FSCS112-06 | EF607351 | MBCSC:Fish:GD 9085042 | 20.476 109.825 |
|  |  | *puncticeps* | FSCS497-07 | EU595089 | MBCSC:Fish:ZC I07156 | 21.007 111.223 |
|  |  | *semilaevis* | FSCS899-11 | JN242744 | MBCSC:Fish:ZP1141203 | 21.333 111.977 |
|  |  | *semilaevis* | FSCS898-11 | JN242743 | MBCSC:Fish:ZP1141202 | 21.333 111.977 |
|  |  | *semilaevis* | FSCS896-11 | JN242742 | MBCSC:Fish:TCL116479 | 21.121 110.921 |
|  |  | *sp.* | FSCS111-06 | EF607353 | MBCSC:Fish:GD 9085041 | 20.476 109.825 |
|  |  | *sp.* | FSCS498-07 | EU595091 | MBCSC:Fish:ZC I07157 | 21.007 111.223 |
|  |  | *sp.* | FSCS499-07 | EU595090 | MBCSC:Fish:ZC I07158 | 21.091 112.553 |
|  | *Paraplagusia* | *japonica* | FSCS113-06 | EF607480 | MBCSC:Fish:GD 9085043 | 20.454 109.889 |
| Dactylopteridae | *Dactyloptena* | *orientalis* | CFCS044-08 | FJ237717 | MBCSC:Fish:HN SY08360 | 18.216 109.447 |
|  |  | *orientalis* | CFCS045-08 | FJ237716 | MBCSC:Fish:HN SY08361 | 16.035 114.134 |
|  |  | *orientalis* | CFCS046-08 | FJ237715 | MBCSC:Fish:HN SY08362 | 17.788 109.024 |
|  |  | *orientalis* | CFCS267-08 | FJ237714 | MBCSC:Fish:HN SY08583 | 17.788 109.024 |
|  |  | *orientalis* | CFCS268-08 | FJ237713 | MBCSC:Fish:HN SY08584 | 17.788 109.024 |
|  |  | *orientalis* | CFCS269-08 | FJ237712 | MBCSC:Fish:HN SY08585 | 17.788 109.024 |
|  |  | *orientalis* | CFCS270-08 | FJ237711 | MBCSC:Fish:HN SY08586 | 17.788 109.024 |
|  |  | *orientalis* | CFCS271-08 | FJ237710 | MBCSC:Fish:HN SY08587 | 17.788 109.024 |
| Dasyatididae | *Dasyatis* | *bennetti* | FSCS521-07 | EU595098 | MBCSC:Fish:ZC I07190 | 21.007 111.223 |
|  |  | *bennetti* | FSCS522-07 | EU595097 | MBCSC:Fish:ZC I07191 | 21.007 111.223 |
|  |  | *bennetti* | FSCS523-07 | EU595096 | MBCSC:Fish:ZC I07192 | 19.366 113.422 |
|  |  | *bennetti* | FSCS524-07 | EU595095 | MBCSC:Fish:ZC I07193 | 19.366 113.422 |
|  |  | *bennetti* | FSCS525-07 | EU595094 | MBCSC:Fish:ZC I07194 | 19.366 113.422 |
|  |  | *bennetti* | FSCS526-07 | EU595093 | MBCSC:Fish:ZC I07195 | 19.366 113.422 |
|  |  | *bennetti* | FSCS527-07 | EU595092 | MBCSC:Fish:ZC I07196 | 16.345 111.422 |
|  |  | *bennetti* | FSCS831-08 | FJ237982 | MBCSC:Fish:ZC I07406 | 19.685 112.767 |
|  |  | *bennetti* | FSCS832-08 | FJ237981 | MBCSC:Fish:ZC I07407 | 20.65 111.487 |
|  |  | *bennetti* | FSCS833-08 | FJ237980 | MBCSC:Fish:ZC I07408 | 20.65 111.487 |
|  |  | *bennetti* | FSCS834-08 | FJ237979 | MBCSC:Fish:ZC I07409 | 19.685 112.767 |
|  |  | *bennetti* | FSCS835-08 | FJ237978 | MBCSC:Fish:ZC I07410 | 20.65 111.487 |
|  |  | *bennetti* | FSCS836-08 | FJ237977 | MBCSC:Fish:ZC I07411 | 19.685 112.767 |
|  |  | *bennettii* | FSCS906-11 | JN242685 | MBCSC:Fish:BH1122653 | 21.232 109.377 |
|  |  | *bennettii* | FSCS905-11 | JN242684 | MBCSC:Fish:BH1122652 | 21.232 109.377 |
|  |  | *bennettii* | FSCS904-11 | JN242683 | MBCSC:Fish:ZH1151823 | 21.125 113.42 |
|  |  | *bennettii* | FSCS903-11 | JN242682 | MBCSC:Fish:XS116974 | 17.311 113.276 |
|  |  | *bennettii* | FSCS902-11 | JN242681 | MBCSC:Fish:LCG116206 | 23.388 117.449 |
|  |  | *bennettii* | FSCS901-11 | JN242680 | MBCSC:Fish:XS1169127 | 17.311 113.276 |
|  |  | *zugei* | FSCS008-06 | EF607354 | MBCSC:Fish:GD 9085008 | 20.313 109.858 |
|  |  | *zugei* | FSCS528-07 | EU595099 | MBCSC:Fish:ZC I07197 | 19.366 113.422 |
| Drepaneidae | *Drepane* | *punctata* | FSCS239-06 | EF607360 | MBCSC:Fish:GD 9081087 | 20.454 110.717 |
|  |  | *punctata* | FSCS472-07 | EU595102 | MBCSC:Fish:ZC I07133 | 21.007 111.223 |
|  |  | *punctata* | FSCS473-07 | EU595101 | MBCSC:Fish:ZC I07134 | 21.091 112.553 |
|  |  | *punctata* | FSCS474-07 | EU595100 | MBCSC:Fish:ZC I07181 | 21.091 112.553 |
|  |  | *punctata* | FSCS787-08 | FJ237984 | MBCSC:Fish:ZC I07362 | 20.65 111.487 |
|  |  | *punctata* | FSCS788-08 | FJ237983 | MBCSC:Fish:ZC I07363 | 20.65 111.487 |
|  |  | *punctata* | FSCS909-11 | JN242500 | MBCSC:Fish:ZH1151891 | 21.125 113.42 |
|  |  | *punctata* | FSCS908-11 | JN242499 | MBCSC:Fish:ZP1141248 | 21.333 111.977 |
|  |  | *punctata* | FSCS907-11 | JN242498 | MBCSC:Fish:XS116971 | 17.311 113.276 |
| Elopidae | *Elops* | *hawaiensis* | FSCS059-06 | EF607366 | MBCSC:Fish:GD 9086034 | 20.313 109.858 |
|  |  | *hawaiensis* | FSCS060-06 | EF607365 | MBCSC:Fish:GD 9086035 | 20.313 109.858 |
|  |  | *hawaiensis* | FSCS061-06 | EF607364 | MBCSC:Fish:GD 9086036 | 20.313 109.858 |
|  |  | *hawaiensis* | FSCS062-06 | EF607367 | MBCSC:Fish:GD 9086037 | 20.313 109.858 |
|  |  | *hawaiensis* | FSCS396-07 | EU595109 | MBCSC:Fish:ZC I07059 | 20.497 113.568 |
| Engraulidae | *Anchoviella* | *sp.* | FSCS265-06 | EF607314 | MBCSC:Fish:GD 9082008 | 21.089 110.452 |
|  | *Engraulis* | *japonicus* | FSCS263-06 | EF607370 | MBCSC:Fish:GD 9082006 | 21.094 110.529 |
|  |  | *japonicus* | FSCS266-06 | EF607369 | MBCSC:Fish:GD 9082009 | 21.047 110.665 |
|  |  | *japonicus* | FSCS267-06 | EF607368 | MBCSC:Fish:GD 9082010 | 21.047 110.665 |
|  | *Stolephorus* | *indicus* | FSCS446-07 | EU595317 | MBCSC:Fish:ZC I07107 | 20.045 111.484 |
|  |  | *indicus* | FSCS447-07 | EU595316 | MBCSC:Fish:ZC I07108 | 20.045 111.484 |
|  |  | *indicus* | FSCS448-07 | EU595315 | MBCSC:Fish:ZC I07109 | 19.685 112.767 |
|  |  | *indicus* | FSCS449-07 | EU595314 | MBCSC:Fish:ZC I07110 | 19.685 112.767 |
|  |  | *indicus* | FSCS450-07 | EU595313 | MBCSC:Fish:ZC I07111 | 19.685 112.767 |
|  |  | *indicus* | FSCS451-07 | EU595312 | MBCSC:Fish:ZC I07112 | 21.007 111.223 |
|  |  | *indicus* | FSCS452-07 | EU595311 | MBCSC:Fish:ZC I07113 | 21.007 111.223 |
|  |  | *indicus* | FSCS453-07 | EU595310 | MBCSC:Fish:ZC I07114 | 21.007 111.223 |
|  |  | *indicus* | FSCS454-07 | EU595320 | MBCSC:Fish:ZC I07115 | 21.007 111.223 |
|  |  | *indicus* | FSCS455-07 | EU595319 | MBCSC:Fish:ZC I07116 | 21.007 111.223 |
|  |  | *indicus* | FSCS456-07 | EU595318 | MBCSC:Fish:ZC I07118 | 21.007 111.223 |
|  |  | *indicus* | FSCS755-08 | FJ238041 | MBCSC:Fish:ZC I07330 | 19.685 112.767 |
|  |  | *indicus* | FSCS756-08 | FJ238040 | MBCSC:Fish:ZC I07331 | 19.685 112.767 |
|  |  | *indicus* | FSCS762-08 | FJ238039 | MBCSC:Fish:ZC I07337 | 20.65 111.487 |
|  | *Thryssa* | *hamiltonii* | FSCS016-06 | EF607589 | MBCSC:Fish:GD 9085016 | 20.313 109.858 |
|  |  | *hamiltonii* | FSCS019-06 | EF607588 | MBCSC:Fish:GD 9085019 | 20.313 109.858 |
|  |  | *kammalensis* | FSCS015-06 | EF607591 | MBCSC:Fish:GD 9085015 | 20.454 109.889 |
|  |  | *kammalensis* | FSCS017-06 | EF607590 | MBCSC:Fish:GD 9085017 | 20.416 109.841 |
|  |  | *kammalensis* | FSCS159-06 | EF607596 | MBCSC:Fish:GD 9081007 | 20.767 110.675 |
|  |  | *kammalensis* | FSCS160-06 | EF607595 | MBCSC:Fish:GD 9081008 | 20.767 110.675 |
|  |  | *kammalensis* | FSCS161-06 | EF607594 | MBCSC:Fish:GD 9081009 | 20.767 110.675 |
|  |  | *kammalensis* | FSCS240-06 | EF607593 | MBCSC:Fish:GD 9083001 | 20.55 111.815 |
|  |  | *kammalensis* | FSCS241-06 | EF607592 | MBCSC:Fish:GD 9083002 | 21.658 111.815 |
|  |  | *setirostris* | FSCS014-06 | EF607598 | MBCSC:Fish:GD 9085014 | 20.313 109.858 |
|  |  | *setirostris* | FSCS018-06 | EF607597 | MBCSC:Fish:GD 9085018 | 20.454 109.889 |
|  |  | *setirostris* | FSCS158-06 | EF607599 | MBCSC:Fish:GD 9081006 | 20.917 110.542 |
| Etmopteridae | *Etmopterus* | *lucifer* | FSCS162-06 | EF607381 | MBCSC:Fish:GD 9081010 | 20.917 110.542 |
|  |  | *lucifer* | FSCS236-06 | EF607380 | MBCSC:Fish:GD 9081084 | 20.454 110.717 |
|  |  | *lucifer* | FSCS237-06 | EF607379 | MBCSC:Fish:GD 9081085 | 20.454 110.717 |
|  |  | *lucifer* | FSCS571-07 | EU595124 | MBCSC:Fish:ZC I07241 | 19.685 112.767 |
| Exocoetidae | *Hirundichthys* | *rondeletii* | FSCS079-06 | EF607404 | MBCSC:Fish:GD 9086054 | 20.313 109.858 |
|  |  | *rondeletii* | FSCS080-06 | EF607403 | MBCSC:Fish:GD 9086055 | 20.313 109.858 |
| Fistulariidae | *Fistularia* | *commersonii* | FSCS214-06 | EF607384 | MBCSC:Fish:GD 9081062 | 20.454 110.717 |
|  |  | *commersonii* | FSCS215-06 | EF607383 | MBCSC:Fish:GD 9081063 | 20.917 110.542 |
| Gerreidae | *Gerres* | *erythrourus* | FSCS222-06 | EF607385 | MBCSC:Fish:GD 9081070 | 20.454 110.717 |
|  |  | *erythrourus* | FSCS254-06 | EF607387 | MBCSC:Fish:GD 9083015 | 21.551 111.987 |
|  |  | *erythrourus* | FSCS312-06 | EF607386 | MBCSC:Fish:GD 9084005 | 22.749 115.211 |
|  |  | *filamentosus* | FSCS940-11 | JN242560 | MBCSC:Fish:LCG1162101 | 23.388 117.449 |
|  |  | *filamentosus* | FSCS939-11 | JN242559 | MBCSC:Fish:ZH1151898 | 21.125 113.42 |
|  |  | *filamentosus* | FSCS938-11 | JN242558 | MBCSC:Fish:TCL116428 | 21.121 110.921 |
|  |  | *filamentosus* | FSCS937-11 | JN242557 | MBCSC:Fish:ZH11518136 | 21.125 113.42 |
|  |  | *limbatus* | FSCS231-06 | EF607388 | MBCSC:Fish:GD 9081079 | 20.702 110.564 |
|  |  | *limbatus* | FSCS232-06 | EF607392 | MBCSC:Fish:GD 9081080 | 20.454 110.717 |
|  |  | *limbatus* | FSCS250-06 | EF607391 | MBCSC:Fish:GD 9083011 | 21.658 111.815 |
|  |  | *limbatus* | FSCS282-06 | EF607390 | MBCSC:Fish:GD 9082025 | 21.089 110.452 |
|  |  | *limbatus* | FSCS283-06 | EF607389 | MBCSC:Fish:GD 9082026 | 21.047 110.665 |
|  |  | *oblongus* | FSCS223-06 | EF607393 | MBCSC:Fish:GD 9081071 | 20.767 110.675 |
| Gobiidae | *Acentrogobius* | *caninus* | FSCS037-06 | EF607303 | MBCSC:Fish:GD 9086012 | 20.313 109.858 |
|  |  | *caninus* | FSCS038-06 | EF607302 | MBCSC:Fish:GD 9086013 | 20.416 109.841 |
|  |  | *caninus* | FSCS039-06 | EF607301 | MBCSC:Fish:GD 9086014 | 20.416 109.841 |
|  |  | *caninus* | FSCS170-06 | EF607306 | MBCSC:Fish:GD 9081018 | 20.917 110.542 |
|  |  | *caninus* | FSCS273-06 | EF607305 | MBCSC:Fish:GD 9082016 | 21.047 110.665 |
|  |  | *caninus* | FSCS274-06 | EF607304 | MBCSC:Fish:GD 9082017 | 21.089 110.452 |
|  | *Favonigobius* | *gymnauchen* | FSCS044-06 | EF607382 | MBCSC:Fish:GD 9086019 | 20.416 109.841 |
|  | *Glossogobius* | *aureus* | FSCS082-06 | EF607394 | MBCSC:Fish:GD 9086057 | 20.454 109.889 |
|  | *Oxyurichthys* | *tentacularis* | FSCS1046-11 | JN242590 | MBCSC:Fish:XS116917 | 17.311 113.276 |
|  |  | *tentacularis* | FSCS1045-11 | JN242589 | MBCSC:Fish:XS116914 | 17.311 113.276 |
|  |  | *tentacularis* | FSCS1044-11 | JN242588 | MBCSC:Fish:ZP1141230 | 21.333 111.977 |
|  |  | *tentacularis* | FSCS1043-11 | JN242587 | MBCSC:Fish:LCG1132506 | 23.666 118.05 |
|  |  | *tentacularis* | FSCS1042-11 | JN242586 | MBCSC:Fish:XS116921 | 17.311 113.276 |
|  |  | *tentacularis* | FSCS1041-11 | JN242585 | MBCSC:Fish:BH1122646 | 21.232 109.377 |
|  | *Scartelaos* | *histophorus* | FSCS458-07 | EU595282 | MBCSC:Fish:ZC I07119 | 21.091 112.553 |
|  |  | *histophorus* | FSCS459-07 | EU595281 | MBCSC:Fish:ZC I07120 | 21.091 112.553 |
|  |  | *histophorus* | FSCS460-07 | EU595280 | MBCSC:Fish:ZC I07121 | 21.091 112.553 |
|  |  | *histophorus* | FSCS461-07 | EU595279 | MBCSC:Fish:ZC I07122 | 21.091 112.553 |
|  |  | *histophorus* | FSCS462-07 | EU595278 | MBCSC:Fish:ZC I07123 | 21.091 112.553 |
|  |  | *histophorus* | FSCS463-07 | EU595277 | MBCSC:Fish:ZC I07124 | 21.091 112.553 |
|  |  | *histophorus* | FSCS464-07 | EU595276 | MBCSC:Fish:ZC I07125 | 21.007 111.223 |
|  |  | *histophorus* | FSCS465-07 | EU595275 | MBCSC:Fish:ZC I07126 | 21.007 111.223 |
|  |  | *histophorus* | FSCS466-07 | EU595274 | MBCSC:Fish:ZC I07127 | 21.007 111.223 |
|  |  | *histophorus* | FSCS467-07 | EU595273 | MBCSC:Fish:ZC I07128 | 21.007 111.223 |
|  |  | *histophorus* | FSCS468-07 | EU595272 | MBCSC:Fish:ZC I07129 | 21.007 111.223 |
|  |  | *histophorus* | FSCS469-07 | EU595271 | MBCSC:Fish:ZC I07130 | 21.007 111.223 |
|  |  | *histophorus* | FSCS770-08 | FJ238032 | MBCSC:Fish:ZC I07345 | 20.875 111.21 |
|  |  | *histophorus* | FSCS771-08 | FJ238031 | MBCSC:Fish:ZC I07346 | 20.65 111.487 |
|  |  | *histophorus* | FSCS772-08 | FJ238030 | MBCSC:Fish:ZC I07347 | 19.685 112.767 |
|  |  | *histophorus* | FSCS776-08 | FJ238029 | MBCSC:Fish:ZC I07351 | 20.65 111.487 |
|  |  | *histophorus* | FSCS777-08 | FJ238028 | MBCSC:Fish:ZC I07352 | 20.875 111.21 |
|  |  | *histophorus* | FSCS778-08 | FJ238027 | MBCSC:Fish:ZC I07353 | 20.65 111.487 |
|  |  | *histophorus* | FSCS779-08 | FJ238026 | MBCSC:Fish:ZC I07354 | 20.875 111.21 |
|  |  | *histophorus* | FSCS781-08 | FJ238025 | MBCSC:Fish:ZC I07356 | 20.875 111.21 |
| Haemulidae | *Diagramma* | *pictum* | FSCS477-07 | EU595232 | MBCSC:Fish:ZC I07137 | 19.366 113.422 |
|  |  | *pictum* | FSCS478-07 | EU595231 | MBCSC:Fish:ZC I07176 | 19.366 113.422 |
|  | *Parapristipoma* | *trilineatum* | FSCS216-06 | EF607482 | MBCSC:Fish:GD 9081064 | 20.454 110.717 |
|  |  | *trilineatum* | FSCS217-06 | EF607481 | MBCSC:Fish:GD 9081065 | 20.454 110.717 |
|  | *Plectorhinchus* | *cinctus* | FSCS442-07 | EU595230 | MBCSC:Fish:ZC I07096 | 21.007 111.223 |
|  | *Pomadasys* | *hasta* | FSCS049-06 | EF607494 | MBCSC:Fish:GD 9086024 | 20.416 109.841 |
|  |  | *hasta* | FSCS050-06 | EF607493 | MBCSC:Fish:GD 9086025 | 20.454 109.889 |
|  |  | *hasta* | FSCS051-06 | EF607492 | MBCSC:Fish:GD 9086026 | 20.416 109.841 |
|  |  | *hasta* | FSCS052-06 | EF607491 | MBCSC:Fish:GD 9086027 | 20.313 109.858 |
|  |  | *hasta* | FSCS174-06 | EF607495 | MBCSC:Fish:GD 9081022 | 20.702 110.564 |
|  |  | *maculatus* | FSCS470-07 | EU595244 | MBCSC:Fish:ZC I07131 | 20.045 111.484 |
|  |  | *maculatus* | FSCS471-07 | EU595243 | MBCSC:Fish:ZC I07132 | 20.045 111.484 |
|  |  | *maculatus* | FSCS784-08 | FJ238017 | MBCSC:Fish:ZC I07359 | 20.65 111.487 |
|  |  | *maculatus* | CFCS025-08 | FJ237890 | MBCSC:Fish:HN SY08341 | 17.073 109.736 |
|  |  | *maculatus* | CFCS220-08 | FJ237889 | MBCSC:Fish:HN SY08536 | 17.073 109.736 |
|  |  | *maculatus* | FSCS785-08 | FJ238016 | MBCSC:Fish:ZC I07360 | 20.875 111.21 |
| Hapalogenyidae | *Hapalogenys* | *nigripinnis* | FSCS944-11 | JN242704 | MBCSC:Fish:BH1122615 | 21.232 109.377 |
|  |  | *nigripinnis* | FSCS943-11 | JN242703 | MBCSC:Fish:ZP1141204 | 21.333 111.977 |
|  |  | *nigripinnis* | FSCS942-11 | JN242702 | MBCSC:Fish:TCL116410 | 21.121 110.921 |
|  |  | *nigripinnis* | FSCS941-11 | JN242701 | MBCSC:Fish:ZH1151804 | 21.125 113.42 |
| Hemiramphidae | *Hyporhamphus* | *dussumieri* | FSCS076-06 | EF607401 | MBCSC:Fish:GD 9086051 | 20.476 109.825 |
|  |  | *dussumieri* | FSCS077-06 | EF607400 | MBCSC:Fish:GD 9086052 | 20.454 109.889 |
|  |  | *limbatus* | FSCS177-06 | EF607402 | MBCSC:Fish:GD 9081025 | 20.767 110.675 |
|  |  | *limbatus* | FSCS592-07 | EU595152 | MBCSC:Fish:ZC I07262 | 21.074 111.557 |
|  |  | *limbatus* | FSCS593-07 | EU595151 | MBCSC:Fish:ZC I07263 | 20.337 111.856 |
|  |  | *limbatus* | FSCS594-07 | EU595150 | MBCSC:Fish:ZC I07264 | 20.871 111.335 |
|  |  | *limbatus* | FSCS595-07 | EU595149 | MBCSC:Fish:ZC I07265 | 20.664 111.986 |
|  |  | *limbatus* | FSCS596-07 | EU595148 | MBCSC:Fish:ZC I07266 | 19.767 113.334 |
|  |  | *limbatus* | FSCS597-07 | EU595147 | MBCSC:Fish:ZC I07267 | 21.333 112.158 |
|  |  | *limbatus* | FSCS814-08 | FJ237993 | MBCSC:Fish:ZC I07389 | 20.55 111.2 |
|  |  | *quoyi* | FSCS457-07 | EU595153 | MBCSC:Fish:ZC I07117 | 21.007 111.223 |
|  |  | *quoyi* | FSCS763-08 | FJ237994 | MBCSC:Fish:ZC I07338 | 19.685 112.767 |
| Hemiscylliidae | *Chiloscyllium* | *plagiosum* | FSCS331-07 | EU595075 | MBCSC:Fish:ZC I07103 | 8.667 112.533 |
|  |  | *plagiosum* | FSCS443-07 | EU595074 | MBCSC:Fish:ZC I07104 | 9.58 113.422 |
|  |  | *plagiosum* | FSCS444-07 | EU595073 | MBCSC:Fish:ZC I07105 | 9.867 115.811 |
|  |  | *plagiosum* | FSCS753-08 | FJ237974 | MBCSC:Fish:ZC I07328 | 19.685 112.767 |
|  |  | *plagiosum* | FSCS874-11 | JN242710 | MBCSC:Fish:TCL116463 | 21.121 110.921 |
|  |  | *plagiosum* | FSCS873-11 | JN242709 | MBCSC:Fish:XS116997 | 17.311 113.276 |
|  |  | *plagiosum* | FSCS872-11 | JN242708 | MBCSC:Fish:XS116993 | 17.311 113.276 |
|  |  | *plagiosum* | FSCS871-11 | JN242707 | MBCSC:Fish:LCG116412 | 23.45 117.379 |
|  |  | *plagiosum* | FSCS870-11 | JN242706 | MBCSC:Fish:ZH1151882 | 21.125 113.42 |
|  |  | *plagiosum* | FSCS869-11 | JN242705 | MBCSC:Fish:ZP1141247 | 21.333 111.977 |
| Holocentridae | *Ostichthys* | *japonicus* | CFCS030-08 | FJ237861 | MBCSC:Fish:HN SY08346 | 16.583 112.756 |
|  |  | *japonicus* | CFCS031-08 | FJ237860 | MBCSC:Fish:HN SY08347 | 16.035 114.134 |
|  |  | *japonicus* | CFCS032-08 | FJ237859 | MBCSC:Fish:HN SY08348 | 16.035 114.134 |
|  |  | *japonicus* | CFCS231-08 | FJ237858 | MBCSC:Fish:HN SY08547 | 17.073 109.736 |
|  |  | *japonicus* | CFCS232-08 | FJ237857 | MBCSC:Fish:HN SY08548 | 17.073 109.736 |
|  |  | *japonicus* | CFCS233-08 | FJ237856 | MBCSC:Fish:HN SY08549 | 18.237 108.161 |
|  |  | *japonicus* | CFCS234-08 | FJ237855 | MBCSC:Fish:HN SY08550 | 17.073 109.736 |
|  |  | *japonicus* | CFCS235-08 | FJ237854 | MBCSC:Fish:HN SY08551 | 18.237 108.161 |
|  |  | *japonicus* | CFCS236-08 | FJ237853 | MBCSC:Fish:HN SY08552 | 16.583 112.756 |
|  |  | *japonicus* | CFCS237-08 | FJ237852 | MBCSC:Fish:HN SY08553 | 16.867 111.36 |
|  |  | *japonicus* | CFCS238-08 | FJ237851 | MBCSC:Fish:HN SY08554 | 16.583 112.756 |
|  |  | *japonicus* | CFCS239-08 | FJ237850 | MBCSC:Fish:HN SY08555 | 16.583 112.756 |
|  |  | *japonicus* | CFCS240-08 | FJ237849 | MBCSC:Fish:HN SY08556 | 16.583 112.756 |
|  |  | *japonicus* | FSCS1040-11 | JN242603 | MBCSC:Fish:ZH1151883 | 21.125 113.42 |
|  |  | *japonicus* | FSCS1039-11 | JN242602 | MBCSC:Fish:XS116907 | 17.311 113.276 |
|  |  | *japonicus* | FSCS1038-11 | JN242601 | MBCSC:Fish:ZP1141225 | 21.333 111.977 |
|  |  | *japonicus* | FSCS1037-11 | JN242600 | MBCSC:Fish:ZP1141224 | 21.333 111.977 |
|  |  | *japonicus* | FSCS1036-11 | JN242599 | MBCSC:Fish:BH1122645 | 21.232 109.377 |
|  |  | *japonicus* | FSCS1035-11 | JN242598 | MBCSC:Fish:BH1122644 | 21.232 109.377 |
|  |  | *japonicus* | FSCS1034-11 | JN242597 | MBCSC:Fish:LCG116318 | 23.125 117.876 |
|  |  | *japonicus* | FSCS1033-11 | JN242596 | MBCSC:Fish:XS116924 | 17.311 113.276 |
|  | *Sargocentron* | *rubrum* | CFCS242-08 | FJ237913 | MBCSC:Fish:HN SY08558 | 16.583 112.756 |
|  |  | *rubrum* | CFCS245-08 | FJ237912 | MBCSC:Fish:HN SY08561 | 18.234 109.455 |
| Labridae | *Bodianus* | *oxycephalus* | CFCS020-08 | FJ237632 | MBCSC:Fish:HN SY08336 | 16.378 112.452 |
|  |  | *oxycephalus* | CFCS199-08 | FJ237631 | MBCSC:Fish:HN SY08515 | 18.237 108.161 |
|  |  | *oxycephalus* | CFCS200-08 | FJ237630 | MBCSC:Fish:HN SY08516 | 18.237 108.161 |
|  |  | *oxycephalus* | CFCS201-08 | FJ237629 | MBCSC:Fish:HN SY08517 | 18.237 108.161 |
|  |  | *oxycephalus* | CFCS202-08 | FJ237628 | MBCSC:Fish:HN SY08518 | 17.073 109.736 |
|  |  | *oxycephalus* | CFCS203-08 | FJ237627 | MBCSC:Fish:HN SY08519 | 17.073 109.736 |
|  |  | *oxycephalus* | CFCS204-08 | FJ237626 | MBCSC:Fish:HN SY08520 | 17.073 109.736 |
|  |  | *tanyokidus* | CFCS021-08 | FJ237633 | MBCSC:Fish:HN SY08337 | 16.677 112.898 |
|  | *Cheilinus* | *undulatus* | FSCS616-07 | EU595072 | MBCSC:Fish:ZC I07288 | 16.643 113.336 |
|  |  | *undulatus* | FSCS829-08 | FJ237973 | MBCSC:Fish:ZC I07404 | 20.65 111.487 |
|  | *Choerodon* | *azurio* | CFCS023-08 | FJ237706 | MBCSC:Fish:HN SY08339 | 17.073 109.736 |
|  |  | *azurio* | CFCS208-08 | FJ237705 | MBCSC:Fish:HN SY08524 | 17.073 109.736 |
|  |  | *azurio* | CFCS209-08 | FJ237704 | MBCSC:Fish:HN SY08525 | 17.073 109.736 |
|  |  | *azurio* | CFCS210-08 | FJ237703 | MBCSC:Fish:HN SY08526 | 17.073 109.736 |
|  |  | *azurio* | CFCS211-08 | FJ237702 | MBCSC:Fish:HN SY08527 | 17.073 109.736 |
|  |  | *azurio* | CFCS214-08 | FJ237701 | MBCSC:Fish:HN SY08530 | 17.073 109.736 |
|  |  | *azurio* | CFCS215-08 | FJ237700 | MBCSC:Fish:HN SY08531 | 17.073 109.736 |
|  |  | *azurio* | FSCS882-11 | JN242484 | MBCSC:Fish:ZP1141234 | 21.333 111.977 |
|  |  | *azurio* | FSCS881-11 | JN242483 | MBCSC:Fish:ZP1141233 | 21.333 111.977 |
|  |  | *azurio* | FSCS880-11 | JN242482 | MBCSC:Fish:XS116995 | 17.311 113.276 |
|  |  | *azurio* | FSCS879-11 | JN242481 | MBCSC:Fish:ZP1141291 | 21.333 111.977 |
|  | *Hemigymnus* | *melapterus* | CFCS112-08 | FJ237787 | MBCSC:Fish:HN SY08428 | 16.035 114.134 |
|  |  | *melapterus* | CFCS113-08 | FJ237786 | MBCSC:Fish:HN SY08429 | 16.378 112.452 |
|  |  | *melapterus* | CFCS186-08 | FJ237785 | MBCSC:Fish:HN SY08502 | 17.073 109.736 |
|  |  | *melapterus* | CFCS187-08 | FJ237784 | MBCSC:Fish:HN SY08503 | 17.073 109.736 |
|  |  | *melapterus* | CFCS189-08 | FJ237783 | MBCSC:Fish:HN SY08505 | 17.073 109.736 |
|  |  | *melapterus* | CFCS191-08 | FJ237782 | MBCSC:Fish:HN SY08507 | 18.237 108.161 |
|  |  | *melapterus* | CFCS192-08 | FJ237781 | MBCSC:Fish:HN SY08508 | 17.073 109.736 |
|  |  | *melapterus* | CFCS193-08 | FJ237780 | MBCSC:Fish:HN SY08509 | 17.073 109.736 |
|  |  | *melapterus* | CFCS194-08 | FJ237779 | MBCSC:Fish:HN SY08510 | 18.237 108.161 |
|  |  | *melapterus* | CFCS195-08 | FJ237778 | MBCSC:Fish:HN SY08511 | 17.073 109.736 |
|  |  | *melapterus* | CFCS196-08 | FJ237777 | MBCSC:Fish:HN SY08512 | 17.073 109.736 |
|  | *Thalassoma* | *lunare* | FSCS198-06 | EF607582 | MBCSC:Fish:GD 9081046 | 20.454 110.717 |
|  | *Xyrichtys* | *verrens* | FSCS1156-11 | JN242508 | MBCSC:Fish:ZH1151836 | 21.125 113.42 |
|  |  | *verrens* | FSCS1155-11 | JN242507 | MBCSC:Fish:BH1122607 | 21.232 109.377 |
|  |  | *verrens* | FSCS1154-11 | JN242506 | MBCSC:Fish:XS116954 | 17.311 113.276 |
| Lactariidae | *Lactarius* | *lactarius* | FSCS010-06 | EF607417 | MBCSC:Fish:GD 9085010 | 20.476 109.825 |
|  |  | *lactarius* | FSCS011-06 | EF607416 | MBCSC:Fish:GD 9085011 | 20.313 109.858 |
|  |  | *lactarius* | FSCS157-06 | EF607418 | MBCSC:Fish:GD 9081005 | 20.808 110.6 |
| Lateolabracidae | *Lateolabrax* | *maculatus* | FSCS981-11 | JN242650 | MBCSC:Fish:XS116941 | 17.311 113.276 |
|  |  | *maculatus* | FSCS980-11 | JN242651 | MBCSC:Fish:LCG1132508 | 23.666 118.05 |
|  |  | *maculatus* | FSCS979-11 | JN242649 | MBCSC:Fish:ZP11412142 | 21.333 111.977 |
|  |  | *maculatus* | FSCS978-11 | JN242648 | MBCSC:Fish:BH1122640 | 21.232 109.377 |
|  |  | *maculatus* | FSCS977-11 | JN242647 | MBCSC:Fish:ZP11412139 | 21.333 111.977 |
| Latidae | *Lates* | *calcarifer* | FSCS397-07 | EU595186 | MBCSC:Fish:ZC I07060 | 20.045 111.484 |
|  |  | *calcarifer* | FSCS399-07 | EU595185 | MBCSC:Fish:ZC I07167 | 20.516 110.981 |
|  |  | *calcarifer* | FSCS400-07 | EU595184 | MBCSC:Fish:ZC I07168 | 19.685 112.767 |
|  |  | *calcarifer* | FSCS401-07 | EU595183 | MBCSC:Fish:ZC I07169 | 21.007 111.223 |
|  |  | *calcarifer* | FSCS402-07 | EU595182 | MBCSC:Fish:ZC I07170 | 20.497 113.568 |
|  |  | *calcarifer* | FSCS404-07 | EU595181 | MBCSC:Fish:ZC I07172 | 21.007 111.223 |
|  |  | *calcarifer* | FSCS405-07 | EU595180 | MBCSC:Fish:ZC I07173 | 19.685 112.767 |
|  |  | *calcarifer* | FSCS406-07 | EU595179 | MBCSC:Fish:ZC I07174 | 19.366 113.422 |
|  |  | *calcarifer* | FSCS407-07 | EU595178 | MBCSC:Fish:ZC I07175 | 19.685 112.767 |
|  |  | *calcarifer* | FSCS816-08 | FJ238005 | MBCSC:Fish:ZC I07391 | 19.685 112.767 |
|  |  | *calcarifer* | FSCS817-08 | FJ238004 | MBCSC:Fish:ZC I07392 | 19.685 112.767 |
|  |  | *calcarifer* | FSCS818-08 | FJ238003 | MBCSC:Fish:ZC I07393 | 20.55 111.2 |
|  |  | *calcarifer* | FSCS819-08 | FJ238002 | MBCSC:Fish:ZC I07394 | 20.55 111.2 |
|  |  | *calcarifer* | FSCS822-08 | FJ238001 | MBCSC:Fish:ZC I07397 | 19.685 112.767 |
|  |  | *calcarifer* | FSCS823-08 | FJ238000 | MBCSC:Fish:ZC I07398 | 20.65 111.487 |
|  |  | *calcarifer* | FSCS824-08 | FJ237999 | MBCSC:Fish:ZC I07399 | 19.685 112.767 |
|  |  | *sp.* | FSCS398-07 | EU595188 | MBCSC:Fish:ZC I07166 | 17.738 113.183 |
|  |  | *sp.* | FSCS403-07 | EU595187 | MBCSC:Fish:ZC I07171 | 17.738 113.183 |
| Leiognathidae | *Equulites* | *leuciscus* | FSCS028-06 | EF607431 | MBCSC:Fish:GD 9086003 | 20.313 109.858 |
|  |  |  |  |  |  |  |
|  |  | *leuciscus* | FSCS029-06 | EF607430 | MBCSC:Fish:GD 9086004 | 20.416 109.841 |
|  |  |  |  |  |  |  |
|  |  | *leuciscus* | FSCS030-06 | EF607429 | MBCSC:Fish:GD 9086005 | 20.454 109.889 |
|  |  | *leuciscus* | FSCS163-06 | EF607434 | MBCSC:Fish:GD 9081011 | 20.808 110.6 |
|  |  | *leuciscus* | FSCS164-06 | EF607433 | MBCSC:Fish:GD 9081012 | 20.917 110.542 |
|  |  | *leuciscus* | FSCS165-06 | EF607432 | MBCSC:Fish:GD 9081013 | 20.454 110.717 |
|  | *Gazza* | *minuta* | FSCS047-06 | EF607428 | MBCSC:Fish:GD 9086022 | 20.416 109.841 |
|  |  | *minuta* | FSCS048-06 | EF607427 | MBCSC:Fish:GD 9086023 | 20.313 109.858 |
|  | *Photopectoralis* | *bindus* | FSCS031-06 | EF607423 | MBCSC:Fish:GD 9086006 | 20.416 109.841 |
|  |  | *bindus* | FSCS032-06 | EF607422 | MBCSC:Fish:GD 9086007 | 20.476 109.825 |
|  |  | *bindus* | FSCS033-06 | EF607421 | MBCSC:Fish:GD 9086008 | 20.454 109.889 |
|  |  | *bindus* | FSCS034-06 | EF607420 | MBCSC:Fish:GD 9086009 | 20.416 109.841 |
|  |  | *bindus* | FSCS172-06 | EF607426 | MBCSC:Fish:GD 9081020 | 20.702 110.564 |
|  |  | *bindus* | FSCS173-06 | EF607425 | MBCSC:Fish:GD 9081021 | 20.917 110.542 |
|  |  | *bindus* | FSCS242-06 | EF607424 | MBCSC:Fish:GD 9083003 | 21.573 111.727 |
|  |  | *bindus* | FSCS985-11 | JN242721 | MBCSC:Fish:ZP1141237 | 21.333 111.977 |
|  |  | *bindus* | FSCS984-11 | JN242720 | MBCSC:Fish:LCG1141823 | 23.5 117.666 |
|  |  | *bindus* | FSCS983-11 | JN242719 | MBCSC:Fish:LCG1141822 | 23.5 117.666 |
|  |  | *bindus* | FSCS982-11 | JN242718 | MBCSC:Fish:ZH1151828 | 21.125 113.42 |
|  | *Nuchequula* | *nuchalis* | FSCS275-06 | EF607436 | MBCSC:Fish:GD 9082018 | 21.047 110.665 |
|  |  | *nuchalis* | FSCS276-06 | EF607435 | MBCSC:Fish:GD 9082019 | 21.089 110.452 |
|  | *Secutor* | *insidiator* | FSCS315-06 | EF607541 | MBCSC:Fish:GD 9088003 | 23.36 116.84 |
|  |  | *insidiator* | FSCS316-06 | EF607540 | MBCSC:Fish:GD 9088004 | 23.256 116.833 |
|  |  | *insidiator* | FSCS1126-11 | JN242556 | MBCSC:Fish:XS116943 | 17.311 113.276 |
|  |  | *insidiator* | FSCS1125-11 | JN242555 | MBCSC:Fish:TCL116416 | 21.121 110.921 |
|  |  | *insidiator* | FSCS1124-11 | JN242554 | MBCSC:Fish:TCL116415 | 21.121 110.921 |
|  |  | *insidiator* | FSCS1123-11 | JN242553 | MBCSC:Fish:XS116939 | 17.311 113.276 |
|  |  | *insidiator* | FSCS1122-11 | JN242552 | MBCSC:Fish:ZH1151805 | 21.125 113.42 |
|  |  | *insidiator* | FSCS1121-11 | JN242551 | MBCSC:Fish:ZP1141213 | 21.333 111.977 |
|  |  | *insidiator* | FSCS1120-11 | JN242550 | MBCSC:Fish:ZP1141212 | 21.333 111.977 |
|  |  | *insidiator* | FSCS1119-11 | JN242549 | MBCSC:Fish:BH1122624 | 21.232 109.377 |
|  |  | *ruconius* | FSCS026-06 | EF607543 | MBCSC:Fish:GD 9086001 | 20.313 109.858 |
|  |  | *ruconius* | FSCS027-06 | EF607542 | MBCSC:Fish:GD 9086002 | 20.416 109.841 |
|  |  | *ruconius* | FSCS314-06 | EF607544 | MBCSC:Fish:GD 9088002 | 23.36 116.84 |
| Lethrinidae | *Lethrinus* | *lentjan* | FSCS280-06 | EF607440 | MBCSC:Fish:GD 9082023 | 21.089 110.452 |
|  |  | *lentjan* | FSCS281-06 | EF607439 | MBCSC:Fish:GD 9082024 | 21.047 110.665 |
|  |  | *lentjan* | CFCS119-08 | FJ237802 | MBCSC:Fish:HN SY08435 | 18.216 109.447 |
|  |  | *lentjan* | CFCS120-08 | FJ237801 | MBCSC:Fish:HN SY08436 | 18.216 109.447 |
|  |  | *lentjan* | CFCS121-08 | FJ237800 | MBCSC:Fish:HN SY08437 | 18.216 109.447 |
|  |  | *lentjan* | CFCS218-08 | FJ237799 | MBCSC:Fish:HN SY08534 | 18.234 109.455 |
|  |  | *lentjan* | CFCS219-08 | FJ237798 | MBCSC:Fish:HN SY08535 | 18.234 109.455 |
|  |  | *haematopterus* | CFCS026-08 | FJ237797 | MBCSC:Fish:HN SY08342 | 16.583 112.756 |
|  |  | *haematopterus* | CFCS027-08 | FJ237796 | MBCSC:Fish:HN SY08343 | 16.583 112.756 |
|  |  | *haematopterus* | CFCS221-08 | FJ237795 | MBCSC:Fish:HN SY08537 | 18.237 108.161 |
|  |  | *haematopterus* | CFCS222-08 | FJ237794 | MBCSC:Fish:HN SY08538 | 17.073 109.736 |
|  |  | *haematopterus* | CFCS223-08 | FJ237793 | MBCSC:Fish:HN SY08539 | 17.073 109.736 |
|  |  | *haematopterus* | CFCS224-08 | FJ237792 | MBCSC:Fish:HN SY08540 | 17.073 109.736 |
|  |  | *haematopterus* | CFCS225-08 | FJ237791 | MBCSC:Fish:HN SY08541 | 18.237 108.161 |
|  |  | *haematopterus* | CFCS226-08 | FJ237790 | MBCSC:Fish:HN SY08542 | 17.073 109.736 |
| Lobotidae | *Lobotes* | *surinamensis* | CFCS068-08 | FJ237808 | MBCSC:Fish:HN SY08384 | 17.788 109.024 |
|  |  | *surinamensis* | CFCS188-08 | FJ237807 | MBCSC:Fish:HN SY08504 | 18.237 108.161 |
|  |  | *surinamensis* | CFCS310-08 | FJ237806 | MBCSC:Fish:HN SY08626 | 17.788 109.024 |
|  |  | *surinamensis* | CFCS312-08 | FJ237805 | MBCSC:Fish:HN SY08628 | 17.788 109.024 |
|  |  | *surinamensis* | CFCS313-08 | FJ237804 | MBCSC:Fish:HN SY08629 | 17.788 109.024 |
|  |  | *surinamensis* | CFCS314-08 | FJ237803 | MBCSC:Fish:HN SY08630 | 17.788 109.024 |
|  |  | *surinamensis* | CFCS068-08 | FJ237808 | MBCSC:Fish:HN SY08384 | 17.788 109.024 |
|  |  | *surinamensis* | FSCS993-11 | JN242497 | MBCSC:Fish:ZH11518152 | 21.125 113.42 |
|  |  | *surinamensis* | FSCS992-11 | JN242496 | MBCSC:Fish:ZH11518140 | 21.125 113.42 |
| Lutjanidae | *Caesio* | *caerulaurea* | CFCS048-08 | FJ237659 | MBCSC:Fish:HN SY08364 | 18.216 109.447 |
|  |  | *caerulaurea* | CFCS272-08 | FJ237658 | MBCSC:Fish:HN SY08588 | 18.234 109.455 |
|  |  | *caerulaurea* | CFCS273-08 | FJ237657 | MBCSC:Fish:HN SY08589 | 18.234 109.455 |
|  |  | *caerulaurea* | CFCS274-08 | FJ237656 | MBCSC:Fish:HN SY08590 | 18.234 109.455 |
|  |  | *caerulaurea* | CFCS275-08 | FJ237655 | MBCSC:Fish:HN SY08591 | 18.234 109.455 |
|  |  | *caerulaurea* | CFCS276-08 | FJ237654 | MBCSC:Fish:HN SY08592 | 18.234 109.455 |
|  |  | *caerulaurea* | CFCS277-08 | FJ237653 | MBCSC:Fish:HN SY08593 | 18.234 109.455 |
|  |  | *caerulaurea* | CFCS278-08 | FJ237652 | MBCSC:Fish:HN SY08594 | 18.234 109.455 |
|  | *Lutjanus* | *argentimaculatus* | FSCS479-07 | EU595190 | MBCSC:Fish:ZC I07138 | 19.366 113.422 |
|  |  | *argentimaculatus* | FSCS480-07 | EU595189 | MBCSC:Fish:ZC I07139 | 9.286 109.438 |
|  |  | *argentimaculatus* | FSCS791-08 | FJ238006 | MBCSC:Fish:ZC I07366 | 20.875 111.21 |
|  |  | *bohar* | FSCS626-07 | EU595191 | MBCSC:Fish:ZC I07298 | 13.734 113.865 |
|  |  | *erythropterus* | FSCS621-07 | EU595214 | MBCSC:Fish:ZC I07293 | 16.643 113.336 |
|  |  | *erythropterus* | FSCS627-07 | EU595213 | MBCSC:Fish:ZC I07299 | 23.967 118.067 |
|  |  | *erythropterus* | FSCS628-07 | EU595212 | MBCSC:Fish:ZC I07300 | 23.967 118.067 |
|  |  | *erythropterus* | FSCS629-07 | EU595211 | MBCSC:Fish:ZC I07301 | 15.117 116.867 |
|  |  | *erythropterus* | FSCS630-07 | EU595210 | MBCSC:Fish:ZC I07302 | 15.117 116.867 |
|  |  | *erythropterus* | FSCS631-07 | EU595209 | MBCSC:Fish:ZC I07303 | 23.967 118.067 |
|  |  | *erythropterus* | FSCS632-07 | EU595208 | MBCSC:Fish:ZC I07304 | 4.9 112.717 |
|  |  | *erythropterus* | FSCS633-07 | EU595207 | MBCSC:Fish:ZC I07305 | 8.233 109.15 |
|  |  | *erythropterus* | FSCS634-07 | EU595206 | MBCSC:Fish:ZC I07306 | 23.967 118.037 |
|  |  | *erythropterus* | FSCS635-07 | EU595205 | MBCSC:Fish:ZC I07307 | 8.833 112.353 |
|  |  | *erythropterus* | FSCS636-07 | EU595204 | MBCSC:Fish:ZC I07308 | 8.833 112.353 |
|  |  | *erythropterus* | FSCS637-07 | EU595203 | MBCSC:Fish:ZC I07309 | 8.833 112.353 |
|  |  | *erythropterus* | FSCS638-07 | EU595202 | MBCSC:Fish:ZC I07310 | 8.233 109.25 |
|  |  | *erythropterus* | FSCS639-07 | EU595201 | MBCSC:Fish:ZC I07311 | 18.033 111.817 |
|  |  | *erythropterus* | FSCS640-07 | EU595200 | MBCSC:Fish:ZC I07312 | 18.033 111.883 |
|  |  | *erythropterus* | FSCS641-07 | EU595199 | MBCSC:Fish:ZC I07313 | 15.117 111.867 |
|  |  | *erythropterus* | FSCS642-07 | EU595198 | MBCSC:Fish:ZC I07314 | 15.111 116.867 |
|  |  | *erythropterus* | FSCS643-07 | EU595197 | MBCSC:Fish:ZC I07315 | 15.2 111.817 |
|  |  | *erythropterus* | FSCS644-07 | EU595196 | MBCSC:Fish:ZC I07316 | 15.2 111.817 |
|  |  | *erythropterus* | FSCS645-07 | EU595195 | MBCSC:Fish:ZC I07317 | 15.2 111.817 |
|  |  | *erythropterus* | FSCS646-07 | EU595194 | MBCSC:Fish:ZC I07318 | 8.233 109.25 |
|  |  | *erythropterus* | FSCS647-07 | EU595193 | MBCSC:Fish:ZC I07319 | 4.9 112.717 |
|  |  | *erythropterus* | FSCS648-07 | EU595192 | MBCSC:Fish:ZC I07320 | 4.9 112.717 |
|  |  | *johnii* | FSCS229-06 | EF607441 | MBCSC:Fish:GD 9081077 | 20.454 110.717 |
|  |  | *lutjanus* | CFCS099-08 | FJ237812 | MBCSC:Fish:HN SY08415 | 16.583 112.756 |
|  |  | *lutjanus* | CFCS132-08 | FJ237811 | MBCSC:Fish:HN SY08448 | 16.378 112.452 |
|  |  | *lutjanus* | CFCS133-08 | FJ237810 | MBCSC:Fish:HN SY08449 | 16.378 112.452 |
|  |  | *lutjanus* | CFCS137-08 | FJ237809 | MBCSC:Fish:HN SY08453 | 16.867 111.36 |
|  |  | *lutjanus* | FSCS218-06 | EF607554 | MBCSC:Fish:GD 9081066 | 20.454 110.717 |
|  |  | *lutjanus* | FSCS219-06 | EF607553 | MBCSC:Fish:GD 9081067 | 20.917 110.542 |
|  |  | *lutjanus* | FSCS220-06 | EF607552 | MBCSC:Fish:GD 9081068 | 20.454 110.717 |
|  |  | *lutjanus* | FSCS221-06 | EF607551 | MBCSC:Fish:GD 9081069 | 20.917 110.542 |
|  |  | *rivulatus* | FSCS622-07 | EU595215 | MBCSC:Fish:ZC I07294 | 16.478 110.535 |
|  |  | *russellii* | FSCS618-07 | EU595217 | MBCSC:Fish:ZC I07290 | 17.175 110.775 |
|  |  | *russellii* | FSCS624-07 | EU595216 | MBCSC:Fish:ZC I07296 | 16.643 113.336 |
|  |  | *sebae* | FSCS620-07 | EU595218 | MBCSC:Fish:ZC I07292 | 16.643 113.336 |
|  |  | *sebae* | FSCS837-08 | FJ238007 | MBCSC:Fish:ZC I07412 | 20.55 111.2 |
|  |  | *sp.* | FSCS617-07 | EU595219 | MBCSC:Fish:ZC I07289 | 17.175 110.775 |
|  | *Pristipomoides* | *multidens* | FSCS1080-11 | JN242629 | MBCSC:Fish:ZH1151894 | 21.125 113.42 |
|  |  | *multidens* | FSCS1079-11 | JN242628 | MBCSC:Fish:XS116985 | 17.311 113.276 |
|  |  | *multidens* | FSCS1078-11 | JN242627 | MBCSC:Fish:ZP1141227 | 21.333 111.977 |
|  |  | *multidens* | FSCS1077-11 | JN242626 | MBCSC:Fish:ZP1141226 | 21.333 111.977 |
|  |  | *multidens* | FSCS1076-11 | JN242625 | MBCSC:Fish:LCG116319 | 23.125 117.876 |
|  |  | *multidens* | FSCS1075-11 | JN242624 | MBCSC:Fish:TCL116468 | 21.121 110.921 |
|  |  | *multidens* | FSCS1074-11 | JN242623 | MBCSC:Fish:ZH1151813 | 21.125 113.42 |
| Malacanthidae | *Branchiostegus* | *argentatus* | CFCS083-08 | FJ237649 | MBCSC:Fish:HN SY08399 | 18.234 109.455 |
|  |  | *argentatus* | CFCS084-08 | FJ237648 | MBCSC:Fish:HN SY08400 | 17.788 109.024 |
|  |  | *argentatus* | CFCS085-08 | FJ237647 | MBCSC:Fish:HN SY08401 | 16.378 112.452 |
|  |  | *argentatus* | CFCS086-08 | FJ237646 | MBCSC:Fish:HN SY08402 | 16.583 112.756 |
|  |  | *argentatus* | CFCS087-08 | FJ237645 | MBCSC:Fish:HN SY08403 | 16.583 112.756 |
|  |  | *argentatus* | CFCS088-08 | FJ237644 | MBCSC:Fish:HN SY08404 | 16.583 112.756 |
|  |  | *argentatus* | CFCS339-08 | FJ237643 | MBCSC:Fish:HN SY08655 | 16.583 112.756 |
|  |  | *argentatus* | CFCS340-08 | FJ237642 | MBCSC:Fish:HN SY08656 | 16.583 112.756 |
|  |  | *argentatus* | CFCS341-08 | FJ237641 | MBCSC:Fish:HN SY08657 | 16.583 112.756 |
|  |  | *argentatus* | CFCS342-08 | FJ237640 | MBCSC:Fish:HN SY08658 | 16.583 112.756 |
|  |  | *argentatus* | CFCS343-08 | FJ237639 | MBCSC:Fish:HN SY08659 | 16.583 112.756 |
|  |  | *argentatus* | CFCS344-08 | FJ237638 | MBCSC:Fish:HN SY08660 | 16.583 112.756 |
|  |  | *argentatus* | CFCS345-08 | FJ237637 | MBCSC:Fish:HN SY08661 | 16.583 112.756 |
|  |  | *argentatus* | CFCS346-08 | FJ237636 | MBCSC:Fish:HN SY08662 | 16.583 112.756 |
|  |  | *argentatus* | CFCS347-08 | FJ237635 | MBCSC:Fish:HN SY08663 | 16.583 112.756 |
|  |  | *argentatus* | CFCS348-08 | FJ237634 | MBCSC:Fish:HN SY08664 | 16.583 112.756 |
| Menidae | *Mene* | *maculata* | FSCS001-06 | EF607445 | MBCSC:Fish:GD 9085001 | 20.476 109.825 |
|  |  | *maculata* | FSCS002-06 | EF607444 | MBCSC:Fish:GD 9085002 | 20.476 109.825 |
|  |  | *maculata* | FSCS003-06 | EF607443 | MBCSC:Fish:GD 9085003 | 20.454 109.889 |
|  |  | *maculata* | FSCS004-06 | EF607442 | MBCSC:Fish:GD 9085004 | 20.454 109.889 |
|  |  | *maculata* | FSCS1000-11 | JN242548 | MBCSC:Fish:ZP1141220 | 21.333 111.977 |
|  |  | *maculata* | FSCS999-11 | JN242547 | MBCSC:Fish:BH1122673 | 21.232 109.377 |
|  |  | *maculata* | FSCS998-11 | JN242546 | MBCSC:Fish:ZH1151818 | 21.125 113.42 |
|  |  | *maculata* | FSCS997-11 | JN242545 | MBCSC:Fish:BH11226138 | 21.232 109.377 |
|  |  | *maculata* | FSCS996-11 | JN242544 | MBCSC:Fish:BH1122641 | 21.232 109.377 |
|  |  | *maculata* | FSCS995-11 | JN242543 | MBCSC:Fish:LCG11509C | 23.459 117.599 |
|  |  | *maculata* | FSCS994-11 | JN242542 | MBCSC:Fish:XS116957 | 17.311 113.276 |
| Monacanthidae | *Monacanthus* | *chinensis* | FSCS185-06 | EF607587 | MBCSC:Fish:GD 9081033 | 20.808 110.6 |
|  |  | *chinensis* | FSCS278-06 | EF607586 | MBCSC:Fish:GD 9082021 | 21.047 110.665 |
|  |  | *chinensis* | FSCS481-07 | EU595322 | MBCSC:Fish:ZC I07140 | 20.045 111.484 |
|  | *Paramonacanthus* | *sulcatus* | FSCS101-06 | EF607475 | MBCSC:Fish:GD 9085031 | 20.416 109.841 |
|  |  | *sulcatus* | FSCS102-06 | EF607474 | MBCSC:Fish:GD 9085032 | 20.476 109.825 |
|  |  | *sulcatus* | FSCS103-06 | EF607473 | MBCSC:Fish:GD 9085033 | 20.476 109.825 |
|  |  | *sulcatus* | FSCS104-06 | EF607472 | MBCSC:Fish:GD 9085034 | 20.416 109.841 |
|  |  | *sulcatus* | FSCS105-06 | EF607471 | MBCSC:Fish:GD 9085035 | 20.476 109.825 |
|  |  | *sulcatus* | FSCS106-06 | EF607470 | MBCSC:Fish:GD 9085036 | 20.476 109.825 |
|  |  | *sulcatus* | FSCS183-06 | EF607469 | MBCSC:Fish:GD 9081031 | 20.808 110.6 |
|  |  | *sulcatus* | FSCS184-06 | EF607468 | MBCSC:Fish:GD 9081032 | 20.767 110.675 |
|  |  | *sulcatus* | FSCS243-06 | EF607467 | MBCSC:Fish:GD 9083004 | 21.573 111.727 |
|  | *Thamnaconus* | *modestoides* | FSCS1141-11 | JN242524 | MBCSC:Fish:XS116930 | 17.311 113.276 |
|  |  | *modestoides* | FSCS1140-11 | JN242523 | MBCSC:Fish:XS116927 | 17.311 113.276 |
|  |  | *modestoides* | FSCS1139-11 | JN242522 | MBCSC:Fish:ZP1141252 | 21.333 111.977 |
|  |  | *modestoides* | FSCS1138-11 | JN242521 | MBCSC:Fish:LCG1132517 | 23.666 118.05 |
|  |  | *modestoides* | FSCS1137-11 | JN242520 | MBCSC:Fish:BH1122619 | 21.232 109.377 |
|  |  | *modestoides* | FSCS1136-11 | JN242519 | MBCSC:Fish:BH1122618 | 21.232 109.377 |
|  |  | *septentrionalis* | FSCS107-06 | EF607585 | MBCSC:Fish:GD 9085037 | 20.416 109.841 |
|  |  | *septentrionalis* | FSCS108-06 | EF607584 | MBCSC:Fish:GD 9085038 | 20.454 109.889 |
|  |  | *septentrionalis* | FSCS186-06 | EF607583 | MBCSC:Fish:GD 9081034 | 20.917 110.542 |
| Monodactylidae | *Monodactylus* | *argenteus* | CFCS077-08 | FJ237826 | MBCSC:Fish:HN SY08393 | 17.788 109.024 |
|  |  | *argenteus* | CFCS078-08 | FJ237825 | MBCSC:Fish:HN SY08394 | 16.583 112.756 |
|  |  | *argenteus* | CFCS079-08 | FJ237824 | MBCSC:Fish:HN SY08395 | 16.583 112.756 |
|  |  | *argenteus* | CFCS080-08 | FJ237823 | MBCSC:Fish:HN SY08396 | 16.583 112.756 |
|  |  | *argenteus* | CFCS081-08 | FJ237822 | MBCSC:Fish:HN SY08397 | 17.788 109.024 |
|  |  | *argenteus* | CFCS330-08 | FJ237821 | MBCSC:Fish:HN SY08646 | 17.788 109.024 |
|  |  | *argenteus* | CFCS331-08 | FJ237820 | MBCSC:Fish:HN SY08647 | 17.788 109.024 |
|  |  | *argenteus* | CFCS332-08 | FJ237819 | MBCSC:Fish:HN SY08648 | 17.788 109.024 |
|  |  | *argenteus* | CFCS333-08 | FJ237818 | MBCSC:Fish:HN SY08649 | 17.788 109.024 |
|  |  | *argenteus* | CFCS334-08 | FJ237817 | MBCSC:Fish:HN SY08650 | 17.788 109.024 |
|  |  | *argenteus* | CFCS335-08 | FJ237816 | MBCSC:Fish:HN SY08651 | 16.583 112.756 |
|  |  | *argenteus* | CFCS336-08 | FJ237815 | MBCSC:Fish:HN SY08652 | 16.867 111.36 |
|  |  | *argenteus* | CFCS337-08 | FJ237814 | MBCSC:Fish:HN SY08653 | 16.867 111.36 |
|  |  | *argenteus* | CFCS338-08 | FJ237813 | MBCSC:Fish:HN SY08654 | 16.583 112.756 |
| Mugilidae | *Liza* | *sp.* | FSCS303-06 | EF607447 | MBCSC:Fish:GD 9087014 | 21.42 111.169 |
|  |  | *sp.* | FSCS304-06 | EF607446 | MBCSC:Fish:GD 9087015 | 21.435 111.128 |
|  |  | *haematocheilus* | FSCS990-11 | JN242619 | MBCSC:Fish:LCG1141841 | 23.5 117.666 |
|  |  | *haematocheilus* | FSCS989-11 | JN242618 | MBCSC:Fish:ZH1151821 | 21.125 113.42 |
|  |  | *haematocheilus* | FSCS988-11 | JN242617 | MBCSC:Fish:XS116998 | 17.311 113.276 |
|  |  | *haematocheilus* | FSCS987-11 | JN242616 | MBCSC:Fish:BH1122650 | 21.232 109.377 |
|  |  | *haematocheilus* | FSCS986-11 | JN242615 | MBCSC:Fish:BH1122649 | 21.232 109.377 |
|  | *Mugil* | *cephalus* | FSCS559-07 | EU595085 | MBCSC:Fish:ZC I07228 | 21.091 112.553 |
|  |  | *cephalus* | FSCS560-07 | EU595084 | MBCSC:Fish:ZC I07229 | 20.045 111.484 |
|  |  | *cephalus* | FSCS1019-11 | JN242570 | MBCSC:Fish:ZP1141223 | 21.333 111.977 |
|  |  | *cephalus* | FSCS1018-11 | JN242569 | MBCSC:Fish:ZP1141222 | 21.333 111.977 |
|  |  | *cephalus* | FSCS1017-11 | JN242568 | MBCSC:Fish:LCG116459 | 23.45 117.379 |
|  |  | *cephalus* | FSCS1016-11 | JN242567 | MBCSC:Fish:ZP1141255 | 21.333 111.977 |
|  |  | *cephalus* | FSCS1015-11 | JN242566 | MBCSC:Fish:XS1169156 | 17.311 113.276 |
|  |  | *cephalus* | FSCS1014-11 | JN242565 | MBCSC:Fish:LCG116405 | 23.45 117.379 |
|  |  | *cephalus* | FSCS1013-11 | JN242571 | MBCSC:Fish:ZP11412147 | 21.333 111.977 |
|  | *Valamugil* | *engeli* | FSCS251-06 | EF607615 | MBCSC:Fish:GD 9083012 | 21.551 111.987 |
|  |  | *cunnesius* | FSCS580-07 | EU595343 | MBCSC:Fish:ZC I07250 | 20.516 110.981 |
|  |  | *cunnesius* | FSCS581-07 | EU595342 | MBCSC:Fish:ZC I07251 | 20.516 110.981 |
|  |  | *cunnesius* | FSCS582-07 | EU595341 | MBCSC:Fish:ZC I07252 | 19.685 112.767 |
|  |  | *cunnesius* | FSCS583-07 | EU595340 | MBCSC:Fish:ZC I07253 | 19.685 112.767 |
|  |  | *cunnesius* | FSCS584-07 | EU595339 | MBCSC:Fish:ZC I07254 | 21.074 111.557 |
|  |  | *cunnesius* | FSCS585-07 | EU595338 | MBCSC:Fish:ZC I07255 | 21.074 111.557 |
|  |  | *cunnesius* | FSCS806-08 | FJ238048 | MBCSC:Fish:ZC I07381 | 20.55 111.2 |
| Mullidae | *Mulloidichthys* | *vanicolensis* | CFCS104-08 | FJ237829 | MBCSC:Fish:HN SY08420 | 16.677 112.898 |
|  |  | *vanicolensis* | CFCS157-08 | FJ237828 | MBCSC:Fish:HN SY08473 | 17.073 109.736 |
|  |  | *vanicolensis* | CFCS158-08 | FJ237827 | MBCSC:Fish:HN SY08474 | 17.073 109.736 |
|  |  | *vanicolensis* | FSCS130-06 | EF607607 | MBCSC:Fish:GD 9085060 | 20.476 109.825 |
|  | *Parupeneus* | *ciliatus* | FSCS210-06 | EF607486 | MBCSC:Fish:GD 9081058 | 20.454 110.717 |
|  |  | *ciliatus* | FSCS211-06 | EF607485 | MBCSC:Fish:GD 9081059 | 20.454 110.717 |
|  |  | *indicus* | CFCS101-08 | FJ237883 | MBCSC:Fish:HN SY08417 | 16.677 112.898 |
|  |  | *indicus* | CFCS102-08 | FJ237882 | MBCSC:Fish:HN SY08418 | 18.234 109.455 |
|  |  | *indicus* | CFCS103-08 | FJ237881 | MBCSC:Fish:HN SY08419 | 16.677 112.898 |
|  |  | *indicus* | CFCS142-08 | FJ237880 | MBCSC:Fish:HN SY08458 | 16.867 111.36 |
|  |  | *indicus* | CFCS143-08 | FJ237879 | MBCSC:Fish:HN SY08459 | 16.378 112.452 |
|  |  | *indicus* | CFCS144-08 | FJ237878 | MBCSC:Fish:HN SY08460 | 18.234 109.455 |
|  |  | *indicus* | CFCS145-08 | FJ237877 | MBCSC:Fish:HN SY08461 | 18.234 109.455 |
|  |  | *indicus* | CFCS148-08 | FJ237876 | MBCSC:Fish:HN SY08464 | 17.073 109.736 |
|  |  | *indicus* | CFCS149-08 | FJ237875 | MBCSC:Fish:HN SY08465 | 17.073 109.736 |
|  |  | *indicus* | CFCS150-08 | FJ237874 | MBCSC:Fish:HN SY08466 | 17.073 109.736 |
|  |  | *indicus* | CFCS151-08 | FJ237873 | MBCSC:Fish:HN SY08467 | 17.073 109.736 |
|  |  | *indicus* | CFCS155-08 | FJ237872 | MBCSC:Fish:HN SY08471 | 17.455 109.833 |
|  |  | *indicus* | CFCS156-08 | FJ237871 | MBCSC:Fish:HN SY08472 | 17.073 109.736 |
|  |  | *multifasciatus* | CFCS100-08 | FJ237888 | MBCSC:Fish:HN SY08416 | 16.583 112.756 |
|  |  | *multifasciatus* | CFCS117-08 | FJ237887 | MBCSC:Fish:HN SY08433 | 16.378 112.452 |
|  |  | *multifasciatus* | CFCS140-08 | FJ237886 | MBCSC:Fish:HN SY08456 | 16.867 111.36 |
|  |  | *multifasciatus* | CFCS141-08 | FJ237885 | MBCSC:Fish:HN SY08457 | 16.867 111.36 |
|  |  | *multifasciatus* | CFCS212-08 | FJ237884 | MBCSC:Fish:HN SY08528 | 17.073 109.736 |
|  | *Upeneus* | *japonicus* | FSCS126-06 | EF607606 | MBCSC:Fish:GD 9085056 | 20.454 109.889 |
|  |  | *japonicus* | FSCS127-06 | EF607605 | MBCSC:Fish:GD 9085057 | 20.476 109.825 |
|  |  | *japonicus* | FSCS128-06 | EF607604 | MBCSC:Fish:GD 9085058 | 20.416 109.841 |
|  |  | *japonicus* | FSCS129-06 | EF607603 | MBCSC:Fish:GD 9085059 | 20.476 109.825 |
|  |  | *japonicus* | FSCS245-06 | EF607602 | MBCSC:Fish:GD 9083006 | 21.551 111.987 |
|  |  | *sulphureus* | FSCS171-06 | EF607610 | MBCSC:Fish:GD 9081019 | 20.767 110.675 |
|  |  | *sulphureus* | FSCS045-06 | EF607609 | MBCSC:Fish:GD 9086020 | 20.416 109.841 |
|  |  | *sulphureus* | FSCS046-06 | EF607608 | MBCSC:Fish:GD 9086021 | 20.476 109.825 |
|  |  | *sulphureus* | FSCS484-07 | EU595334 | MBCSC:Fish:ZC I07143 | 19.685 112.767 |
|  |  | *sulphureus* | FSCS485-07 | EU595333 | MBCSC:Fish:ZC I07144 | 19.685 112.767 |
|  |  | *sulphureus* | FSCS486-07 | EU595332 | MBCSC:Fish:ZC I07145 | 19.685 112.767 |
|  |  | *sulphureus* | FSCS487-07 | EU595331 | MBCSC:Fish:ZC I07146 | 19.685 112.767 |
|  |  | *sulphureus* | FSCS488-07 | EU595330 | MBCSC:Fish:ZC I07147 | 19.685 112.767 |
|  |  | *sulphureus* | FSCS489-07 | EU595329 | MBCSC:Fish:ZC I07148 | 19.685 112.767 |
|  |  | *sulphureus* | FSCS490-07 | EU595328 | MBCSC:Fish:ZC I07149 | 21.007 111.223 |
|  |  | *sulphureus* | FSCS491-07 | EU595327 | MBCSC:Fish:ZC I07150 | 21.007 111.223 |
|  |  | *tragula* | FSCS207-06 | EF607613 | MBCSC:Fish:GD 9081055 | 20.767 110.675 |
|  |  | *tragula* | FSCS208-06 | EF607612 | MBCSC:Fish:GD 9081056 | 20.454 110.717 |
|  |  | *tragula* | FSCS209-06 | EF607611 | MBCSC:Fish:GD 9081057 | 20.917 110.542 |
|  |  | *tragula* | FSCS279-06 | EF607614 | MBCSC:Fish:GD 9082022 | 21.047 110.665 |
| Muraenesocidae | *Oxyconger* | *leptognathus* | FSCS144-06 | EF607454 | MBCSC:Fish:GD 9085074 | 20.476 109.825 |
|  |  | *leptognathus* | FSCS145-06 | EF607453 | MBCSC:Fish:GD 9085075 | 20.476 109.825 |
|  |  | *leptognathus* | FSCS146-06 | EF607452 | MBCSC:Fish:GD 9085076 | 20.313 109.858 |
|  | *Muraenesox* | *cinereus* | FSCS234-06 | EF607449 | MBCSC:Fish:GD 9081082 | 20.454 110.717 |
|  |  | *cinereus* | FSCS288-06 | EF607448 | MBCSC:Fish:GD 9082031 | 21.047 110.665 |
| Muraenidae | *Gymnothorax* | *pseudothyrsoideus* | FSCS441-07 | EU595143 | MBCSC:Fish:ZC I07102 | 19.685 112.767 |
|  |  | *pseudothyrsoideus* | FSCS748-08 | FJ237991 | MBCSC:Fish:ZC I07323 | 20.65 111.487 |
|  |  | *pseudothyrsoideus* | FSCS749-08 | FJ237990 | MBCSC:Fish:ZC I07324 | 20.65 111.487 |
|  |  | *reevesii* | FSCS147-06 | EF607397 | MBCSC:Fish:GD 9085077 | 20.476 109.825 |
|  |  | *reevesii* | FSCS188-06 | EF607396 | MBCSC:Fish:GD 9081036 | 20.808 110.6 |
|  |  | *reevesii* | FSCS189-06 | EF607395 | MBCSC:Fish:GD 9081037 | 20.702 110.564 |
|  |  | *reevesii* | FSCS475-07 | EU595145 | MBCSC:Fish:ZC I07135 | 20.045 111.484 |
|  |  | *reevesii* | FSCS476-07 | EU595144 | MBCSC:Fish:ZC I07136 | 15.267 112.335 |
|  |  | *reevesii* | FSCS790-08 | FJ237992 | MBCSC:Fish:ZC I07365 | 20.875 111.21 |
|  |  | *undulatus* | FSCS440-07 | EU595146 | MBCSC:Fish:ZC I07092 | 19.366 113.422 |
| Narcinidae | *Narcine* | *brevilabiata* | CFCS011-08 | FJ237832 | MBCSC:Fish:HN SY08327 | 16.583 112.756 |
|  |  | *brevilabiata* | CFCS161-08 | FJ237831 | MBCSC:Fish:HN SY08477 | 17.455 109.833 |
|  |  | *brevilabiata* | CFCS162-08 | FJ237830 | MBCSC:Fish:HN SY08478 | 17.455 109.833 |
| Nemipteridae | *Nemipterus* | *virgatus* | CFCS013-08 | FJ237848 | MBCSC:Fish:HN SY08329 | 18.234 109.455 |
|  |  | *virgatus* | CFCS014-08 | FJ237847 | MBCSC:Fish:HN SY08330 | 17.073 109.736 |
|  |  | *virgatus* | CFCS015-08 | FJ237846 | MBCSC:Fish:HN SY08331 | 18.234 109.455 |
|  |  | *virgatus* | CFCS016-08 | FJ237845 | MBCSC:Fish:HN SY08332 | 16.035 114.134 |
|  |  | *virgatus* | CFCS017-08 | FJ237844 | MBCSC:Fish:HN SY08333 | 18.234 109.455 |
|  |  | *virgatus* | CFCS167-08 | FJ237843 | MBCSC:Fish:HN SY08483 | 17.073 109.736 |
|  |  | *virgatus* | CFCS168-08 | FJ237842 | MBCSC:Fish:HN SY08484 | 17.455 109.833 |
|  |  | *virgatus* | CFCS174-08 | FJ237841 | MBCSC:Fish:HN SY08490 | 17.073 109.736 |
|  |  | *virgatus* | CFCS175-08 | FJ237840 | MBCSC:Fish:HN SY08491 | 17.073 109.736 |
|  |  | *virgatus* | CFCS176-08 | FJ237839 | MBCSC:Fish:HN SY08492 | 18.237 108.161 |
|  |  | *virgatus* | CFCS177-08 | FJ237838 | MBCSC:Fish:HN SY08493 | 18.234 109.455 |
|  |  | *virgatus* | CFCS178-08 | FJ237837 | MBCSC:Fish:HN SY08494 | 18.234 109.455 |
|  |  | *virgatus* | CFCS179-08 | FJ237836 | MBCSC:Fish:HN SY08495 | 18.234 109.455 |
|  |  | *virgatus* | CFCS180-08 | FJ237835 | MBCSC:Fish:HN SY08496 | 18.234 109.455 |
|  |  | *virgatus* | CFCS181-08 | FJ237834 | MBCSC:Fish:HN SY08497 | 18.234 109.455 |
|  |  | *virgatus* | CFCS182-08 | FJ237833 | MBCSC:Fish:HN SY08498 | 18.234 109.455 |
| Ophidiidae | *Brotula* | *multibarbata* | CFCS118-08 | FJ237651 | MBCSC:Fish:HN SY08434 | 18.216 109.447 |
|  |  | *multibarbata* | CFCS216-08 | FJ237650 | MBCSC:Fish:HN SY08532 | 18.234 109.455 |
| Paralichthyidae | *Pseudorhombus* | *arsius* | CFCS043-08 | FJ237895 | MBCSC:Fish:HN SY08359 | 18.216 109.447 |
|  |  | *arsius* | CFCS263-08 | FJ237894 | MBCSC:Fish:HN SY08579 | 18.234 109.455 |
|  |  | *arsius* | CFCS264-08 | FJ237893 | MBCSC:Fish:HN SY08580 | 18.234 109.455 |
|  |  | *arsius* | CFCS265-08 | FJ237892 | MBCSC:Fish:HN SY08581 | 18.234 109.455 |
|  |  | *arsius* | CFCS266-08 | FJ237891 | MBCSC:Fish:HN SY08582 | 18.234 109.455 |
| Peristediidae | *Satyrichthys* | *amiscus* | CFCS047-08 | FJ237914 | MBCSC:Fish:HN SY08363 | 16.035 114.134 |
| Pinguipedidae | *Parapercis* | *ommatura* | FSCS040-06 | EF607479 | MBCSC:Fish:GD 9086015 | 20.454 109.889 |
|  |  | *ommatura* | FSCS041-06 | EF607478 | MBCSC:Fish:GD 9086016 | 20.416 109.841 |
|  |  | *ommatura* | FSCS042-06 | EF607477 | MBCSC:Fish:GD 9086017 | 20.313 109.858 |
|  |  | *ommatura* | FSCS043-06 | EF607476 | MBCSC:Fish:GD 9086018 | 20.454 109.889 |
| Platycephalidae | *Kumococius* | *rodericensis* | FSCS132-06 | EF607415 | MBCSC:Fish:GD 9085062 | 20.476 109.825 |
|  |  | *rodericensis* | FSCS133-06 | EF607414 | MBCSC:Fish:GD 9085063 | 20.476 109.825 |
|  |  | *rodericensis* | FSCS134-06 | EF607413 | MBCSC:Fish:GD 9085064 | 20.454 109.889 |
|  |  | *rodericensis* | FSCS500-07 | EU595159 | MBCSC:Fish:ZC I07159 | 21.091 112.553 |
|  |  | *rodericensis* | FSCS501-07 | EU595158 | MBCSC:Fish:ZC I07160 | 21.091 112.553 |
|  | *Platycephalus* | *indicus* | FSCS408-07 | EU595229 | MBCSC:Fish:ZC I07061 | 19.685 112.767 |
|  |  | *indicus* | FSCS409-07 | EU595228 | MBCSC:Fish:ZC I07062 | 19.366 113.422 |
|  |  | *indicus* | FSCS410-07 | EU595227 | MBCSC:Fish:ZC I07063 | 21.007 111.223 |
|  |  | *indicus* | FSCS411-07 | EU595226 | MBCSC:Fish:ZC I07064 | 20.516 110.981 |
|  |  | *indicus* | FSCS808-08 | FJ238010 | MBCSC:Fish:ZC I07383 | 20.65 111.487 |
|  |  | *indicus* | FSCS810-08 | FJ238009 | MBCSC:Fish:ZC I07385 | 20.55 111.2 |
|  |  | *sp.* | FSCS135-06 | EF607489 | MBCSC:Fish:GD 9085065 | 20.454 109.889 |
| Plotosidae | *Plotosus* | *lineatus* | FSCS284-06 | EF607324 | MBCSC:Fish:GD 9082027 | 21.047 110.665 |
|  |  | *lineatus* | FSCS285-06 | EF607323 | MBCSC:Fish:GD 9082028 | 21.089 110.452 |
|  |  | *lineatus* | FSCS384-07 | EU595239 | MBCSC:Fish:ZC I07045 | 21.007 111.223 |
|  |  | *lineatus* | FSCS385-07 | EU595238 | MBCSC:Fish:ZC I07046 | 21.007 111.223 |
|  |  | *lineatus* | FSCS386-07 | EU595237 | MBCSC:Fish:ZC I07047 | 19.685 112.767 |
|  |  | *lineatus* | FSCS387-07 | EU595236 | MBCSC:Fish:ZC I07048 | 19.685 112.767 |
|  |  | *lineatus* | FSCS388-07 | EU595235 | MBCSC:Fish:ZC I07049 | 20.516 110.981 |
|  |  | *lineatus* | FSCS389-07 | EU595234 | MBCSC:Fish:ZC I07050 | 20.516 110.981 |
|  |  | *lineatus* | FSCS798-08 | FJ238012 | MBCSC:Fish:ZC I07373 | 20.65 111.487 |
| Polynemidae | *Eleutheronema* | *tetradactylum* | FSCS544-07 | EU595108 | MBCSC:Fish:ZC I07220 | 19.685 112.767 |
|  |  | *tetradactylum* | FSCS545-07 | EU595107 | MBCSC:Fish:ZC I07221 | 19.685 112.767 |
|  |  | *tetradactylum* | FSCS546-07 | EU595106 | MBCSC:Fish:ZC I07222 | 19.685 112.767 |
|  |  | *tetradactylum* | FSCS547-07 | EU595105 | MBCSC:Fish:ZC I07213 | 21.091 112.553 |
|  |  | *tetradactylum* | FSCS548-07 | EU595104 | MBCSC:Fish:ZC I07214 | 16.256 114.883 |
|  |  | *tetradactylum* | FSCS549-07 | EU595103 | MBCSC:Fish:ZC I07215 | 11.058 115.021 |
|  |  | *tetradactylum* | FSCS767-08 | FJ237985 | MBCSC:Fish:ZC I07342 | 20.875 111.21 |
|  | *Polydactylus* | *sextarius* | FSCS305-06 | EF607490 | MBCSC:Fish:GD 9087016 | 21.435 111.128 |
|  |  | *sextarius* | FSCS492-07 | EU595242 | MBCSC:Fish:ZC I07151 | 21.091 112.553 |
|  |  | *sextarius* | FSCS493-07 | EU595241 | MBCSC:Fish:ZC I07152 | 21.091 112.553 |
|  |  | *sextarius* | FSCS494-07 | EU595240 | MBCSC:Fish:ZC I07153 | 19.366 113.422 |
|  |  | *sextarius* | FSCS803-08 | FJ238015 | MBCSC:Fish:ZC I07378 | 20.875 111.21 |
|  |  | *sextarius* | FSCS804-08 | FJ238014 | MBCSC:Fish:ZC I07379 | 20.55 111.2 |
|  |  | *sextarius* | FSCS805-08 | FJ238013 | MBCSC:Fish:ZC I07380 | 20.55 111.2 |
| Pomacentridae | *Abudefduf* | *septemfasciatus* | FSCS199-06 | EF607296 | MBCSC:Fish:GD 9081047 | 20.454 110.717 |
|  |  | *vaigiensis* | CFCS122-08 | FJ237625 | MBCSC:Fish:HN SY08438 | 18.216 109.447 |
|  |  | *vaigiensis* | CFCS123-08 | FJ237624 | MBCSC:Fish:HN SY08439 | 18.216 109.447 |
| Priacanthidae | *Priacanthus* | *macracanthus* | FSCS530-07 | EU595246 | MBCSC:Fish:ZC I07199 | 21.091 112.553 |
|  |  | *macracanthus* | FSCS532-07 | EU595245 | MBCSC:Fish:ZC I07201 | 19.685 112.767 |
|  |  | *tayenus* | FSCS321-06 | EF607497 | MBCSC:Fish:GD 9088009 | 23.296 116.784 |
|  |  | *tayenus* | FSCS322-06 | EF607496 | MBCSC:Fish:GD 9088010 | 23.256 116.833 |
|  |  | *tayenus* | FSCS529-07 | EU595249 | MBCSC:Fish:ZC I07198 | 19.685 112.767 |
|  |  | *tayenus* | FSCS531-07 | EU595248 | MBCSC:Fish:ZC I07200 | 19.685 112.767 |
|  |  | *tayenus* | FSCS533-07 | EU595247 | MBCSC:Fish:ZC I07202 | 21.091 112.553 |
|  |  | *tayenus* | FSCS746-08 | FJ238019 | MBCSC:Fish:ZC I07321 | 20.55 111.2 |
|  |  | *tayenus* | FSCS747-08 | FJ238018 | MBCSC:Fish:ZC I07322 | 20.65 111.487 |
|  |  | *tayenus* | FSCS1073-11 | JN242728 | MBCSC:Fish:BH1122672 | 21.232 109.377 |
|  |  | *tayenus* | FSCS1072-11 | JN242727 | MBCSC:Fish:ZH1151834 | 21.125 113.42 |
|  |  | *tayenus* | FSCS1071-11 | JN242726 | MBCSC:Fish:ZP1141232 | 21.333 111.977 |
|  |  | *tayenus* | FSCS1070-11 | JN242725 | MBCSC:Fish:ZP1141231 | 21.333 111.977 |
|  |  | *tayenus* | FSCS1069-11 | JN242724 | MBCSC:Fish:TCL116424 | 21.121 110.921 |
|  |  | *tayenus* | FSCS1068-11 | JN242723 | MBCSC:Fish:BH1122638 | 21.232 109.377 |
|  |  | *tayenus* | FSCS1067-11 | JN242722 | MBCSC:Fish:BH1122637 | 21.232 109.377 |
| Rachycentridae | *Rachycentron* | *canadum* | FSCS095-06 | EF607498 | MBCSC:Fish:GD 9086070 | 20.313 109.858 |
| Salangidae | *Salanx* | *chinensis* | FSCS1088-11 | JN242664 | MBCSC:Fish:LCG1163116 | 23.125 117.876 |
|  |  | *chinensis* | FSCS1087-11 | JN242663 | MBCSC:Fish:ZH1151815 | 21.125 113.42 |
|  |  | *chinensis* | FSCS1086-11 | JN242662 | MBCSC:Fish:XS116913 | 17.311 113.276 |
|  |  | *chinensis* | FSCS1085-11 | JN242661 | MBCSC:Fish:XS116909 | 17.311 113.276 |
|  |  | *chinensis* | FSCS1084-11 | JN242660 | MBCSC:Fish:ZP1141217 | 21.333 111.977 |
|  |  | *chinensis* | FSCS1083-11 | JN242659 | MBCSC:Fish:ZP1141216 | 21.333 111.977 |
|  |  | *chinensis* | FSCS1082-11 | JN242658 | MBCSC:Fish:LCG1132524 | 23.666 118.05 |
|  |  | *chinensis* | FSCS1081-11 | JN242657 | MBCSC:Fish:BH1122639 | 21.232 109.377 |
| Scaridae | *Calotomus* | *carolinus* | CFCS107-08 | FJ237667 | MBCSC:Fish:HN SY08423 | 16.035 114.134 |
|  |  | *carolinus* | CFCS108-08 | FJ237666 | MBCSC:Fish:HN SY08424 | 16.035 114.134 |
|  |  | *carolinus* | CFCS166-08 | FJ237665 | MBCSC:Fish:HN SY08482 | 17.073 109.736 |
|  |  | *carolinus* | CFCS169-08 | FJ237664 | MBCSC:Fish:HN SY08485 | 17.073 109.736 |
|  |  | *carolinus* | CFCS170-08 | FJ237663 | MBCSC:Fish:HN SY08486 | 17.073 109.736 |
|  |  | *carolinus* | CFCS171-08 | FJ237662 | MBCSC:Fish:HN SY08487 | 17.073 109.736 |
|  |  | *carolinus* | CFCS172-08 | FJ237661 | MBCSC:Fish:HN SY08488 | 17.073 109.736 |
|  |  | *carolinus* | CFCS173-08 | FJ237660 | MBCSC:Fish:HN SY08489 | 17.073 109.736 |
|  | *Chlorurus* | *gibbus* | CFCS105-08 | FJ237699 | MBCSC:Fish:HN SY08421 | 16.677 112.898 |
|  | *Leptoscarus* | *vaigiensis* | CFCS183-08 | FJ237789 | MBCSC:Fish:HN SY08499 | 17.073 109.736 |
|  |  | *vaigiensis* | CFCS311-08 | FJ237788 | MBCSC:Fish:HN SY08627 | 17.788 109.024 |
|  | *Scarus* | *chameleon* | CFCS114-08 | FJ237917 | MBCSC:Fish:HN SY08430 | 16.378 112.452 |
|  |  | *chameleon* | CFCS197-08 | FJ237916 | MBCSC:Fish:HN SY08513 | 17.073 109.736 |
|  |  | *chameleon* | CFCS198-08 | FJ237915 | MBCSC:Fish:HN SY08514 | 17.073 109.736 |
|  |  | *frenatus* | CFCS111-08 | FJ237920 | MBCSC:Fish:HN SY08427 | 17.073 109.736 |
|  |  | *frenatus* | CFCS184-08 | FJ237919 | MBCSC:Fish:HN SY08500 | 17.455 109.833 |
|  |  | *frenatus* | CFCS185-08 | FJ237918 | MBCSC:Fish:HN SY08501 | 17.073 109.736 |
| Scatophagidae | *Scatophagus* | *argus* | FSCS053-06 | EF607518 | MBCSC:Fish:GD 9086028 | 20.313 109.858 |
|  |  | *argus* | FSCS054-06 | EF607517 | MBCSC:Fish:GD 9086029 | 20.313 109.858 |
|  |  | *argus* | FSCS055-06 | EF607516 | MBCSC:Fish:GD 9086030 | 20.313 109.858 |
|  |  | *argus* | FSCS299-06 | EF607519 | MBCSC:Fish:GD 9087010 | 21.42 111.169 |
| Sciaenidae | *Argyrosomus* | *japonicus* | FSCS860-11 | JN242527 | MBCSC:Fish:ZP1141289 | 21.333 111.977 |
|  |  | *japonicus* | FSCS859-11 | JN242526 | MBCSC:Fish:TCL116457 | 21.121 110.921 |
|  |  | *japonicus* | FSCS858-11 | JN242525 | MBCSC:Fish:LCG1151103 | 23.299 117.8 |
|  | *Chrysochir* | *aureus* | FSCS148-06 | EF607347 | MBCSC:Fish:GD 9085078 | 20.454 109.889 |
|  | *Collichthys* | *lucidus* | FSCS971-11 | JN242538 | MBCSC:Fish:LCG1141817 | 23.5 117.666 |
|  |  | *lucidus* | FSCS970-11 | JN242537 | MBCSC:Fish:ZH1151897 | 21.125 113.42 |
|  |  | *lucidus* | FSCS969-11 | JN242536 | MBCSC:Fish:LCG1141812 | 23.5 117.666 |
|  |  | *lucidus* | FSCS968-11 | JN242541 | MBCSC:Fish:ZP1141209 | 21.333 111.977 |
|  |  | *lucidus* | FSCS967-11 | JN242540 | MBCSC:Fish:ZP1141208 | 21.333 111.977 |
|  |  | *lucidus* | FSCS966-11 | JN242539 | MBCSC:Fish:ZP1141207 | 21.333 111.977 |
|  |  | *lucidus* | FSCS965-11 | JN242535 | MBCSC:Fish:BH1122610 | 21.232 109.377 |
|  |  | *lucidus* | FSCS964-11 | JN242534 | MBCSC:Fish:BH1122609 | 21.232 109.377 |
|  |  | *lucidus* | FSCS963-11 | JN242533 | MBCSC:Fish:LCG1141806 | 23.5 117.666 |
|  |  | *lucidus* | FSCS893-11 | JN242475 | MBCSC:Fish:ZH1151861 | 21.125 113.42 |
|  |  | *lucidus* | FSCS892-11 | JN242474 | MBCSC:Fish:LCG116227 | 23.388 117.449 |
|  |  | *lucidus* | FSCS891-11 | JN242473 | MBCSC:Fish:BH1122602 | 21.232 109.377 |
|  |  | *lucidus* | FSCS890-11 | JN242472 | MBCSC:Fish:ZP11412103 | 21.333 111.977 |
|  |  | *lucidus* | FSCS889-11 | JN242471 | MBCSC:Fish:XS1169112 | 17.311 113.276 |
|  |  | *lucidus* | FSCS888-11 | JN242470 | MBCSC:Fish:LCG116401 | 23.45 117.379 |
|  |  | *lucidus* | FSCS887-11 | JN242469 | MBCSC:Fish:XS116976 | 17.311 113.276 |
|  | *Dendrophysa* | *russelii* | FSCS068-06 | EF607359 | MBCSC:Fish:GD 9086043 | 20.476 109.825 |
|  |  | *russelii* | FSCS069-06 | EF607358 | MBCSC:Fish:GD 9086044 | 20.313 109.858 |
|  |  | *russelii* | FSCS070-06 | EF607357 | MBCSC:Fish:GD 9086045 | 20.476 109.825 |
|  |  | *russelii* | FSCS071-06 | EF607356 | MBCSC:Fish:GD 9086046 | 20.476 109.825 |
|  |  | *russelii* | FSCS072-06 | EF607355 | MBCSC:Fish:GD 9086047 | 20.476 109.825 |
|  | *Johnius* | *belangerii* | FSCS200-06 | EF607412 | MBCSC:Fish:GD 9081048 | 20.767 110.675 |
|  |  | *belangerii* | FSCS201-06 | EF607411 | MBCSC:Fish:GD 9081049 | 20.454 110.717 |
|  |  | *belangerii* | FSCS297-06 | EF607410 | MBCSC:Fish:GD 9087008 | 21.435 111.128 |
|  | *Larimichthys* | *crocea* | FSCS346-07 | EU595177 | MBCSC:Fish:ZC I07016 | 21.074 111.557 |
|  |  | *crocea* | FSCS347-07 | EU595176 | MBCSC:Fish:ZC I07017 | 19.366 113.422 |
|  |  | *crocea* | FSCS348-07 | EU595175 | MBCSC:Fish:ZC I07018 | 19.685 112.767 |
|  |  | *crocea* | FSCS349-07 | EU595174 | MBCSC:Fish:ZC I07019 | 20.497 113.568 |
|  |  | *crocea* | FSCS350-07 | EU595173 | MBCSC:Fish:ZC I07020 | 20.516 110.981 |
|  |  | *crocea* | FSCS351-07 | EU595172 | MBCSC:Fish:ZC I07021 | 21.074 111.557 |
|  |  | *crocea* | FSCS352-07 | EU595171 | MBCSC:Fish:ZC I07022 | 19.685 112.767 |
|  |  | *crocea* | FSCS353-07 | EU595170 | MBCSC:Fish:ZC I07057 | 19.366 113.422 |
|  |  | *crocea* | FSCS354-07 | EU595169 | MBCSC:Fish:ZC I07058 | 19.685 112.767 |
|  |  | *crocea* | FSCS598-07 | EU595168 | MBCSC:Fish:ZC I07268 | 21.074 111.557 |
|  |  | *crocea* | FSCS599-07 | EU595167 | MBCSC:Fish:ZC I07269 | 21.074 111.557 |
|  |  | *crocea* | FSCS600-07 | EU595166 | MBCSC:Fish:ZC I07270 | 20.045 111.484 |
|  |  | *crocea* | FSCS601-07 | EU595165 | MBCSC:Fish:ZC I07271 | 20.045 111.484 |
|  |  | *crocea* | FSCS602-07 | EU595164 | MBCSC:Fish:ZC I07272 | 20.045 111.484 |
|  |  | *crocea* | FSCS820-08 | FJ237998 | MBCSC:Fish:ZC I07395 | 19.685 112.767 |
|  |  | *crocea* | FSCS821-08 | FJ237997 | MBCSC:Fish:ZC I07396 | 20.55 111.2 |
|  |  | *polyactis* | FSCS976-11 | JN242495 | MBCSC:Fish:XS116903 | 17.311 113.276 |
|  |  | *polyactis* | FSCS975-11 | JN242494 | MBCSC:Fish:BH1122636 | 21.232 109.377 |
|  |  | *polyactis* | FSCS974-11 | JN242493 | MBCSC:Fish:ZH1151880 | 21.125 113.42 |
|  |  | *polyactis* | FSCS973-11 | JN242492 | MBCSC:Fish:LCG1132514 | 23.666 118.05 |
|  |  | *polyactis* | FSCS972-11 | JN242491 | MBCSC:Fish:ZH1151878 | 21.125 113.42 |
|  | *Miichthys* | *miiuy* | FSCS1012-11 | JN242518 | MBCSC:Fish:XS116987 | 17.311 113.276 |
|  |  | *miiuy* | FSCS1011-11 | JN242517 | MBCSC:Fish:LCG1141851 | 23.5 117.666 |
|  |  | *miiuy* | FSCS1010-11 | JN242516 | MBCSC:Fish:BH1122628 | 21.232 109.377 |
|  |  | *miiuy* | FSCS1008-11 | JN242515 | MBCSC:Fish:BH1122626 | 21.232 109.377 |
|  |  | *miiuy* | FSCS1007-11 | JN242514 | MBCSC:Fish:LCG1141825 | 23.5 117.666 |
|  |  | *miiuy* | FSCS1006-11 | JN242513 | MBCSC:Fish:LCG1141894 | 23.5 117.666 |
|  |  | *miiuy* | FSCS1005-11 | JN242512 | MBCSC:Fish:ZP1141256 | 21.333 111.977 |
|  |  | *miiuy* | FSCS1004-11 | JN242511 | MBCSC:Fish:ZH11518153 | 21.125 113.42 |
|  |  | *miiuy* | FSCS1003-11 | JN242510 | MBCSC:Fish:ZH11518151 | 21.125 113.42 |
|  |  | *miiuy* | FSCS1002-11 | JN242509 | MBCSC:Fish:ZP1141244 | 21.333 111.977 |
|  | *Nibea* | *albiflora* | FSCS412-07 | EU595222 | MBCSC:Fish:ZC I07065 | 20.516 110.981 |
|  |  | *albiflora* | FSCS1032-11 | JN242532 | MBCSC:Fish:ZH11518125 | 21.125 113.42 |
|  |  | *albiflora* | FSCS1031-11 | JN242531 | MBCSC:Fish:LCG116204 | 23.388 117.449 |
|  |  | *albiflora* | FSCS1030-11 | JN242530 | MBCSC:Fish:XS116938 | 17.311 113.276 |
|  |  | *albiflora* | FSCS1029-11 | JN242529 | MBCSC:Fish:LCG116320 | 23.125 117.876 |
|  |  | *albiflora* | FSCS1028-11 | JN242528 | MBCSC:Fish:BH1122678 | 21.232 109.377 |
|  | *Otolithes* | *ruber* | FSCS252-06 | EF607451 | MBCSC:Fish:GD 9083013 | 21.3 112.191 |
|  |  | *ruber* | FSCS306-06 | EF607450 | MBCSC:Fish:GD 9087017 | 21.435 111.128 |
|  | *Pennahia* | *anea* | FSCS291-06 | EF607488 | MBCSC:Fish:GD 9087002 | 21.47 111.239 |
|  |  | *anea* | FSCS313-06 | EF607487 | MBCSC:Fish:GD 9088001 | 22.678 114.549 |
|  | *Protonibea* | *diacanthus* | FSCS505-07 | EU595220 | MBCSC:Fish:ZC I07164 | 21.091 112.553 |
|  |  | *diacanthus* | FSCS813-08 | FJ238008 | MBCSC:Fish:ZC I07388 | 19.685 112.767 |
| Scomberesocidae | *Cololabis* | *saira* | FSCS423-07 | EU595081 | MBCSC:Fish:ZC I07082 | 20.412 108.333 |
|  |  | *saira* | FSCS424-07 | EU595080 | MBCSC:Fish:ZC I07083 | 20.567 118.022 |
|  |  | *saira* | FSCS425-07 | EU595079 | MBCSC:Fish:ZC I07084 | 19.366 113.422 |
|  |  | *saira* | FSCS426-07 | EU595078 | MBCSC:Fish:ZC I07085 | 20.443 111.037 |
|  |  | *saira* | FSCS427-07 | EU595077 | MBCSC:Fish:ZC I07086 | 19.685 112.767 |
|  |  | *saira* | FSCS428-07 | EU595076 | MBCSC:Fish:ZC I07087 | 19.685 108.5 |
|  |  | *sp.* | FSCS895-11 | JN242622 | MBCSC:Fish:TCL116604 | 21.216 110.956 |
|  |  | *sp.* | FSCS894-11 | JN242621 | MBCSC:Fish:TCL116603 | 21.216 110.956 |
| Scombridae | *Rastrelliger* | *kanagurta* | FSCS089-06 | EF607526 | MBCSC:Fish:GD 9086064 | 20.476 109.825 |
|  |  | *kanagurta* | FSCS090-06 | EF607525 | MBCSC:Fish:GD 9086065 | 20.476 109.825 |
|  |  | *kanagurta* | FSCS091-06 | EF607524 | MBCSC:Fish:GD 9086066 | 20.313 109.858 |
|  |  | *kanagurta* | FSCS092-06 | EF607523 | MBCSC:Fish:GD 9086067 | 20.476 109.825 |
|  |  | *kanagurta* | FSCS093-06 | EF607536 | MBCSC:Fish:GD 9086068 | 20.416 109.841 |
|  |  | *kanagurta* | FSCS180-06 | EF607522 | MBCSC:Fish:GD 9081028 | 20.767 110.675 |
|  |  | *kanagurta* | FSCS181-06 | EF607521 | MBCSC:Fish:GD 9081029 | 20.808 110.6 |
|  |  | *kanagurta* | FSCS309-06 | EF607520 | MBCSC:Fish:GD 9084002 | 22.678 114.549 |
|  | *Scomber* | *australasicus* | FSCS554-07 | EU595287 | MBCSC:Fish:ZC I07223 | 19.685 112.767 |
|  |  | *australasicus* | FSCS555-07 | EU595286 | MBCSC:Fish:ZC I07224 | 19.685 112.767 |
|  |  | *australasicus* | FSCS556-07 | EU595285 | MBCSC:Fish:ZC I07225 | 19.685 112.767 |
|  |  | *australasicus* | FSCS557-07 | EU595284 | MBCSC:Fish:ZC I07226 | 19.685 112.767 |
|  |  | *australasicus* | FSCS558-07 | EU595283 | MBCSC:Fish:ZC I07227 | 19.685 112.767 |
|  |  | *australasicus* | FSCS773-08 | FJ238035 | MBCSC:Fish:ZC I07348 | 20.875 111.21 |
|  |  | *australasicus* | FSCS774-08 | FJ238034 | MBCSC:Fish:ZC I07349 | 20.65 111.487 |
|  |  | *australasicus* | FSCS775-08 | FJ238033 | MBCSC:Fish:ZC I07350 | 20.65 111.487 |
|  |  | *japonicus* | FSCS1097-11 | JN242700 | MBCSC:Fish:XS116933 | 17.311 113.276 |
|  |  | *japonicus* | FSCS1096-11 | JN242699 | MBCSC:Fish:XS116932 | 17.311 113.276 |
|  |  | *japonicus* | FSCS1095-11 | JN242698 | MBCSC:Fish:ZH1151829 | 21.125 113.42 |
|  |  | *japonicus* | FSCS1094-11 | JN242697 | MBCSC:Fish:LCG1163134 | 23.125 117.876 |
|  |  | *japonicus* | FSCS1093-11 | JN242696 | MBCSC:Fish:ZP1141242 | 21.333 111.977 |
|  |  | *japonicus* | FSCS1092-11 | JN242695 | MBCSC:Fish:ZH1151870 | 21.125 113.42 |
|  |  | *japonicus* | FSCS1091-11 | JN242694 | MBCSC:Fish:LCG116210 | 23.388 117.449 |
|  |  | *japonicus* | FSCS1090-11 | JN242693 | MBCSC:Fish:BH1122606 | 21.232 109.377 |
|  |  | *japonicus* | FSCS1089-11 | JN242692 | MBCSC:Fish:BH1122604 | 21.232 109.377 |
|  | *Scomberomorus* | *commerson* | FSCS248-06 | EF607532 | MBCSC:Fish:GD 9083009 | 21.551 111.987 |
|  |  | *guttatus* | FSCS228-06 | EF607533 | MBCSC:Fish:GD 9081076 | 20.917 110.542 |
|  |  | *guttatus* | FSCS249-06 | EF607535 | MBCSC:Fish:GD 9083010 | 21.551 111.987 |
|  |  | *guttatus* | FSCS298-06 | EF607534 | MBCSC:Fish:GD 9087009 | 21.435 111.128 |
|  |  | *niphonius* | FSCS503-07 | EU595289 | MBCSC:Fish:ZC I07162 | 21.091 112.553 |
|  |  | *niphonius* | FSCS504-07 | EU595288 | MBCSC:Fish:ZC I07163 | 19.685 112.767 |
|  |  | *niphonius* | FSCS812-08 | FJ238036 | MBCSC:Fish:ZC I07387 | 20.55 111.2 |
|  |  | *niphonius* | FSCS1103-11 | JN242686 | MBCSC:Fish:ZP11412117 | 21.333 111.977 |
|  |  | *niphonius* | FSCS1102-11 | JN242687 | MBCSC:Fish:ZH1151810 | 21.125 113.42 |
|  |  | *niphonius* | FSCS1101-11 | JN242688 | MBCSC:Fish:XS116982 | 17.311 113.276 |
|  |  | *niphonius* | FSCS1100-11 | JN242689 | MBCSC:Fish:XS1169105 | 17.311 113.276 |
|  |  | *niphonius* | FSCS1099-11 | JN242690 | MBCSC:Fish:ZP1141215 | 21.333 111.977 |
|  |  | *niphonius* | FSCS1098-11 | JN242691 | MBCSC:Fish:ZP1141214 | 21.333 111.977 |
|  | *Thunnus* | *tonggol* | CFCS005-08 | FJ237960 | MBCSC:Fish:HN SY08321 | 16.035 114.134 |
|  |  | *tonggol* | CFCS134-08 | FJ237959 | MBCSC:Fish:HN SY08450 | 16.378 112.452 |
|  |  | *tonggol* | CFCS135-08 | FJ237958 | MBCSC:Fish:HN SY08451 | 16.378 112.452 |
|  |  | *tonggol* | CFCS136-08 | FJ237957 | MBCSC:Fish:HN SY08452 | 16.867 111.36 |
| Scorpaenidae | *Pterois* | *antennata* | CFCS089-08 | FJ237896 | MBCSC:Fish:HN SY08405 | 17.788 109.024 |
|  |  | *russelii* | CFCS092-08 | FJ237911 | MBCSC:Fish:HN SY08408 | 17.788 109.024 |
|  |  | *russelii* | CFCS093-08 | FJ237910 | MBCSC:Fish:HN SY08409 | 16.677 112.898 |
|  |  | *russelii* | CFCS094-08 | FJ237909 | MBCSC:Fish:HN SY08410 | 18.234 109.455 |
|  |  | *russelii* | CFCS095-08 | FJ237908 | MBCSC:Fish:HN SY08411 | 16.583 112.756 |
|  |  | *russelii* | CFCS096-08 | FJ237907 | MBCSC:Fish:HN SY08412 | 18.234 109.455 |
|  |  | *russelii* | CFCS097-08 | FJ237906 | MBCSC:Fish:HN SY08413 | 17.788 109.024 |
|  |  | *russelii* | CFCS128-08 | FJ237905 | MBCSC:Fish:HN SY08444 | 17.073 109.736 |
|  |  | *russelii* | CFCS129-08 | FJ237904 | MBCSC:Fish:HN SY08445 | 17.455 109.833 |
|  |  | *russelii* | CFCS213-08 | FJ237903 | MBCSC:Fish:HN SY08529 | 17.073 109.736 |
|  |  | *russelii* | CFCS217-08 | FJ237902 | MBCSC:Fish:HN SY08533 | 18.234 109.455 |
|  |  | *russelii* | CFCS355-08 | FJ237901 | MBCSC:Fish:HN SY08671 | 16.583 112.756 |
|  |  | *russelii* | CFCS356-08 | FJ237900 | MBCSC:Fish:HN SY08672 | 18.237 108.161 |
|  |  | *russelii* | CFCS357-08 | FJ237899 | MBCSC:Fish:HN SY08673 | 18.237 108.161 |
|  |  | *russelii* | CFCS358-08 | FJ237898 | MBCSC:Fish:HN SY08674 | 18.234 109.455 |
|  |  | *russelii* | CFCS359-08 | FJ237897 | MBCSC:Fish:HN SY08675 | 18.234 109.455 |
|  | *Scorpaenopsis* | *venosa* | FSCS1112-11 | JN242584 | MBCSC:Fish:ZH1151887 | 21.125 113.42 |
|  |  | *venosa* | FSCS1111-11 | JN242583 | MBCSC:Fish:ZH11518118 | 21.125 113.42 |
|  |  | *venosa* | FSCS1110-11 | JN242582 | MBCSC:Fish:ZP1141253 | 21.333 111.977 |
|  |  | *venosa* | FSCS1109-11 | JN242581 | MBCSC:Fish:ZH11518111 | 21.125 113.42 |
|  |  | *venosa* | FSCS1108-11 | JN242580 | MBCSC:Fish:BH1122608 | 21.232 109.377 |
|  |  | *venosa* | FSCS1107-11 | JN242579 | MBCSC:Fish:TCL116430 | 21.121 110.921 |
|  |  | *venosa* | FSCS1106-11 | JN242578 | MBCSC:Fish:TCL116429 | 21.121 110.921 |
|  |  | *venosa* | FSCS1105-11 | JN242577 | MBCSC:Fish:ZH11518109 | 21.125 113.42 |
|  |  | *venosa* | FSCS1104-11 | JN242576 | MBCSC:Fish:ZP1141229 | 21.333 111.977 |
|  |  | *vittapinna* | FSCS224-06 | EF607539 | MBCSC:Fish:GD 9081072 | 20.454 110.717 |
|  |  | *vittapinna* | FSCS225-06 | EF607538 | MBCSC:Fish:GD 9081073 | 20.917 110.542 |
|  |  | *vittapinna* | FSCS226-06 | EF607537 | MBCSC:Fish:GD 9081074 | 20.454 110.717 |
|  | *Parascorpaena* | *picta* | FSCS609-07 | EU595225 | MBCSC:Fish:ZC I07281 | 19.685 112.767 |
|  | *Dendrochirus* | *zebra* | CFCS090-08 | FJ237725 | MBCSC:Fish:HN SY08406 | 16.035 114.134 |
|  |  | *zebra* | CFCS091-08 | FJ237724 | MBCSC:Fish:HN SY08407 | 16.035 114.134 |
|  |  | *zebra* | CFCS349-08 | FJ237723 | MBCSC:Fish:HN SY08665 | 16.583 112.756 |
|  |  | *zebra* | CFCS350-08 | FJ237722 | MBCSC:Fish:HN SY08666 | 16.867 111.36 |
|  |  | *zebra* | CFCS351-08 | FJ237721 | MBCSC:Fish:HN SY08667 | 16.867 111.36 |
|  |  | *zebra* | CFCS352-08 | FJ237720 | MBCSC:Fish:HN SY08668 | 16.583 112.756 |
|  |  | *zebra* | CFCS353-08 | FJ237719 | MBCSC:Fish:HN SY08669 | 16.583 112.756 |
|  |  | *zebra* | CFCS354-08 | FJ237718 | MBCSC:Fish:HN SY08670 | 16.867 111.36 |
| Sebastidae | *Sebastiscus* | *marmoratus* | FSCS507-07 | EU595294 | MBCSC:Fish:ZC I07177 | 20.045 111.484 |
|  |  | *marmoratus* | FSCS508-07 | EU595293 | MBCSC:Fish:ZC I07178 | 20.045 111.484 |
|  |  | *marmoratus* | FSCS509-07 | EU595292 | MBCSC:Fish:ZC I07179 | 21.007 111.223 |
|  |  | *marmoratus* | FSCS510-07 | EU595291 | MBCSC:Fish:ZC I07275 | 21.007 111.223 |
|  |  | *marmoratus* | FSCS511-07 | EU595290 | MBCSC:Fish:ZC I07280 | 21.007 111.223 |
|  |  | *marmoratus* | FSCS825-08 | FJ238037 | MBCSC:Fish:ZC I07400 | 20.65 111.487 |
|  |  | *marmoratus* | FSCS1118-11 | JN242676 | MBCSC:Fish:XS116947 | 17.311 113.276 |
|  |  | *marmoratus* | FSCS1117-11 | JN242675 | MBCSC:Fish:XS116945 | 17.311 113.276 |
|  |  | *marmoratus* | FSCS1116-11 | JN242674 | MBCSC:Fish:ZH1151832 | 21.125 113.42 |
|  |  | *marmoratus* | FSCS1115-11 | JN242673 | MBCSC:Fish:BH1122612 | 21.232 109.377 |
|  |  | *marmoratus* | FSCS1114-11 | JN242672 | MBCSC:Fish:BH1122635 | 21.232 109.377 |
|  |  | *marmoratus* | FSCS1113-11 | JN242671 | MBCSC:Fish:BH1122634 | 21.232 109.377 |
| Serranidae | *Cephalopholis* | *boenak* | CFCS049-08 | FJ237680 | MBCSC:Fish:HN SY08365 | 16.378 112.452 |
|  |  | *boenak* | CFCS050-08 | FJ237679 | MBCSC:Fish:HN SY08366 | 16.378 112.452 |
|  |  | *boenak* | CFCS279-08 | FJ237678 | MBCSC:Fish:HN SY08595 | 16.583 112.756 |
|  |  | *boenak* | CFCS280-08 | FJ237677 | MBCSC:Fish:HN SY08596 | 16.867 111.36 |
|  |  | *boenak* | CFCS281-08 | FJ237676 | MBCSC:Fish:HN SY08597 | 16.583 112.756 |
|  |  | *boenak* | CFCS282-08 | FJ237675 | MBCSC:Fish:HN SY08598 | 16.583 112.756 |
|  |  | *boenak* | CFCS283-08 | FJ237674 | MBCSC:Fish:HN SY08599 | 16.583 112.756 |
|  | *Epinephelus* | *amblycephalus* | CFCS009-08 | FJ237754 | MBCSC:Fish:HN SY08325 | 16.677 112.898 |
|  |  | *amblycephalus* | CFCS010-08 | FJ237753 | MBCSC:Fish:HN SY08326 | 16.583 112.756 |
|  |  | *amblycephalus* | CFCS058-08 | FJ237752 | MBCSC:Fish:HN SY08374 | 16.035 114.134 |
|  |  | *amblycephalus* | CFCS059-08 | FJ237751 | MBCSC:Fish:HN SY08375 | 16.035 114.134 |
|  |  | *amblycephalus* | CFCS060-08 | FJ237750 | MBCSC:Fish:HN SY08376 | 16.035 114.134 |
|  |  | *amblycephalus* | CFCS061-08 | FJ237749 | MBCSC:Fish:HN SY08377 | 16.035 114.134 |
|  |  | *amblycephalus* | CFCS062-08 | FJ237748 | MBCSC:Fish:HN SY08378 | 16.035 114.134 |
|  |  | *amblycephalus* | CFCS063-08 | FJ237747 | MBCSC:Fish:HN SY08379 | 16.035 114.134 |
|  |  | *amblycephalus* | CFCS064-08 | FJ237746 | MBCSC:Fish:HN SY08380 | 16.378 112.452 |
|  |  | *amblycephalus* | CFCS152-08 | FJ237745 | MBCSC:Fish:HN SY08468 | 17.455 109.833 |
|  |  | *amblycephalus* | CFCS153-08 | FJ237744 | MBCSC:Fish:HN SY08469 | 17.073 109.736 |
|  |  | *amblycephalus* | CFCS154-08 | FJ237743 | MBCSC:Fish:HN SY08470 | 17.073 109.736 |
|  |  | *amblycephalus* | CFCS159-08 | FJ237742 | MBCSC:Fish:HN SY08475 | 17.073 109.736 |
|  |  | *amblycephalus* | CFCS160-08 | FJ237741 | MBCSC:Fish:HN SY08476 | 17.073 109.736 |
|  |  | *amblycephalus* | CFCS288-08 | FJ237740 | MBCSC:Fish:HN SY08604 | 16.583 112.756 |
|  |  | *amblycephalus* | CFCS289-08 | FJ237739 | MBCSC:Fish:HN SY08605 | 17.788 109.024 |
|  |  | *amblycephalus* | CFCS290-08 | FJ237738 | MBCSC:Fish:HN SY08606 | 17.788 109.024 |
|  |  | *amblycephalus* | CFCS291-08 | FJ237737 | MBCSC:Fish:HN SY08607 | 18.237 108.161 |
|  |  | *amblycephalus* | CFCS292-08 | FJ237736 | MBCSC:Fish:HN SY08608 | 18.237 108.161 |
|  |  | *amblycephalus* | CFCS293-08 | FJ237735 | MBCSC:Fish:HN SY08609 | 18.237 108.161 |
|  |  | *amblycephalus* | CFCS294-08 | FJ237734 | MBCSC:Fish:HN SY08610 | 18.237 108.161 |
|  |  | *amblycephalus* | CFCS295-08 | FJ237733 | MBCSC:Fish:HN SY08611 | 18.237 108.161 |
|  |  | *amblycephalus* | CFCS296-08 | FJ237732 | MBCSC:Fish:HN SY08612 | 18.237 108.161 |
|  |  | *amblycephalus* | CFCS297-08 | FJ237731 | MBCSC:Fish:HN SY08613 | 18.237 108.161 |
|  |  | *amblycephalus* | CFCS298-08 | FJ237730 | MBCSC:Fish:HN SY08614 | 18.237 108.161 |
|  |  | *amblycephalus* | CFCS299-08 | FJ237729 | MBCSC:Fish:HN SY08615 | 18.237 108.161 |
|  |  | *amblycephalus* | CFCS300-08 | FJ237728 | MBCSC:Fish:HN SY08616 | 18.237 108.161 |
|  |  | *amblycephalus* | CFCS301-08 | FJ237727 | MBCSC:Fish:HN SY08617 | 17.788 109.024 |
|  |  | *amblycephalus* | CFCS302-08 | FJ237726 | MBCSC:Fish:HN SY08618 | 17.788 109.024 |
|  |  | *amblycephalus* | FSCS916-11 | JN242614 | MBCSC:Fish:ZH11518104 | 21.125 113.42 |
|  |  | *amblycephalus* | FSCS915-11 | JN242613 | MBCSC:Fish:BH1122617 | 21.232 109.377 |
|  |  | *amblycephalus* | FSCS914-11 | JN242612 | MBCSC:Fish:ZH11518115 | 21.125 113.42 |
|  |  | *amblycephalus* | FSCS913-11 | JN242611 | MBCSC:Fish:ZP1141236 | 21.333 111.977 |
|  |  | *amblycephalus* | FSCS912-11 | JN242610 | MBCSC:Fish:XS1169116 | 17.311 113.276 |
|  |  | *amblycephalus* | FSCS911-11 | JN242609 | MBCSC:Fish:TCL116418 | 21.121 110.921 |
|  |  | *amblycephalus* | FSCS910-11 | JN242608 | MBCSC:Fish:ZH1151802 | 21.125 113.42 |
|  |  | *areolatus* | CFCS053-08 | FJ237763 | MBCSC:Fish:HN SY08369 | 18.216 109.447 |
|  |  | *areolatus* | CFCS054-08 | FJ237762 | MBCSC:Fish:HN SY08370 | 16.035 114.134 |
|  |  | *areolatus* | CFCS055-08 | FJ237761 | MBCSC:Fish:HN SY08371 | 16.035 114.134 |
|  |  | *areolatus* | CFCS056-08 | FJ237760 | MBCSC:Fish:HN SY08372 | 16.035 114.134 |
|  |  | *areolatus* | CFCS057-08 | FJ237759 | MBCSC:Fish:HN SY08373 | 16.035 114.134 |
|  |  | *areolatus* | CFCS284-08 | FJ237758 | MBCSC:Fish:HN SY08600 | 16.583 112.756 |
|  |  | *areolatus* | CFCS285-08 | FJ237757 | MBCSC:Fish:HN SY08601 | 16.867 111.36 |
|  |  | *areolatus* | CFCS286-08 | FJ237756 | MBCSC:Fish:HN SY08602 | 16.583 112.756 |
|  |  | *areolatus* | CFCS287-08 | FJ237755 | MBCSC:Fish:HN SY08603 | 16.867 111.36 |
|  |  | *sp.* | FSCS418-07 | EU595115 | MBCSC:Fish:ZC I07077 | 20.516 110.981 |
|  |  | *sp.* | FSCS419-07 | EU595114 | MBCSC:Fish:ZC I07078 | 21.007 111.223 |
|  |  | *sp.* | FSCS420-07 | EU595113 | MBCSC:Fish:ZC I07079 | 21.007 111.223 |
|  |  | *sp.* | FSCS421-07 | EU595112 | MBCSC:Fish:ZC I07080 | 17.235 112.811 |
|  |  | *sp.* | FSCS422-07 | EU595111 | MBCSC:Fish:ZC I07081 | 21.074 111.557 |
|  |  | *bleekeri* | FSCS445-07 | EU595110 | MBCSC:Fish:ZC I07106 | 19.685 112.767 |
|  |  | *bleekeri* | CFCS051-08 | FJ237767 | MBCSC:Fish:HN SY08367 | 16.378 112.452 |
|  |  | *bleekeri* | CFCS052-08 | FJ237766 | MBCSC:Fish:HN SY08368 | 16.378 112.452 |
|  |  | *bleekeri* | CFCS098-08 | FJ237765 | MBCSC:Fish:HN SY08414 | 16.583 112.756 |
|  |  | *bleekeri* | CFCS130-08 | FJ237764 | MBCSC:Fish:HN SY08446 | 16.378 112.452 |
|  |  | *bleekeri* | FSCS922-11 | JN242646 | MBCSC:Fish:BH1122648 | 21.232 109.377 |
|  |  | *bleekeri* | FSCS921-11 | JN242645 | MBCSC:Fish:BH1122647 | 21.232 109.377 |
|  |  | *bleekeri* | FSCS920-11 | JN242644 | MBCSC:Fish:ZH11518119 | 21.125 113.42 |
|  |  | *bleekeri* | FSCS919-11 | JN242643 | MBCSC:Fish:TCL116419 | 21.121 110.921 |
|  |  | *bleekeri* | FSCS918-11 | JN242642 | MBCSC:Fish:XS116961 | 17.311 113.276 |
|  |  | *bleekeri* | FSCS917-11 | JN242641 | MBCSC:Fish:XS116959 | 17.311 113.276 |
|  |  | *coioides* | FSCS930-11 | JN242468 | MBCSC:Fish:XS1169135 | 17.311 113.276 |
|  |  | *coioides* | FSCS929-11 | JN242467 | MBCSC:Fish:BH1122621 | 21.232 109.377 |
|  |  | *coioides* | FSCS928-11 | JN242466 | MBCSC:Fish:XS116990 | 17.311 113.276 |
|  |  | *coioides* | FSCS927-11 | JN242465 | MBCSC:Fish:XS116991 | 17.311 113.276 |
|  |  | *coioides* | FSCS926-11 | JN242464 | MBCSC:Fish:BH1122613 | 21.232 109.377 |
|  |  | *coioides* | FSCS925-11 | JN242463 | MBCSC:Fish:ZH1151859 | 21.125 113.42 |
|  |  | *coioides* | FSCS924-11 | JN242462 | MBCSC:Fish:LCG116213 | 23.388 117.449 |
|  |  | *epistictus* | CFCS146-08 | FJ237768 | MBCSC:Fish:HN SY08462 | 17.073 109.736 |
|  |  | *fasciatomaculosus* | FSCS099-06 | EF607565 | MBCSC:Fish:GD 9085029 | 20.476 109.825 |
|  |  | *maculatus* | FSCS936-11 | JN242490 | MBCSC:Fish:BH1122643 | 21.232 109.377 |
|  |  | *maculatus* | FSCS935-11 | JN242489 | MBCSC:Fish:BH1122642 | 21.232 109.377 |
|  |  | *maculatus* | FSCS934-11 | JN242488 | MBCSC:Fish:LCG116298 | 23.388 117.449 |
|  |  | *maculatus* | FSCS933-11 | JN242487 | MBCSC:Fish:ZH11518132 | 21.125 113.42 |
|  |  | *maculatus* | FSCS932-11 | JN242486 | MBCSC:Fish:ZH1151895 | 21.125 113.42 |
|  |  | *maculatus* | FSCS931-11 | JN242485 | MBCSC:Fish:LCG116296 | 23.388 117.449 |
|  |  | *poecilonotus* | CFCS065-08 | FJ237769 | MBCSC:Fish:HN SY08381 | 17.788 109.024 |
|  |  | *sexfasciatus* | FSCS182-06 | EF607564 | MBCSC:Fish:GD 9081030 | 20.767 110.675 |
|  |  | *sexfasciatus* | FSCS344-07 | EU595123 | MBCSC:Fish:ZC I07014 | 21.007 111.223 |
|  |  | *sexfasciatus* | FSCS512-07 | EU595122 | MBCSC:Fish:ZC I07180 | 21.091 112.553 |
|  |  | *sexfasciatus* | FSCS603-07 | EU595121 | MBCSC:Fish:ZC I07273 | 16.336 111.017 |
|  |  | *sexfasciatus* | FSCS604-07 | EU595120 | MBCSC:Fish:ZC I07274 | 19.685 112.767 |
|  |  | *sexfasciatus* | FSCS605-07 | EU595119 | MBCSC:Fish:ZC I07276 | 19.685 112.767 |
|  |  | *sexfasciatus* | FSCS606-07 | EU595118 | MBCSC:Fish:ZC I07277 | 21.091 112.553 |
|  |  | *sexfasciatus* | FSCS607-07 | EU595117 | MBCSC:Fish:ZC I07278 | 21.091 112.553 |
|  |  | *sexfasciatus* | FSCS608-07 | EU595116 | MBCSC:Fish:ZC I07279 | 21.091 112.553 |
|  |  | *spilotoceps* | CFCS012-08 | FJ237776 | MBCSC:Fish:HN SY08328 | 17.073 109.736 |
|  |  | *spilotoceps* | CFCS022-08 | FJ237775 | MBCSC:Fish:HN SY08338 | 16.035 114.134 |
|  |  | *spilotoceps* | CFCS163-08 | FJ237774 | MBCSC:Fish:HN SY08479 | 17.455 109.833 |
|  |  | *spilotoceps* | CFCS164-08 | FJ237773 | MBCSC:Fish:HN SY08480 | 17.455 109.833 |
|  |  | *spilotoceps* | CFCS165-08 | FJ237772 | MBCSC:Fish:HN SY08481 | 17.455 109.833 |
|  |  | *spilotoceps* | CFCS205-08 | FJ237771 | MBCSC:Fish:HN SY08521 | 17.073 109.736 |
|  |  | *spilotoceps* | CFCS206-08 | FJ237770 | MBCSC:Fish:HN SY08522 | 17.073 109.736 |
|  | *Plectropomus* | *areolatus* | FSCS1066-11 | JN242595 | MBCSC:Fish:LCG116395 | 23.125 117.876 |
|  |  | *areolatus* | FSCS1065-11 | JN242594 | MBCSC:Fish:ZH1151868 | 21.125 113.42 |
|  |  | *areolatus* | FSCS1064-11 | JN242593 | MBCSC:Fish:ZH1151865 | 21.125 113.42 |
|  |  | *areolatus* | FSCS1063-11 | JN242592 | MBCSC:Fish:TCL116601 | 21.216 110.956 |
|  |  | *areolatus* | FSCS1062-11 | JN242591 | MBCSC:Fish:ZP1141219 | 21.333 111.977 |
|  |  | *leopardus* | FSCS615-07 | EU595233 | MBCSC:Fish:ZC I07287 | 16.643 113.336 |
| Siganidae | *Siganus* | *argenteus* | FSCS230-06 | EF607555 | MBCSC:Fish:GD 9081078 | 20.454 110.717 |
|  |  | *argenteus* | FSCS311-06 | EF607558 | MBCSC:Fish:GD 9084004 | 22.749 115.211 |
|  |  | *argenteus* | FSCS317-06 | EF607557 | MBCSC:Fish:GD 9088005 | 23.296 116.784 |
|  |  | *argenteus* | FSCS319-06 | EF607556 | MBCSC:Fish:GD 9088007 | 23.256 116.833 |
|  |  | *guttatus* | CFCS036-08 | FJ237944 | MBCSC:Fish:HN SY08352 | 18.234 109.455 |
|  |  | *guttatus* | CFCS037-08 | FJ237943 | MBCSC:Fish:HN SY08353 | 16.035 114.134 |
|  |  | *guttatus* | CFCS038-08 | FJ237942 | MBCSC:Fish:HN SY08354 | 18.216 109.447 |
|  |  | *guttatus* | CFCS039-08 | FJ237941 | MBCSC:Fish:HN SY08355 | 16.583 112.756 |
|  |  | *guttatus* | CFCS040-08 | FJ237940 | MBCSC:Fish:HN SY08356 | 16.035 114.134 |
|  |  | *guttatus* | CFCS041-08 | FJ237939 | MBCSC:Fish:HN SY08357 | 18.216 109.447 |
|  |  | *guttatus* | CFCS247-08 | FJ237938 | MBCSC:Fish:HN SY08563 | 17.788 109.024 |
|  |  | *guttatus* | CFCS248-08 | FJ237937 | MBCSC:Fish:HN SY08564 | 17.788 109.024 |
|  |  | *guttatus* | CFCS249-08 | FJ237936 | MBCSC:Fish:HN SY08565 | 17.788 109.024 |
|  |  | *guttatus* | CFCS250-08 | FJ237935 | MBCSC:Fish:HN SY08566 | 18.234 109.455 |
|  |  | *guttatus* | CFCS251-08 | FJ237934 | MBCSC:Fish:HN SY08567 | 18.234 109.455 |
|  |  | *guttatus* | CFCS252-08 | FJ237933 | MBCSC:Fish:HN SY08568 | 18.234 109.455 |
|  |  | *guttatus* | CFCS253-08 | FJ237932 | MBCSC:Fish:HN SY08569 | 18.234 109.455 |
|  |  | *guttatus* | CFCS254-08 | FJ237931 | MBCSC:Fish:HN SY08570 | 17.788 109.024 |
|  |  | *guttatus* | CFCS255-08 | FJ237930 | MBCSC:Fish:HN SY08571 | 17.788 109.024 |
|  |  | *guttatus* | CFCS256-08 | FJ237929 | MBCSC:Fish:HN SY08572 | 18.234 109.455 |
|  |  | *guttatus* | CFCS257-08 | FJ237928 | MBCSC:Fish:HN SY08573 | 18.234 109.455 |
| Sillaginidae | *Sillago* | *asiatica* | FSCS1130-11 | JN242607 | MBCSC:Fish:XS116948 | 17.311 113.276 |
|  |  | *asiatica* | FSCS1129-11 | JN242606 | MBCSC:Fish:ZP1141228 | 21.333 111.977 |
|  |  | *asiatica* | FSCS1128-11 | JN242605 | MBCSC:Fish:LCG116406 | 23.45 117.379 |
|  |  | *asiatica* | FSCS1127-11 | JN242604 | MBCSC:Fish:BH1122614 | 21.232 109.377 |
|  |  | *maculata* | FSCS081-06 | EF607559 | MBCSC:Fish:GD 9086056 | 20.476 109.825 |
|  |  | *maculata* | FSCS332-07 | EU595295 | MBCSC:Fish:ZC I07002 | 19.685 112.767 |
|  |  | *sihama* | FSCS196-06 | EF607563 | MBCSC:Fish:GD 9081044 | 20.917 110.542 |
|  |  | *sihama* | FSCS197-06 | EF607562 | MBCSC:Fish:GD 9081045 | 20.917 110.542 |
|  |  | *sihama* | FSCS246-06 | EF607561 | MBCSC:Fish:GD 9083007 | 21.551 111.987 |
|  |  | *sihama* | FSCS247-06 | EF607560 | MBCSC:Fish:GD 9083008 | 21.58 111.815 |
| Soleidae | *Brachirus* | *orientalis* | FSCS009-06 | EF607337 | MBCSC:Fish:GD 9085009 | 20.313 109.858 |
|  | *Pardachirus* | *pavoninus* | FSCS253-06 | EF607484 | MBCSC:Fish:GD 9083014 | 21.551 111.987 |
|  |  | *pavoninus* | FSCS255-06 | EF607483 | MBCSC:Fish:GD 9083016 | 21.551 111.987 |
|  | *Solea* | *ovata* | FSCS375-07 | EU595303 | MBCSC:Fish:ZC I07037 | 21.074 111.557 |
|  |  | *ovata* | FSCS376-07 | EU595302 | MBCSC:Fish:ZC I07038 | 21.007 111.223 |
|  |  | *ovata* | FSCS377-07 | EU595301 | MBCSC:Fish:ZC I07039 | 21.007 111.223 |
|  |  | *ovata* | FSCS378-07 | EU595300 | MBCSC:Fish:ZC I07034 | 19.366 113.422 |
|  |  | *ovata* | FSCS379-07 | EU595299 | MBCSC:Fish:ZC I07040 | 19.366 113.422 |
|  |  | *ovata* | FSCS380-07 | EU595298 | MBCSC:Fish:ZC I07041 | 19.366 113.422 |
|  |  | *ovata* | FSCS381-07 | EU595297 | MBCSC:Fish:ZC I07042 | 21.074 111.557 |
|  |  | *ovata* | FSCS382-07 | EU595296 | MBCSC:Fish:ZC I07043 | 21.074 111.557 |
|  | *Zebrias* | *quagga* | FSCS109-06 | EF607616 | MBCSC:Fish:GD 9085039 | 20.476 109.825 |
|  |  | *quagga* | FSCS513-07 | EU595351 | MBCSC:Fish:ZC I07182 | 20.516 110.981 |
|  |  | *quagga* | FSCS514-07 | EU595350 | MBCSC:Fish:ZC I07183 | 20.516 110.981 |
|  |  | *quagga* | FSCS515-07 | EU595349 | MBCSC:Fish:ZC I07184 | 20.516 110.981 |
|  |  | *quagga* | FSCS516-07 | EU595348 | MBCSC:Fish:ZC I07185 | 20.516 110.981 |
|  |  | *quagga* | FSCS517-07 | EU595347 | MBCSC:Fish:ZC I07186 | 20.516 110.981 |
|  |  | *quagga* | FSCS518-07 | EU595346 | MBCSC:Fish:ZC I07187 | 20.045 111.484 |
|  |  | *quagga* | FSCS519-07 | EU595345 | MBCSC:Fish:ZC I07188 | 21.074 111.557 |
|  |  | *quagga* | FSCS520-07 | EU595344 | MBCSC:Fish:ZC I07189 | 21.074 111.557 |
|  |  | *quagga* | FSCS828-08 | FJ238049 | MBCSC:Fish:ZC I07403 | 20.65 111.487 |
|  |  | *quagga* | FSCS830-08 | FJ238050 | MBCSC:Fish:ZC I07405 | 20.65 111.487 |
| Sparidae | *Acanthopagrus* | *berda* | FSCS078-06 | EF607297 | MBCSC:Fish:GD 9086053 | 20.313 109.858 |
|  |  | *schlegelii* | FSCS143-06 | EF607300 | MBCSC:Fish:GD 9085073 | 20.454 109.889 |
|  |  | *schlegelii* |  |  |  |  |
|  |  | *schlegelii schlegelii* | FSCS502-07 | EU595056 | MBCSC:Fish:ZC I07161 | 19.685 112.767 |
|  |  | *latus* | FSCS359-07 | EU595055 | MBCSC:Fish:ZC I07027 | 21.091 112.553 |
|  |  | *latus* | FSCS360-07 | EU595054 | MBCSC:Fish:ZC I07028 | 20.516 110.981 |
|  |  | *latus* | FSCS361-07 | EU595053 | MBCSC:Fish:ZC I07029 | 19.366 113.422 |
|  |  | *latus* | FSCS362-07 | EU595052 | MBCSC:Fish:ZC I07030 | 20.516 110.981 |
|  |  | *latus* | FSCS363-07 | EU595051 | MBCSC:Fish:ZC I07031 | 21.074 111.557 |
|  |  | *latus* | FSCS364-07 | EU595050 | MBCSC:Fish:ZC I07032 | 19.366 113.422 |
|  |  | *latus* | FSCS365-07 | EU595049 | MBCSC:Fish:ZC I07033 | 21.074 111.557 |
|  |  | *latus* | FSCS367-07 | EU595048 | MBCSC:Fish:ZC I07067 | 21.091 112.553 |
|  |  | *latus* | FSCS780-08 | FJ237966 | MBCSC:Fish:ZC I07355 | 20.875 111.21 |
|  |  | *latus* | FSCS782-08 | FJ237965 | MBCSC:Fish:ZC I07357 | 20.875 111.21 |
|  |  | *latus* | FSCS783-08 | FJ237964 | MBCSC:Fish:ZC I07358 | 20.875 111.21 |
|  |  | *latus* | FSCS843-11 | JN242741 | MBCSC:Fish:ZP1141235 | 21.333 111.977 |
|  |  | *latus* | FSCS842-11 | JN242740 | MBCSC:Fish:TCL116452 | 21.121 110.921 |
|  |  | *latus* | FSCS841-11 | JN242739 | MBCSC:Fish:BH1122660 | 21.232 109.377 |
|  |  | *latus* | FSCS840-11 | JN242738 | MBCSC:Fish:LCG1132502 | 23.666 118.05 |
|  |  | *schlegelii* | FSCS847-11 | JN242575 | MBCSC:Fish:XS1169120 | 17.311 113.276 |
|  |  | *schlegelii* | FSCS846-11 | JN242574 | MBCSC:Fish:XS116980 | 17.311 113.276 |
|  |  | *schlegelii* | FSCS845-11 | JN242573 | MBCSC:Fish:ZP1121245 | 21.086 111.81 |
|  |  | *schlegelii* | FSCS844-11 | JN242572 | MBCSC:Fish:TCL116417 | 21.121 110.921 |
|  | *Evynnis* | *cardinalis* | FSCS561-07 | EU595134 | MBCSC:Fish:ZC I07230 | 21.074 111.557 |
|  |  | *cardinalis* | FSCS562-07 | EU595133 | MBCSC:Fish:ZC I07231 | 21.074 111.557 |
|  |  | *cardinalis* | FSCS563-07 | EU595132 | MBCSC:Fish:ZC I07232 | 21.074 111.557 |
|  |  | *cardinalis* | FSCS564-07 | EU595131 | MBCSC:Fish:ZC I07233 | 20.497 113.568 |
|  |  | *cardinalis* | FSCS565-07 | EU595130 | MBCSC:Fish:ZC I07234 | 20.497 113.568 |
|  |  | *cardinalis* | FSCS566-07 | EU595129 | MBCSC:Fish:ZC I07235 | 20.497 113.568 |
|  |  | *cardinalis* | FSCS567-07 | EU595128 | MBCSC:Fish:ZC I07236 | 20.497 113.568 |
|  |  | *cardinalis* | FSCS786-08 | FJ237987 | MBCSC:Fish:ZC I07361 | 20.875 111.21 |
|  |  | *cardinalis* | FSCS789-08 | FJ237986 | MBCSC:Fish:ZC I07364 | 20.875 111.21 |
|  | *Pagrus* | *major* | CFCS066-08 | FJ237870 | MBCSC:Fish:HN SY08382 | 16.378 112.452 |
|  |  | *major* | CFCS067-08 | FJ237869 | MBCSC:Fish:HN SY08383 | 16.583 112.756 |
|  |  | *major* | CFCS303-08 | FJ237868 | MBCSC:Fish:HN SY08619 | 17.788 109.024 |
|  |  | *major* | CFCS304-08 | FJ237867 | MBCSC:Fish:HN SY08620 | 17.788 109.024 |
|  |  | *major* | CFCS305-08 | FJ237866 | MBCSC:Fish:HN SY08621 | 17.788 109.024 |
|  |  | *major* | CFCS306-08 | FJ237865 | MBCSC:Fish:HN SY08622 | 17.788 109.024 |
|  |  | *major* | CFCS307-08 | FJ237864 | MBCSC:Fish:HN SY08623 | 17.788 109.024 |
|  |  | *major* | CFCS308-08 | FJ237863 | MBCSC:Fish:HN SY08624 | 17.788 109.024 |
|  |  | *major* | CFCS309-08 | FJ237862 | MBCSC:Fish:HN SY08625 | 17.788 109.024 |
|  | *Rhabdosargus* | *sarba* | FSCS178-06 | EF607299 | MBCSC:Fish:GD 9081026 | 20.767 110.675 |
|  |  | *sarba* | FSCS308-06 | EF607298 | MBCSC:Fish:GD 9084001 | 22.749 115.211 |
|  |  | *sarba* | FSCS815-08 | FJ238020 | MBCSC:Fish:ZC I07390 | 20.65 111.487 |
| Sphyraenidae | *Sphyraena* | *pinguis* | FSCS098-06 | EF607371 | MBCSC:Fish:GD 9085028 | 20.454 109.889 |
|  |  | *putnamae* | FSCS586-07 | EU595309 | MBCSC:Fish:ZC I07256 | 21.007 111.223 |
|  |  | *putnamae* | FSCS587-07 | EU595308 | MBCSC:Fish:ZC I07257 | 21.007 111.223 |
|  |  | *putnamae* | FSCS588-07 | EU595307 | MBCSC:Fish:ZC I07258 | 21.007 111.223 |
|  |  | *putnamae* | FSCS589-07 | EU595306 | MBCSC:Fish:ZC I07259 | 20.045 111.484 |
|  |  | *putnamae* | FSCS590-07 | EU595305 | MBCSC:Fish:ZC I07260 | 20.045 111.484 |
|  |  | *putnamae* | FSCS591-07 | EU595304 | MBCSC:Fish:ZC I07261 | 20.045 111.484 |
|  |  | *putnamae* | FSCS811-08 | FJ238038 | MBCSC:Fish:ZC I07386 | 20.875 111.21 |
|  |  | *putnamae* | CFCS069-08 | FJ237950 | MBCSC:Fish:HN SY08385 | 16.378 112.452 |
|  |  | *putnamae* | CFCS070-08 | FJ237949 | MBCSC:Fish:HN SY08386 | 16.583 112.756 |
|  |  | *putnamae* | CFCS315-08 | FJ237948 | MBCSC:Fish:HN SY08631 | 17.788 109.024 |
|  |  | *putnamae* | CFCS316-08 | FJ237947 | MBCSC:Fish:HN SY08632 | 18.237 108.161 |
|  |  | *putnamae* | CFCS317-08 | FJ237946 | MBCSC:Fish:HN SY08633 | 17.788 109.024 |
|  |  | *putnamae* | CFCS318-08 | FJ237945 | MBCSC:Fish:HN SY08634 | 18.234 109.455 |
| Sphyrnidae | *Sphyrna* | *lewini* | CFCS002-08 | FJ237956 | MBCSC:Fish:HN SY08318 | 16.378 112.452 |
|  |  | *lewini* | CFCS003-08 | FJ237955 | MBCSC:Fish:HN SY08319 | 16.378 112.452 |
|  |  | *lewini* | CFCS004-08 | FJ237954 | MBCSC:Fish:HN SY08320 | 16.378 112.452 |
|  |  | *lewini* | CFCS126-08 | FJ237953 | MBCSC:Fish:HN SY08442 | 17.455 109.833 |
|  |  | *lewini* | CFCS127-08 | FJ237952 | MBCSC:Fish:HN SY08443 | 17.073 109.736 |
|  |  | *lewini* | CFCS131-08 | FJ237951 | MBCSC:Fish:HN SY08447 | 16.378 112.452 |
| Stromateidae | *Pampus* | *argenteus* | FSCS074-06 | EF607457 | MBCSC:Fish:GD 9086049 | 20.476 109.825 |
|  |  | *argenteus* | FSCS140-06 | EF607460 | MBCSC:Fish:GD 9085070 | 20.454 109.889 |
|  |  | *argenteus* | FSCS141-06 | EF607459 | MBCSC:Fish:GD 9085071 | 20.476 109.825 |
|  |  | *argenteus* | FSCS142-06 | EF607458 | MBCSC:Fish:GD 9085072 | 20.454 109.889 |
|  |  | *chinensis* | FSCS576-07 | EU595223 | MBCSC:Fish:ZC I07246 | 19.685 112.767 |
|  |  | *cinereus* | FSCS073-06 | EF607462 | MBCSC:Fish:GD 9086048 | 20.313 109.858 |
|  |  | *cinereus* | FSCS075-06 | EF607461 | MBCSC:Fish:GD 9086050 | 20.476 109.825 |
|  |  | *cinereus* | FSCS289-06 | EF607466 | MBCSC:Fish:GD 9082032 | 21.089 110.452 |
|  |  | *cinereus* | FSCS323-06 | EF607465 | MBCSC:Fish:GD 9088011 | 23.296 116.784 |
|  |  | *cinereus* | FSCS324-06 | EF607464 | MBCSC:Fish:GD 9088012 | 23.256 116.833 |
|  |  | *cinereus* | FSCS325-06 | EF607463 | MBCSC:Fish:GD 9088013 | 23.296 116.784 |
|  |  | *echinogaster* | FSCS1052-11 | JN242670 | MBCSC:Fish:LCG1163130 | 23.125 117.876 |
|  |  | *echinogaster* | FSCS1051-11 | JN242669 | MBCSC:Fish:ZP1141254 | 21.333 111.977 |
|  |  | *echinogaster* | FSCS1050-11 | JN242668 | MBCSC:Fish:LCG1163129 | 23.125 117.876 |
|  |  | *echinogaster* | FSCS1049-11 | JN242667 | MBCSC:Fish:BH1122625 | 21.232 109.377 |
|  |  | *echinogaster* | FSCS1048-11 | JN242666 | MBCSC:Fish:LCG116321 | 23.125 117.876 |
|  |  | *echinogaster* | FSCS1047-11 | JN242665 | MBCSC:Fish:ZP1141261 | 21.333 111.977 |
|  |  | *Nozawae* | FSCS577-07 | EU595224 | MBCSC:Fish:ZC I07247 | 19.685 112.767 |
|  |  | *punctatissimus* | FSCS1061-11 | JN242737 | MBCSC:Fish:ZP1141218 | 21.333 111.977 |
|  |  | *punctatissimus* | FSCS1060-11 | JN242736 | MBCSC:Fish:ZP1141243 | 21.333 111.977 |
|  |  | *punctatissimus* | FSCS1059-11 | JN242735 | MBCSC:Fish:ZH1151885 | 21.125 113.42 |
|  |  | *punctatissimus* | FSCS1058-11 | JN242734 | MBCSC:Fish:LCG116381 | 23.125 117.876 |
|  |  | *punctatissimus* | FSCS1057-11 | JN242733 | MBCSC:Fish:BH1122633 | 21.232 109.377 |
|  |  | *punctatissimus* | FSCS1056-11 | JN242732 | MBCSC:Fish:BH1122632 | 21.232 109.377 |
|  |  | *punctatissimus* | FSCS1055-11 | JN242731 | MBCSC:Fish:BH1122631 | 21.232 109.377 |
|  |  | *punctatissimus* | FSCS1054-11 | JN242730 | MBCSC:Fish:LCG1132513 | 23.666 118.05 |
|  |  | *punctatissimus* | FSCS1053-11 | JN242729 | MBCSC:Fish:ZH1151838 | 21.125 113.42 |
| Synanceiidae | *Minous* | *monodactylus* | FSCS390-07 | EU595221 | MBCSC:Fish:ZC I07051 | 21.074 111.557 |
|  | *Synanceia* | *sp.* | FSCS131-06 | EF607409 | MBCSC:Fish:GD 9085061 | 20.476 109.825 |
| Synodontidae | *Harpadon* | *nehereus* | FSCS955-11 | JN242630 | MBCSC:Fish:BH11226103 | 21.232 109.377 |
|  |  | *nehereus* | FSCS954-11 | JN242631 | MBCSC:Fish:BH1122611 | 21.232 109.377 |
|  |  | *nehereus* | FSCS953-11 | JN242632 | MBCSC:Fish:ZH1151875 | 21.125 113.42 |
|  |  | *nehereus* | FSCS952-11 | JN242634 | MBCSC:Fish:TCL1164133 | 21.121 110.921 |
|  |  | *nehereus* | FSCS951-11 | JN242633 | MBCSC:Fish:BH1122630 | 21.232 109.377 |
|  |  | *nehereus* | FSCS950-11 | JN242635 | MBCSC:Fish:BH1122629 | 21.232 109.377 |
|  |  | *nehereus* | FSCS949-11 | JN242636 | MBCSC:Fish:ZH1151872 | 21.125 113.42 |
|  |  | *nehereus* | FSCS948-11 | JN242637 | MBCSC:Fish:TCL1164112 | 21.121 110.921 |
|  |  | *nehereus* | FSCS947-11 | JN242638 | MBCSC:Fish:LCG116407 | 23.45 117.379 |
|  |  | *nehereus* | FSCS946-11 | JN242639 | MBCSC:Fish:TCL1164107 | 21.121 110.921 |
|  |  | *nehereus* | FSCS945-11 | JN242640 | MBCSC:Fish:XS116988 | 17.311 113.276 |
|  |  | *nehereus* | FSCS307-06 | EF607399 | MBCSC:Fish:GD 9087018 | 21.435 111.128 |
|  |  | *nehereus* | FSCS326-06 | EF607398 | MBCSC:Fish:GD 9088014 | 23.296 116.784 |
|  | *Saurida* | *elongata* | FSCS100-06 | EF607514 | MBCSC:Fish:GD 9085030 | 20.454 109.889 |
|  |  | *elongata* | FSCS355-07 | EU595270 | MBCSC:Fish:ZC I07023 | 21.091 112.553 |
|  |  | *elongata* | FSCS356-07 | EU595269 | MBCSC:Fish:ZC I07024 | 19.366 113.422 |
|  |  | *elongata* | FSCS357-07 | EU595268 | MBCSC:Fish:ZC I07025 | 19.366 113.422 |
|  |  | *elongata* | FSCS358-07 | EU595267 | MBCSC:Fish:ZC I07026 | 19.685 112.767 |
|  |  | *elongata* | FSCS766-08 | FJ238024 | MBCSC:Fish:ZC I07341 | 20.875 111.21 |
|  |  | *sp.* | FSCS277-06 | EF607515 | MBCSC:Fish:GD 9082020 | 21.047 110.665 |
|  | *Trachinocephalus* | *myops* | FSCS495-07 | EU595324 | MBCSC:Fish:ZC I07154 | 20.045 111.484 |
|  |  | *myops* | FSCS496-07 | EU595323 | MBCSC:Fish:ZC I07155 | 20.045 111.484 |
| Tetraodontidae | *Takifugu* | *oblongus* | FSCS094-06 | EF607572 | MBCSC:Fish:GD 9086069 | 20.476 109.825 |
|  |  | *oblongus* | FSCS614-07 | EU595321 | MBCSC:Fish:ZC I07286 | 20.516 110.981 |
|  |  | *oblongus* | FSCS827-08 | FJ238042 | MBCSC:Fish:ZC I07402 | 20.65 111.487 |
|  | *Lagocephalus* | *spadiceus* | FSCS302-06 | EF607419 | MBCSC:Fish:GD 9087013 | 21.435 111.128 |
|  |  | *spadiceus* | FSCS610-07 | EU595163 | MBCSC:Fish:ZC I07282 | 21.007 111.223 |
|  |  | *spadiceus* | FSCS611-07 | EU595162 | MBCSC:Fish:ZC I07283 | 20.516 110.981 |
|  |  | *spadiceus* | FSCS612-07 | EU595161 | MBCSC:Fish:ZC I07284 | 20.516 110.981 |
|  |  | *spadiceus* | FSCS613-07 | EU595160 | MBCSC:Fish:ZC I07285 | 20.516 110.981 |
| Terapontidae | *Terapon* | *jarbua* | FSCS119-06 | EF607580 | MBCSC:Fish:GD 9085049 | 20.454 109.889 |
|  |  | *jarbua* | FSCS120-06 | EF607579 | MBCSC:Fish:GD 9085050 | 20.454 109.889 |
|  |  | *jarbua* | FSCS121-06 | EF607578 | MBCSC:Fish:GD 9085051 | 20.476 109.825 |
|  |  | *jarbua* | FSCS122-06 | EF607577 | MBCSC:Fish:GD 9085052 | 20.476 109.825 |
|  |  | *jarbua* | FSCS123-06 | EF607576 | MBCSC:Fish:GD 9085053 | 20.454 109.889 |
|  |  | *jarbua* | FSCS244-06 | EF607575 | MBCSC:Fish:GD 9083005 | 21.58 111.815 |
|  |  | *jarbua* | FSCS293-06 | EF607574 | MBCSC:Fish:GD 9087004 | 21.435 111.128 |
|  |  | *jarbua* | FSCS294-06 | EF607573 | MBCSC:Fish:GD 9087005 | 21.42 111.169 |
|  |  | *theraps* | FSCS318-06 | EF607581 | MBCSC:Fish:GD 9088006 | 23.256 116.833 |
| Trichiuridae | *Lepturacanthus* | *savala* | FSCS007-06 | EF607437 | MBCSC:Fish:GD 9085007 | 20.313 109.858 |
|  |  | *savala* | FSCS290-06 | EF607438 | MBCSC:Fish:GD 9087001 | 21.47 111.239 |
|  | *Trichiurus* | *lepturus* | FSCS155-06 | EF607601 | MBCSC:Fish:GD 9081003 | 20.767 110.675 |
|  |  | *lepturus* | FSCS156-06 | EF607600 | MBCSC:Fish:GD 9081004 | 20.767 110.675 |
|  |  | *lepturus* | FSCS1153-11 | JN242480 | MBCSC:Fish:ZH1151892 | 21.125 113.42 |
|  |  | *lepturus* | FSCS1152-11 | JN242479 | MBCSC:Fish:ZP1141241 | 21.333 111.977 |
|  |  | *lepturus* | FSCS1151-11 | JN242478 | MBCSC:Fish:LCG116215 | 23.388 117.449 |
|  |  | *lepturus* | FSCS1150-11 | JN242477 | MBCSC:Fish:XS116952 | 17.311 113.276 |
|  |  | *lepturus* | FSCS1149-11 | JN242476 | MBCSC:Fish:BH1122620 | 21.232 109.377 |
| Uranoscopidae | *Uranoscopus* | *oligolepis* | CFCS018-08 | FJ237963 | MBCSC:Fish:HN SY08334 | 16.035 114.134 |
|  |  | *oligolepis* | CFCS019-08 | FJ237962 | MBCSC:Fish:HN SY08335 | 16.378 112.452 |
|  |  | *oligolepis* | CFCS190-08 | FJ237961 | MBCSC:Fish:HN SY08506 | 17.073 109.736 |

Specimens were morphologically identified by Junbin Zhang and Tingbao Yang. * Samples whose sequence numbers with the suffix “-11” were used for sequence amplifications of cytb, 16S, and 18S.
