# Supplementary material for: Molecular Approach to the Identification of Fish in the South China Sea
Source: PLoS One. 2012 Feb 17;7(2):e30621. doi: 10.1371/journal.pone.0030621 (PMC3281855; doi:10.1371/journal.pone.0030621)
Supplement: Table S2 — Primers (5′-3′) utilized for PCR amplifications and sequencing in this study. (DOCX) [file pone.0030621.s002.docx]

**Table S2** Primers (5′-3′) utilized for PCR amplifications and sequencing in this study.

| **Primer name** | **Primer** | **Amplified genes** |
| --- | --- | --- |
| VF2_t1 | *TGTAAAACGACGGCCAGTCAACCAACCACAAAGACATTGGCAC | COI |
| FishF2_t1 | TGTAAAACGACGGCCAGTCGACTAATCATAAAGATATCGGCAC | COI |
| FishR2_t1 | *CAGGAAACAGCTATGACACTTCAGGGTGACCGAAGAATCAGAA | COI |
| FR1d_t1 | CAGGAAACAGCTATGACACCTCAGGGTGTCCGAARAAYCARAA | COI |
| VF1_t1 | TGTAAAACGACGGCCAGTTCTCAACCAACCACAAAGACATTGG | COI |
| VF1d_t1 | TGTAAAACGACGGCCAGTTCTCAACCAACCACAARGAYATYGG | COI |
| LepF1_t1 | TGTAAAACGACGGCCAGTATTCAACCAATCATAAAGATATTGG | COI |
| VFli_t1 | TGTAAAACGACGGCCAGTTCTCAACCAACCAIAAIGAIATIGG | COI |
| VR1_t1 | CAGGAAACAGCTATGACTAGACTTCTGGGTGGCCRAARAAYCA | COI |
| VR1d_t1 | CAGGAAACAGCTATGACTAGACTTCTGGGTGGCCAAAGAATCA | COI |
| LepR1_t1 | CAGGAAACAGCTATGACTAAACTTCTGGATGTCCAAAAAATCA | COI |
| VRli_t1 | CAGGAAACAGCTATGACTAGACTTCTGGGTGICCIAAIAAICA | COI |
| *Forward primers for cytb* |  |  |
| cytb_F | GGCTGATTCGGAATATGCAYGCNAAYGG | cyt *b* |
| GluRF | TTATTCAACTACAAGAACC | cyt *b* |
| GLuDG | TGACTTGAARAACCAYCGTTG | cyt *b* |
| L14724 | CGAAGCTTGATATGAAAAACCATCGTTG | cyt *b* |
| L15369 | ACAGGMTCAAAYAACCC | cyt *b* |
| L15411 | GATAAAATTYCATTCCACCC | cyt *b* |
| cytb_1F | ATCTCAACHTGATGRAAYTTYGG | cyt *b* |
| CB3 | GGCAAATAGGAARTATCATTC | cyt *b* |
| *Reverse primers for cytb* |  |  |
| cytb_R | GGGAATGGATCGTAGAATTGCRTANGCRAA | cyt *b* |
| H15915 | ACCTCCGATCTYCGGATTACAAGAC | cyt *b* |
| H15973 | TTGGGAGTTAGKGGTRRGAGTT | cyt *b* |
| cytb_1R | TGCAGGAGTDACNADNGGRTT | cyt *b* |
| CB3RF | ATATCATTCTGGCTTAATGTG | cyt *b* |
| 16fiF140 | CGYAAGGGAAHGCTGAAA | 16S |
| 16fiR1524 | CCGGTCTGAACTCAGATCACGTAG | 16S |
| 18S_F | CCACATCCAAGGAAGGCAGCAGGC | 18S |
| 18S_R | CCCGTGTTGAGTCAAATTAA | 18S |

* M13 tails are underlined.
